# Supplementary figures and images for: bfc, a novel serpent co-factor for the expression of croquemort, regulates efferocytosis in Drosophila melanogaster
Source: PLoS Genet. 2021 Dec 3;17(12):e1009947. doi: 10.1371/journal.pgen.1009947 (PMC8673676; doi:10.1371/journal.pgen.1009947)

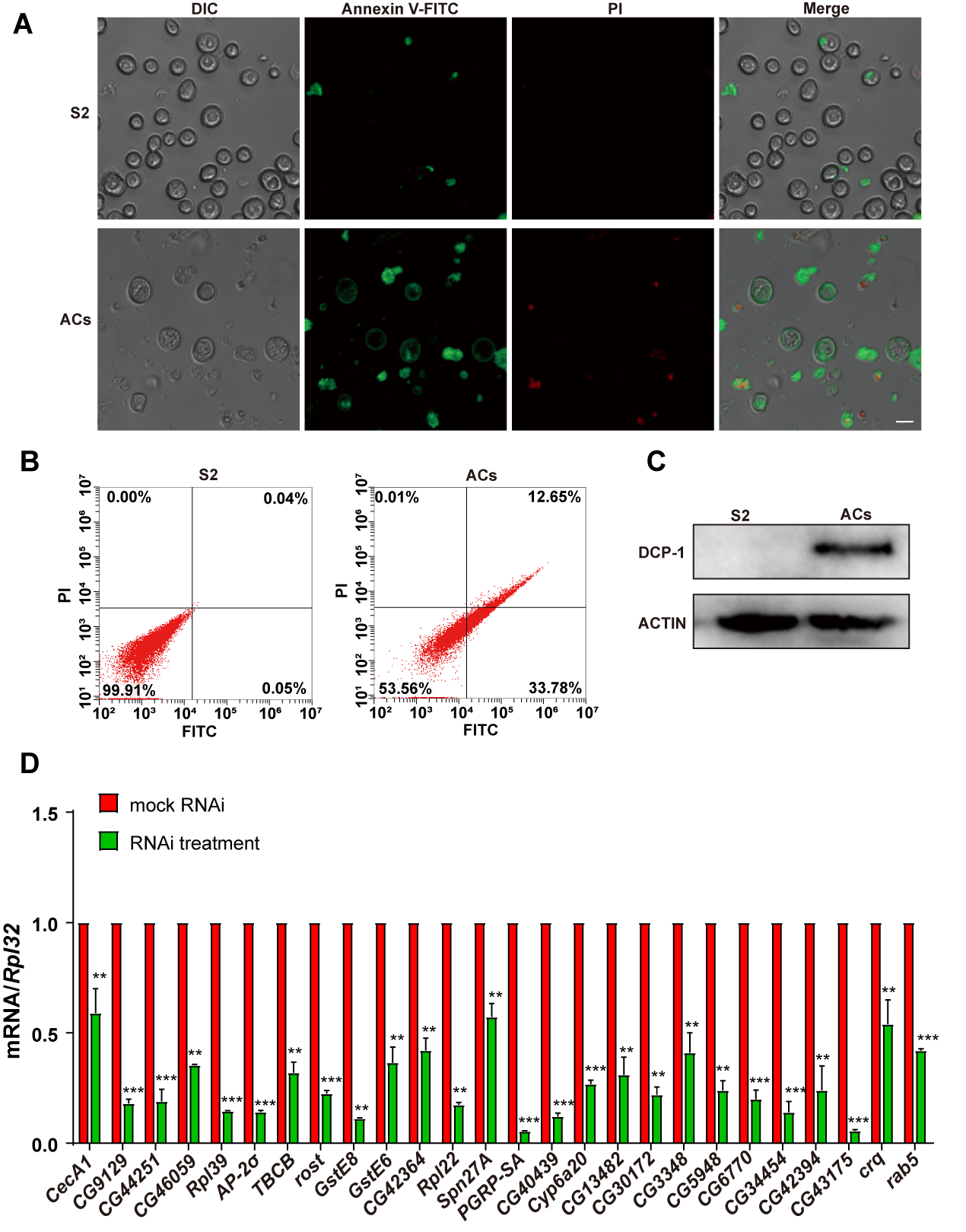

Supplement: S1 Fig — A. S2 cells and ACs were stained with annexin V-FITC and propidium iodide (PI). Scale bar = 20 μm. ACs were obtained from S2 cells after Actinomycin D induction for 18 h, as described in the Materials and Methods section. B. The apoptosis rates of S2 cells and ACs were analyzed by flow cytometry. C. Western blotting detected the Dcp1 protein levels in S2 cells and ACs. Actin was used as the loading control. D. The relative mRNA levels of 24 genes in RNAi-treated S2 cell lines were quantified by qPCR and compared to those of control-treated S2 cells; RpL32 was used as the internal control. Statistical significance was assessed using the two-way ANOVA. (TIF) [file pgen.1009947.s001.tif]

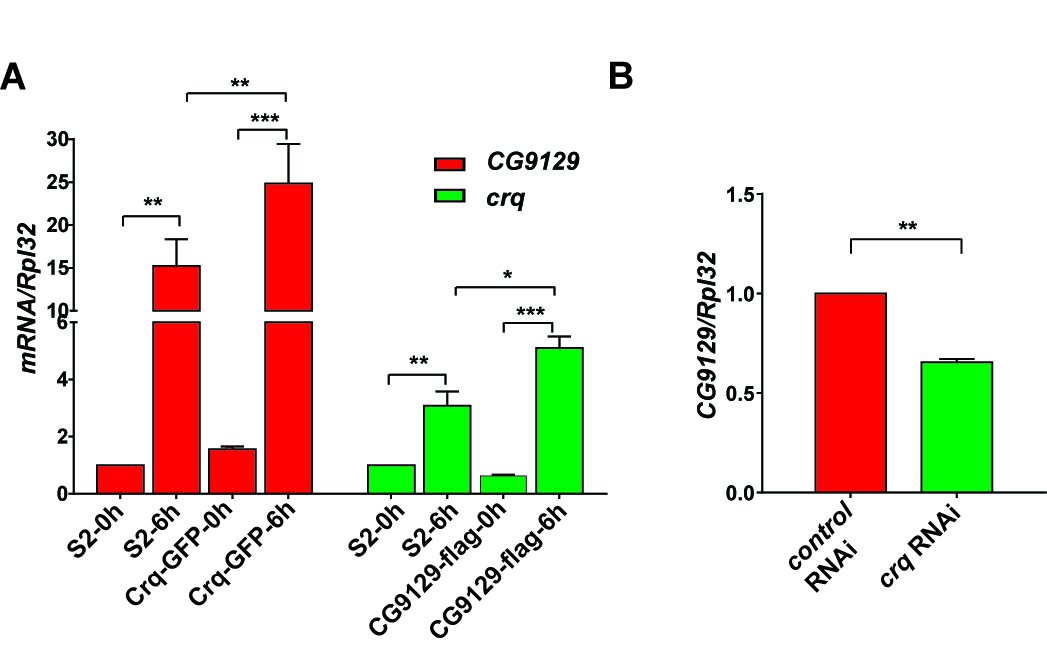

Supplement: S2 Fig — A. The CG9129 mRNA levels were quantified by qPCR in Crq-overexpressing S2 cells (Crq-GFP) and control S2 cells. The crq mRNA levels were also quantified by qPCR in CG9129 over-expressing S2 cells (CG9129-flag) and control S2 cells. 0 h refers to S2 cells in the absence of ACs; 6 h refers to S2 cells co-incubated with ACs for 6 h. RpL32 was used as the internal control. B. The CG9129 mRNA levels were also determined in RNAi-treated S2 cells (control and for the knockdown of crq) after co-incubation with ACs for 6 h, by qPCR. Statistical significance was assessed using the two-way ANOVA (A) or Student’s t-test (B). (TIF) [file pgen.1009947.s002.tif]

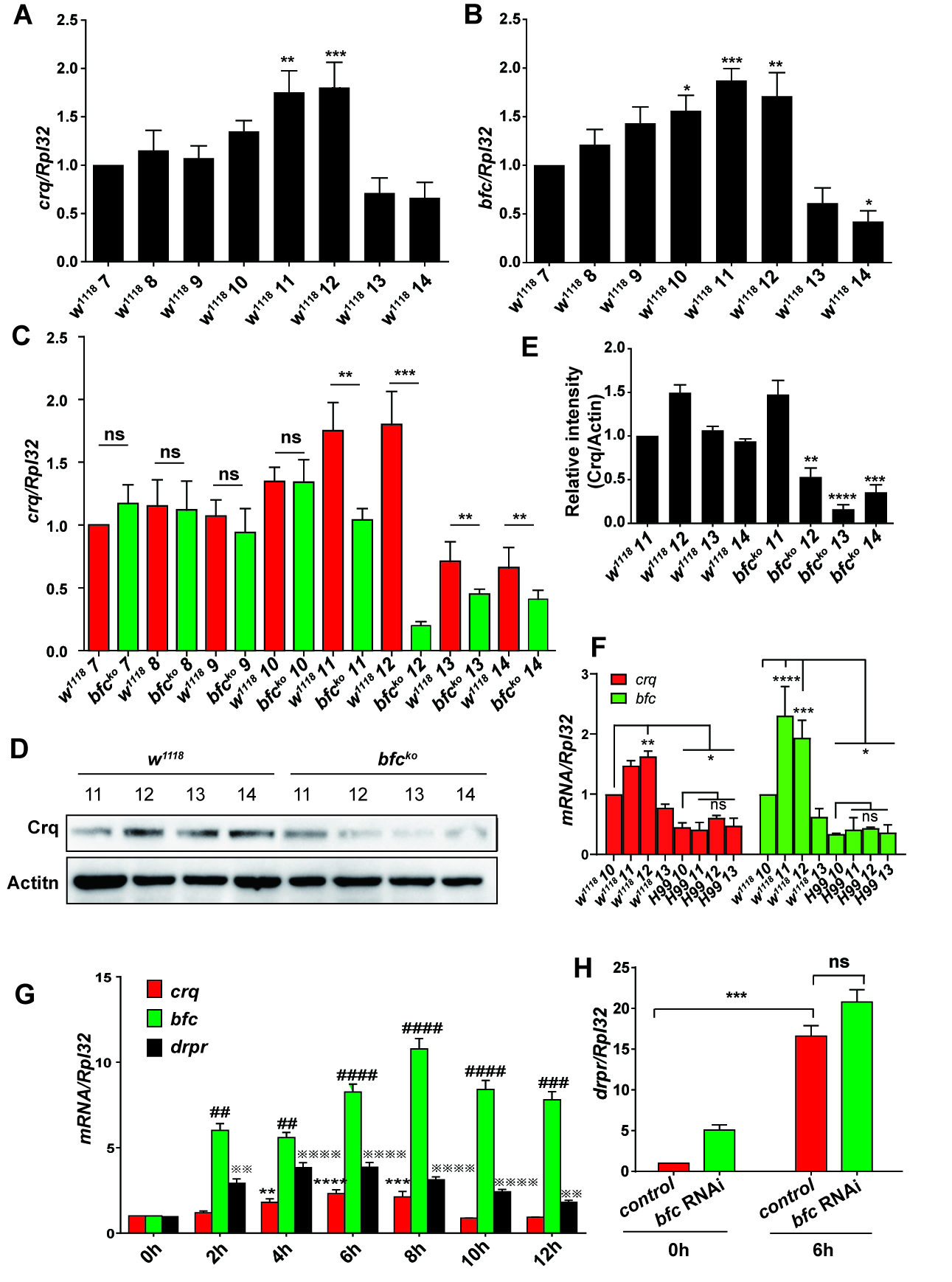

Supplement: S3 Fig — A. The relative crq mRNA levels in stage 7–14 wild type embryos were quantified by qPCR; significant differences were observed compared to the expression in stage 7 embryos. B. The bfc mRNA levels in stage 7–14 wild-type embryos were quantified by qPCR; significant differences were observed compared to the expression in stage 7 embryos. C. The relative crq mRNA levels were quantified and compared in the same stage of wild type and bfcko mutant embryos by qPCR. D. Western blot analysis was used for the detection of the Crq protein levels in stage 11–14 embryos of wild type and bfc mutant flies. Actin was used as the loading control. E. The relative Crq protein levels in stage 11–14 wild type, and bfcko mutant embryos were analyzed via western blotting. The protein levels were normalized to those of actin. Significant differences were observed in comparison with stage 11 wild-type embryos; n = 3. F. The relative crq and bfc mRNA levels were quantified by qPCR in stage to 10–13 wild-type w1118 and H99 (absence of apoptosis) embryos. G. The crq, bfc, and drpr mRNA levels were quantified by qPCR and normalized to the Rpl32 mRNA levels. “*”/“#”/“※” represent the significant differences in the context of crq/bfc/drpr expression, respectively. “##” indicates P<0.01; “###” indicates P<0.001; “####” indicates P<0.0001; “※※” indicates P<0.01. H. The drpr mRNA levels were quantified by qPCR in wild-type S2 cells and bfc-RNAi S2 cells in the presence or absence of ACs. Statistical significance was assessed using the one-way ANOVA(A, B, E, F, H) or two-way ANOVA (C, G). (TIF) [file pgen.1009947.s003.tif]

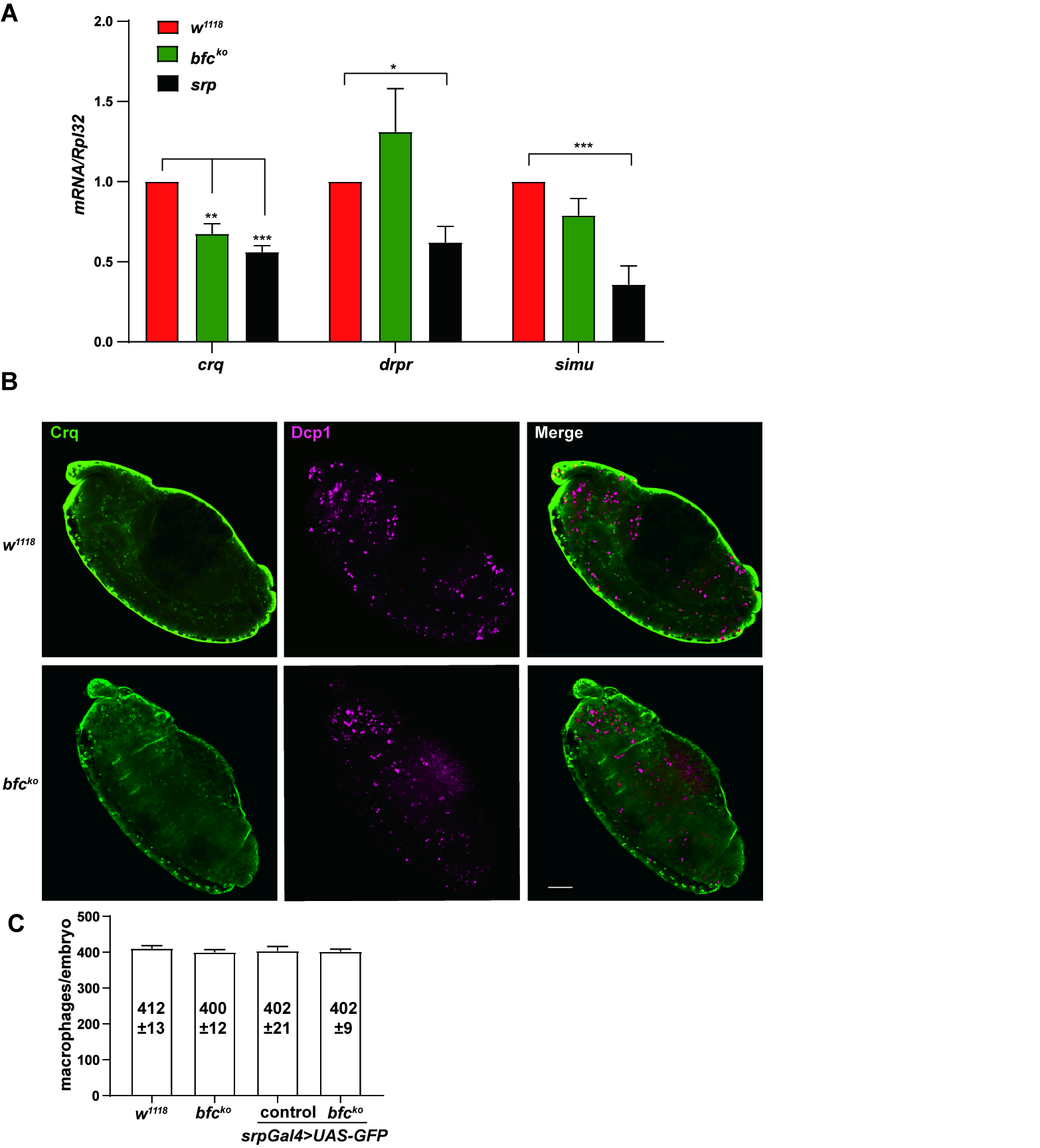

Supplement: S4 Fig — A. The crq, drpr, and simu mRNA levels were quantified by qPCR in stage 13 wild-type, bfcko, and srp mutant embryos. B. Macrophages were stained with anti-CRQ (green) antibodies, and ACs were stained with anti-Dcp1 (magenta) antibodies in stage 13 wild-type and bfcko embryos. Scale bar = 20 μm. C. Graph showing the number of macrophages ± SEM for genotype of wild type, bfcko, srp-Gal4>UAS-GFP and bfcko; srp-Gal4>UAS-GFP. Statistical significance was assessed using the two-way ANOVA (A) or one-way ANOVA (C). (TIF) [file pgen.1009947.s004.tif]

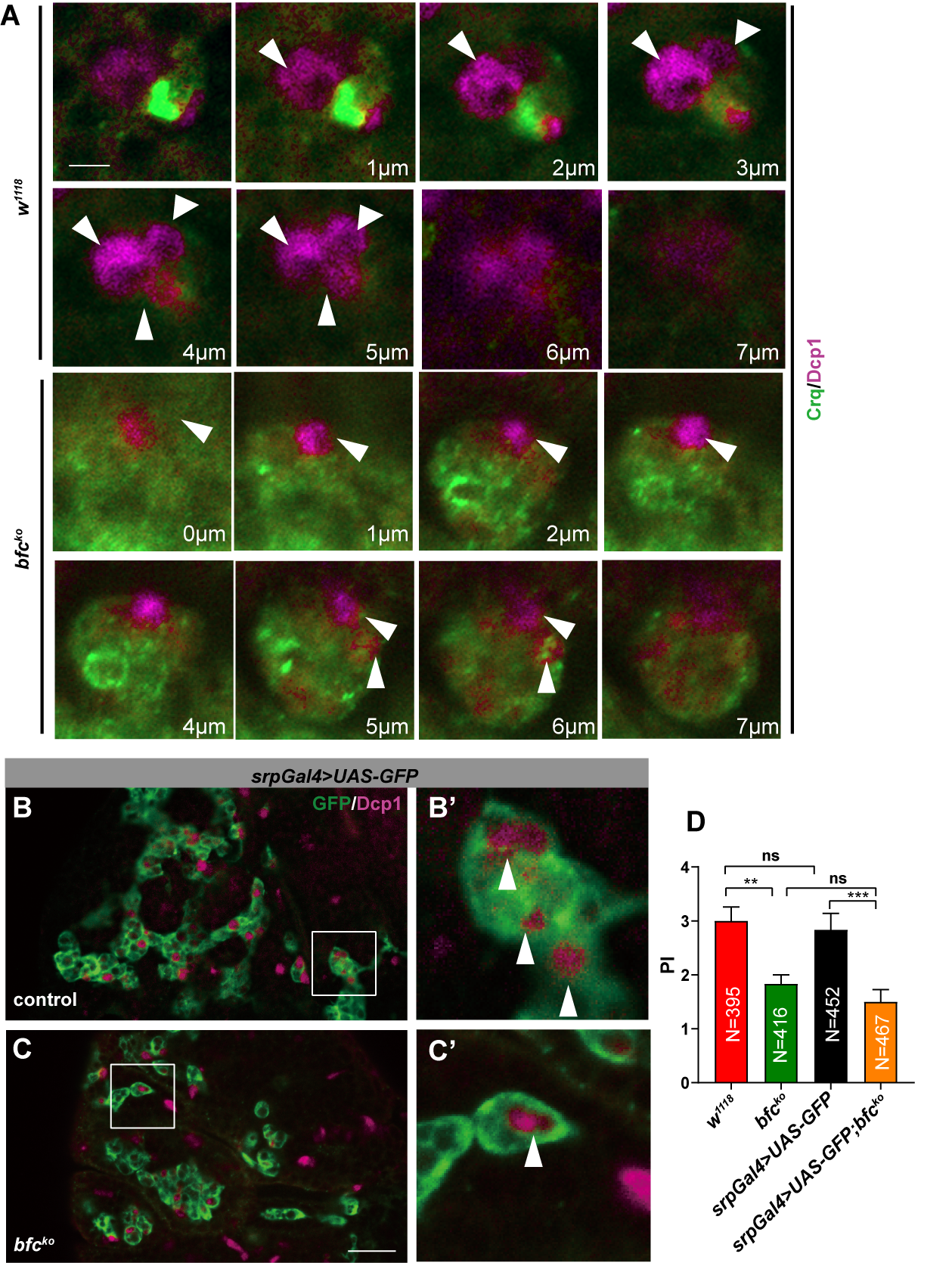

Supplement: S5 Fig — A. Z-stack of eight images from a single macrophage of wild type and bfcko embryos taken at intervals of 1 μm. Scale bar = 5 μm. B-B’. Control srp-Gal4>UAS-GFP embryo macrophages were labeled with srp-Gal4>UAS-GFP (green); anti-Dcp1 was labeled in magenta. C-C’. bfcko; srp-Gal4>UAS-GFP embryo macrophages were labeled as srp-Gal4>UAS-GFP (green); anti-Dcp1 was labeled in magenta. Scale bar = 20 μm. D. Graph showing the mean PIs ± SEM for each genotype (A-C). Statistical significance was assessed using the one-way ANOVA. (TIF) [file pgen.1009947.s005.tif]

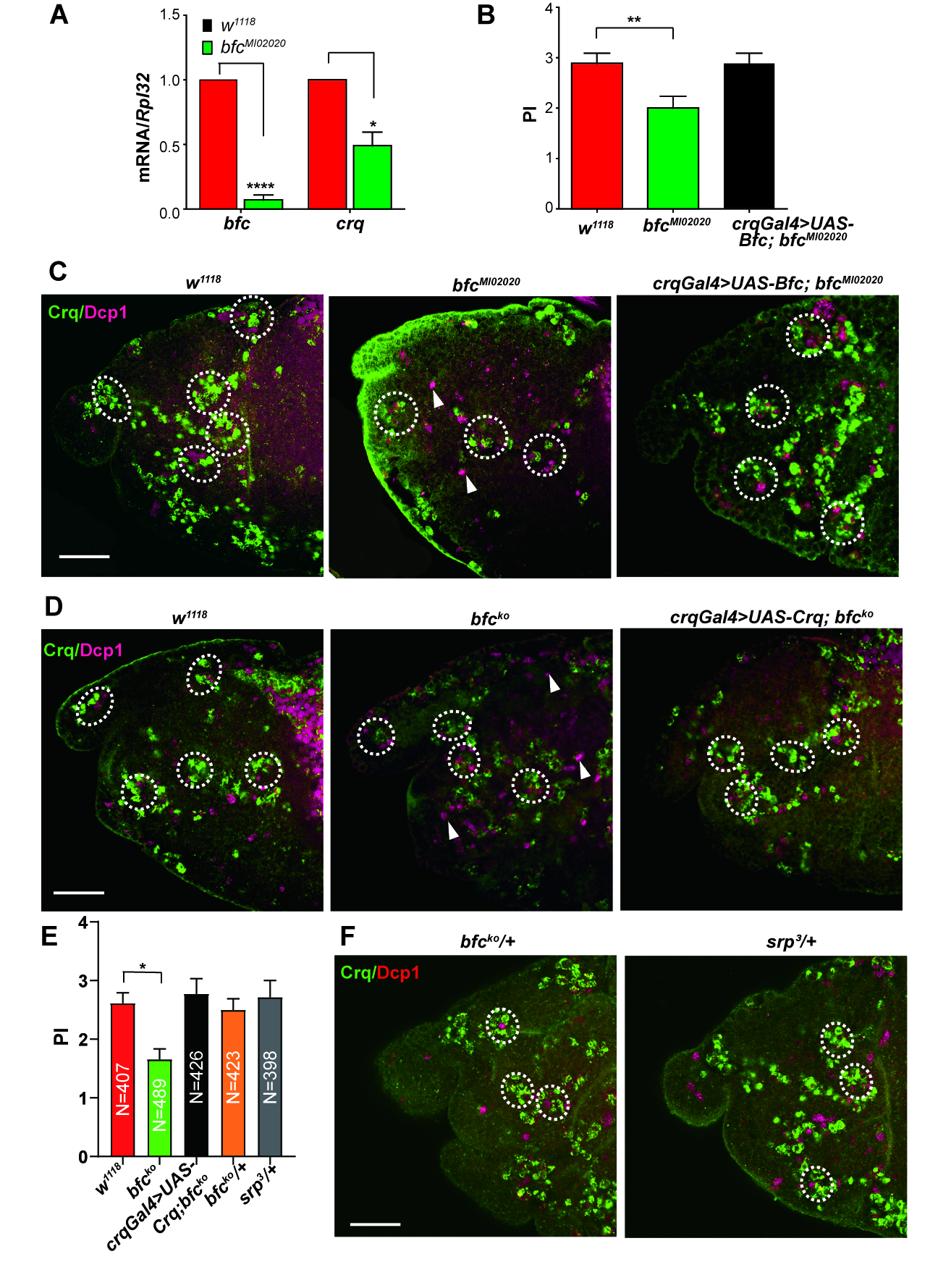

Supplement: S6 Fig — A. The crq and bfc mRNA levels were quantified by qPCR using wild-type and bfcMI02020 embryos. B. Graph showing the mean PI ± SEM for each genotype in (C). C. Anti-CRQ (green) and anti-Dcp1 (magenta) antibodies were used to stain the apoptotic bodies in macrophages of stage 13 wild-type, bfcMI02020, and bfcMI02020 re-expressing UAS-bfc (under the control of a crq-Gal4 driver) embryos. The white arrowheads point to non-engulfed ACs. Scale bar = 20 μm. D. Anti-CRQ (green) and anti-Dcp1 (magenta) antibodies were used to stain the apoptotic bodies in macrophages of stage 13 wild-type, bfcko, and bfcko re-expressing UAS-crq (under the control of a crq-Gal4 driver) embryos. The white arrowheads point to non-engulfed ACs. Scale bar = 20 μm. E. Graph showing the mean PI ± SEM for each genotype in (D). F. Anti-CRQ (green) and anti-Dcp1 (magenta) antibodies were used to stain the apoptotic bodies in heterozygous macrophages of stage 13 bfcko/+ and srp/+ embryos. The white triangles point to non-engulfed ACs. Scale bar = 20 μm. Statistical significance was assessed using the two-way ANOVA (A) or one-way ANOVA (B, E). (TIF) [file pgen.1009947.s006.tif]

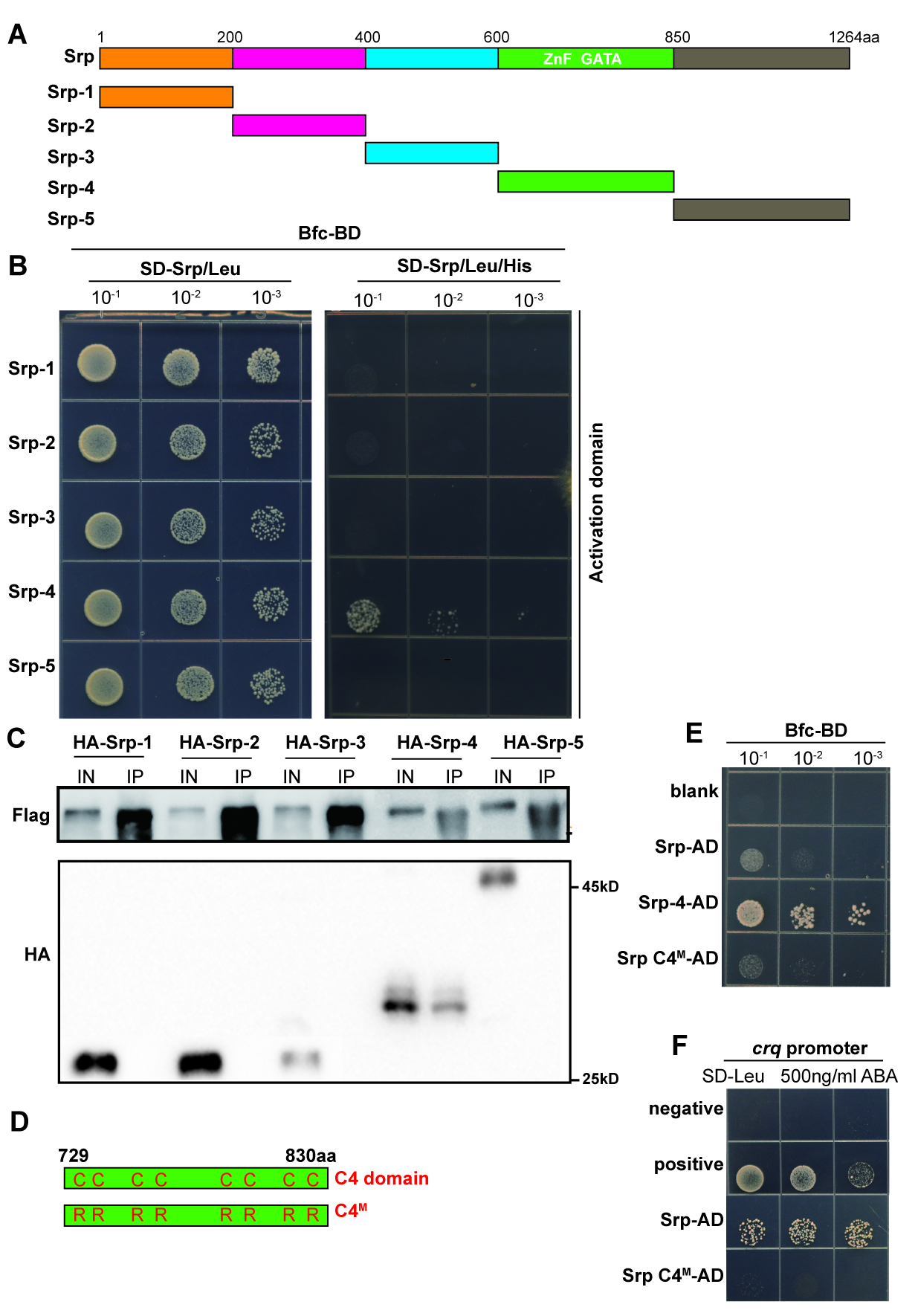

Supplement: S7 Fig — A. Schematic diagram showing the domains of Srp and the truncations generated to examine the potential Bfc-Srp interactions. B and C. The interaction between Bfc and Srp was examined using the yeast two-hybrid (B) and Co-IP assays (C). D. Schematic diagram showing the C4M mutants with the substitution of eight cysteines by arginines in the C4 motif of Srp. E. The yeast two-hybrid assay showing the interaction between Bfc and the C4M Srp mutant. F. The yeast one-hybrid assay shows the lack of interaction between the crq promoter C4M and the C4M Srp mutant. (TIF) [file pgen.1009947.s007.tif]

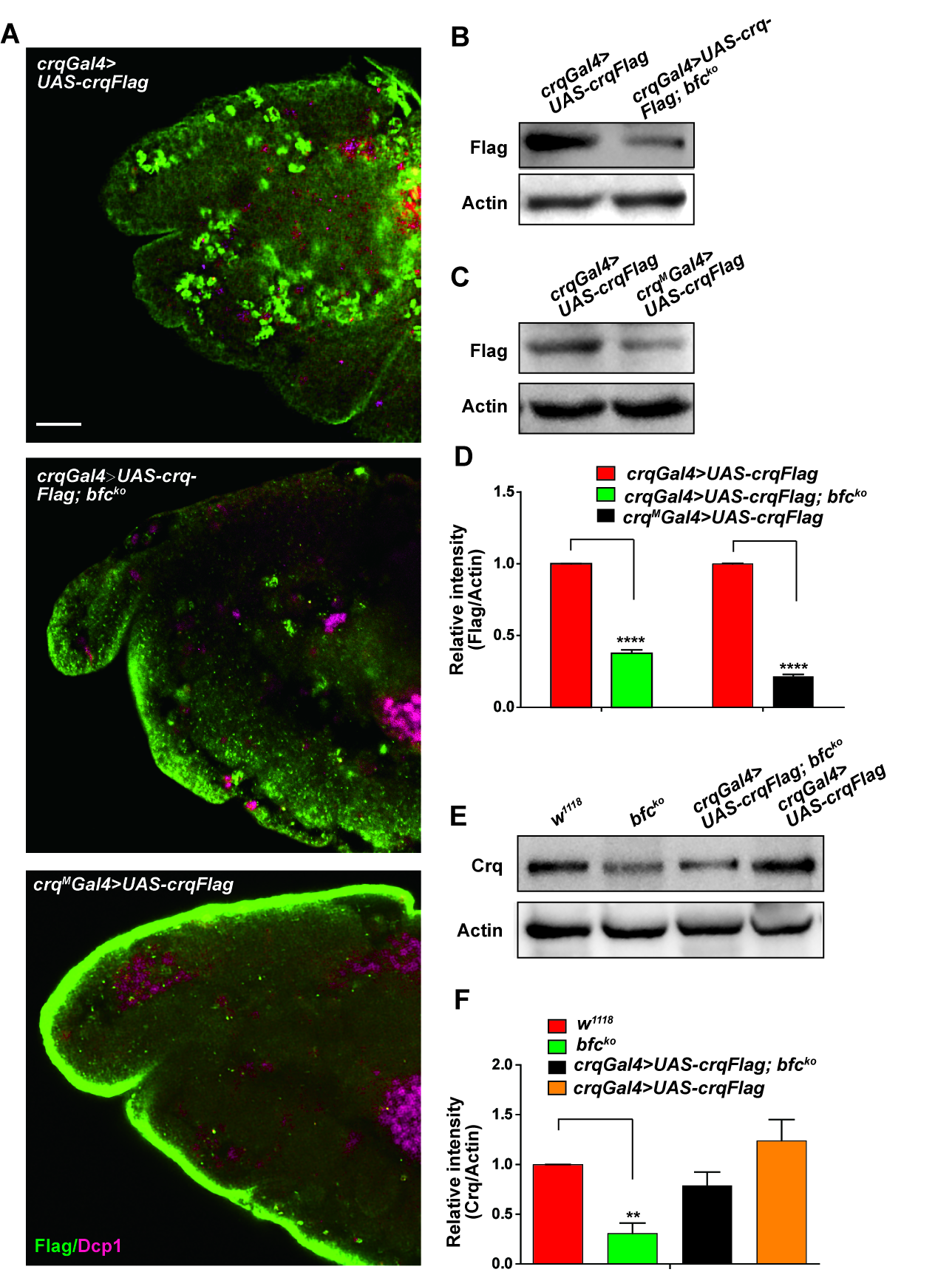

Supplement: S8 Fig — A. Anti-Flag (green) and anti-Dcp1 (magenta) antibodies were used to stain macrophages and ACs of stage 13 wild-type, bfc mutant re-expressing UAS-CrqFlag (under the control of a crq-Gal4 driver), and wild-type re-expressing UAS-CrqFlag (driven by mutated crq-Gal4) embryos. Scale bar = 20 μm. B and C. The Flag protein levels were determined via western blotting for each genotype in (A). Actin was used as the loading control. D. Quantification of the Flag protein levels in (B) and (C) after normalization to those of actin (n = 3). E. The Crq protein levels were detected via western blotting in the context of w1118, bfcko, bfcko mutant re-expressing UAS-CrqFlag (driven by crq-Gal4), and wild-type re-expressing UAS-CrqFlag (driven by crq-Gal4) samples. Actin was used as the loading control. F. Quantification of the Crq protein levels in (E) after normalization to those of actin (n = 3). Statistical significance was assessed using the Student’s t-test. (TIF) [file pgen.1009947.s008.tif]

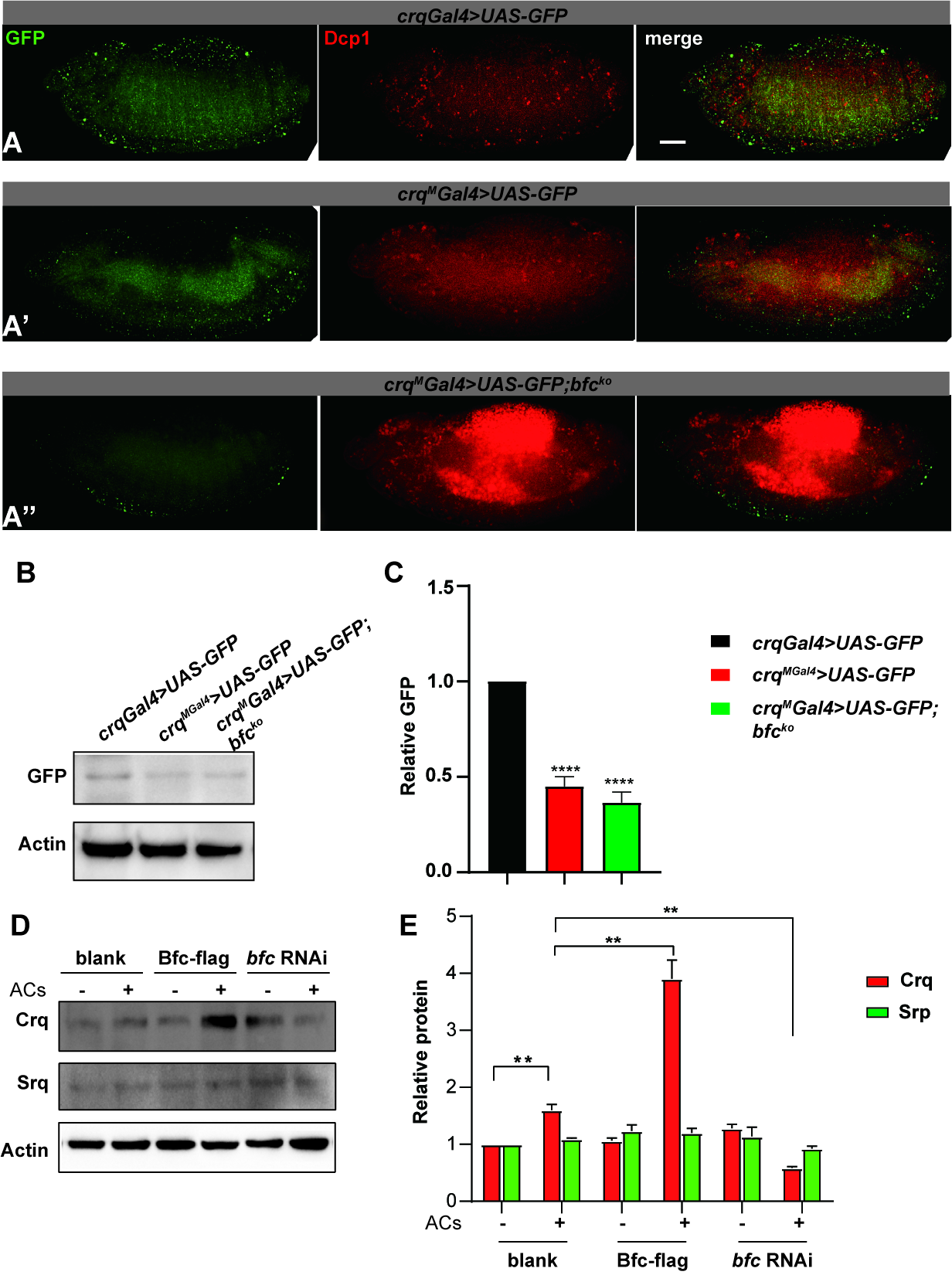

Supplement: S9 Fig — A-A”. Anti-GFP (green) and anti-Dcp1 (Red) antibodies were used to stain macrophages and ACs of stage 13 embryos in crq-Gal4>UAS-GFP(A), crqM-Gal4>UAS-GFP(A’), and crqM-Gal4>UAS-GFP; bfcko(A”). Scale bar = 20 μm. B. The GFP protein levels were determined via western blotting using crq-Gal4>UAS-GFP, crqM-Gal4>UAS-GFP, and crqM-Gal4>UAS-GFP; bfcko embryos. Actin was used as the loading control. C. Quantification of the protein levels in (B) after normalization to actin; n = 3. Statistical significance was assessed using the Student’s t-test. D. The Crq and Srp protein levels were determined via western blot analysis in blank, Bfc-overexpressing, and bfc-RNAi-treated S2 cells in the presence or absence of ACs. Actin was used as the loading control. E. Quantification of the protein levels in (D) after normalization to actin; n = 3. Statistical significance was assessed using the one-way ANOVA(C) and two-way ANOVA(E). (TIF) [file pgen.1009947.s009.tif]

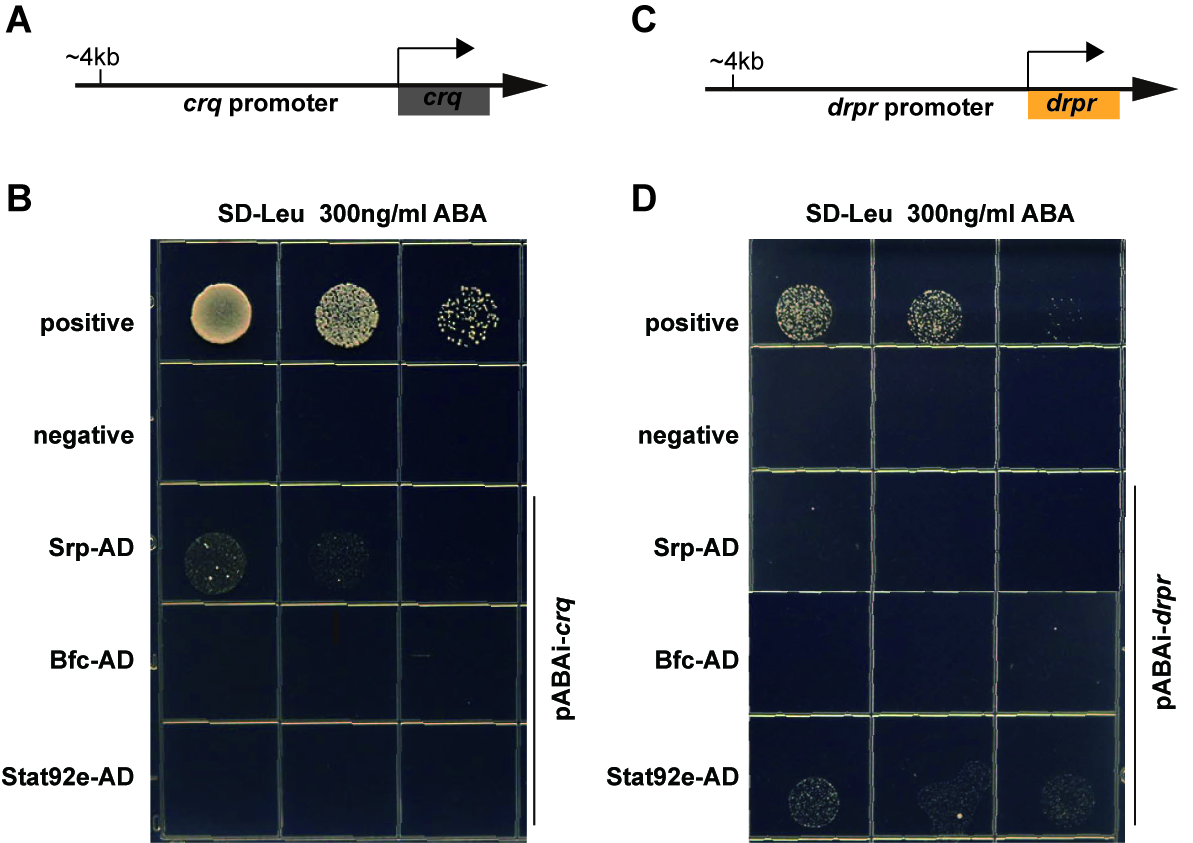

Supplement: S10 Fig — A. Schematic diagram showing the domain of the crq promoter used for the yeast one-hybrid assay. B. The yeast one-hybrid assay shows the interactions between Srp-AD, Bfc-AD, Stat92e-AD, and the crq promoter. C. Schematic diagram showing the domain of the drpr promoter used for the yeast one-hybrid assay. D. The yeast one-hybrid shows the interactions between Srp-AD, Bfc-AD, Stat92e-AD, and the drpr promoter. (TIF) [file pgen.1009947.s010.tif]
