# Supplementary material for: bfc, a novel serpent co-factor for the expression of croquemort, regulates efferocytosis in Drosophila melanogaster
Source: PLoS Genet. 2021 Dec 3;17(12):e1009947. doi: 10.1371/journal.pgen.1009947 (PMC8673676; doi:10.1371/journal.pgen.1009947)
Supplement: S1 Table — Representative genes were selected across a range of expression levels. (DOCX) [file pgen.1009947.s012.docx]

**S1 Table. The RNA-seq data of 50 filtered out genes**

| Identified genes | S2-0h normalize | S2-6h normalize | S2-12h normalize | FoldChange of S2-6h to S2-0h | FoldChange of S2-12h to S2-6h | FoldChange of S2-12h to S2-0h |
| --- | --- | --- | --- | --- | --- | --- |
| *scf* | 516.845 | 1278.295 | 912.491 | 2.473 | 0.714 | 1.766 |
| *CG17440* | 0.030 | 2.511 | 1.253 | 84.951 | 0.499 | 42.393 |
| *CG17258* | 0.355 | 6.164 | 3.760 | 17.376 | 0.610 | 10.598 |
| *CG6770* | 115.525 | 612.742 | 346.593 | 5.304 | 0.566 | 3.000 |
| *CG13196* | 0.030 | 3.881 | 1.611 | 131.288 | 0.415 | 54.505 |
| *Cyp6a20* | 96.192 | 263.984 | 187.976 | 2.744 | 0.712 | 1.954 |
| *CG11475* | 0.414 | 3.424 | 1.611 | 8.274 | 0.471 | 3.893 |
| *CG9129* | 0.030 | 2.663 | 0.895 | 90.099 | 0.336 | 30.281 |
| *CG13482* | 32.754 | 113.310 | 83.247 | 3.459 | 0.735 | 2.542 |
| *CG7900* | 4.139 | 12.328 | 8.414 | 2.979 | 0.683 | 2.033 |
| *CG9444* | 0.946 | 6.240 | 3.043 | 6.597 | 0.488 | 3.217 |
| *CG8907* | 0.296 | 9.664 | 4.655 | 32.693 | 0.482 | 15.746 |
| *CG5246* | 0.177 | 2.511 | 0.895 | 14.158 | 0.356 | 5.047 |
| *CG5948* | 0.030 | 2.663 | 1.253 | 90.099 | 0.471 | 42.393 |
| *SP10* | 1.123 | 22.449 | 15.038 | 19.984 | 0.670 | 13.387 |
| *CG3348* | 1.537 | 11.947 | 4.118 | 7.772 | 0.345 | 2.679 |
| *CG13315* | 0.473 | 7.762 | 2.685 | 16.411 | 0.346 | 5.678 |
| *CG30172* | 0.355 | 6.392 | 2.685 | 18.020 | 0.420 | 7.570 |
| *CG33099* | 3.725 | 32.722 | 11.995 | 8.785 | 0.367 | 3.220 |
| *GstE9* | 6.799 | 19.177 | 12.174 | 2.821 | 0.635 | 1.791 |
| *GstE8* | 15.135 | 66.358 | 51.738 | 4.384 | 0.780 | 3.418 |
| *GstE6* | 187.772 | 576.443 | 357.513 | 3.070 | 0.620 | 1.904 |
| *CG34454* | 3.311 | 9.056 | 6.266 | 2.735 | 0.692 | 1.893 |
| *CG42364* | 0.946 | 4.185 | 2.506 | 4.425 | 0.599 | 2.650 |
| *CR43687* | 0.059 | 4.185 | 1.253 | 70.792 | 0.299 | 21.196 |
| *CG44251* | 9.164 | 25.112 | 15.933 | 2.740 | 0.634 | 1.739 |
| *CG46059* | 0.828 | 3.881 | 1.969 | 4.689 | 0.507 | 2.379 |
| *GstS1* | 19.156 | 49.159 | 28.107 | 2.566 | 0.572 | 1.467 |
| *rost* | 50.786 | 117.876 | 79.129 | 2.321 | 0.671 | 1.558 |
| *crq* | 136.868 | 244.351 | 192.631 | 1.785 | 0.788 | 1.407 |
| *Rpl39* | 552.437 | 1144.134 | 877.402 | 2.071 | 0.767 | 1.588 |
| *Spn27A* | 36.537 | 73.967 | 58.541 | 2.024 | 0.791 | 1.602 |
| *Tsp42Ed* | 25.718 | 55.628 | 38.848 | 2.163 | 0.698 | 1.511 |
| *CG12112* | 57.703 | 120.920 | 95.062 | 2.096 | 0.786 | 1.647 |
| *PGRP-SA* | 76.800 | 76.800 | 129.614 | 2.411 | 1.688 | 1.688 |
| *Cyp4p3* | 7.568 | 15.600 | 11.100 | 2.061 | 0.712 | 1.467 |
| *CG12133* | 27.906 | 58.672 | 39.923 | 2.103 | 0.680 | 1.431 |
| *TBCB* | 112.864 | 232.632 | 182.964 | 2.061 | 0.786 | 1.621 |
| *AP-2σ* | 56.343 | 103.189 | 79.487 | 1.831 | 0.770 | 1.411 |
| *CG40439* | 73.666 | 132.791 | 105.625 | 1.803 | 0.795 | 1.434 |
| *GstE3* | 97.374 | 305.305 | 163.987 | 3.135 | 0.537 | 1.684 |
| *CG42394* | 45.110 | 102.885 | 71.073 | 2.281 | 0.691 | 1.576 |
| *CG42709* | 34.113 | 64.836 | 50.843 | 1.901 | 0.784 | 1.490 |
| *CG43175* | 172.282 | 398.449 | 277.489 | 2.313 | 0.696 | 1.611 |
| *CaBP1* | 713.249 | 1284.535 | 1020.085 | 1.801 | 0.794 | 1.430 |
| *CecA1* | 32.281 | 111.864 | 59.436 | 3.465 | 0.531 | 1.841 |
| *Pu* | 1.892 | 8.295 | 5.371 | 4.384 | 0.647 | 2.839 |
| *LysX* | 3.370 | 8.903 | 6.266 | 2.642 | 0.704 | 1.859 |
| *Cyp4e1* | 0.059 | 3.729 | 1.074 | 63.070 | 0.288 | 18.168 |
| *RpL22* | 112.155 | 325.091 | 230.763 | 2.899 | 0.710 | 2.058 |
