# Supplementary material for: bfc, a novel serpent co-factor for the expression of croquemort, regulates efferocytosis in Drosophila melanogaster
Source: PLoS Genet. 2021 Dec 3;17(12):e1009947. doi: 10.1371/journal.pgen.1009947 (PMC8673676; doi:10.1371/journal.pgen.1009947)
Supplement: S2 Table — (DOCX) [file pgen.1009947.s013.docx]

**S2 Table. qPCR primers used in Figure 2**

| Gene | Forward | Reverse | Reference |
| --- | --- | --- | --- |
| *Rpl32* | GACGCTTCAAGGGACAGTATCTG | AAACGCGGTTCTGCATGAG | [1] |
| *crq* | GTGGAATGAAGCCGGAGAAG | GATGCCAATTCGGAGGAGAG | [1] |
| *CG7900* | TTATCGTCCCAAATCCAAGAGG | AACCAACTCCACCGATGTTAG | This paper |
| *CG6770* | CGATGAGTACGAGCACTACAAC | TCAGCTTGGTCAGAATCTTGC | This paper |
| *CG34454* | ATTTCTGAACTGCTTTGGTAACTG | CCTCTTCGAACTATTTGAATGTCAG | This paper |
| *GstE6* | GATGCCCTATACCCGAAAGATC | CTCCTTGGGTACTTTCGTCTG | This paper |
| *CecA1* | CAACATCTTCGTTTTCGTCGC | TGTTGAGCGATTCCCAGTC | This paper |
| *CaBP1* | TTGAAGGGAGTCGTGAAAGTG | TTGAAGGGAGTCGTGAAAGTG | This paper |
| *scf* | GGGATTCCTACATGCAGACTG | ATTGTCATCCAAGTCCTGGTC | This paper |
| *Cyp6a20* | GGGAAATTGGATTACGACAGC | TGGTGTGCTGGTAGTGTTG | This paper |
| *Cyp4e1* | TTGCGATACAGCTATGGGTG | TCAGGAGCAAAGTGAAAGAGG | This paper |
| *CG3348* | AGCTCTACTCCGAGGAACTC | ATTGACTGATCTTGGCTCGG | This paper |
| *CG42709* | CCTCCCATTGTTCGCATTTAG | CTGATTGGTCTGGCTCTCG | This paper |
| *CG43175* | CGGTGAATCTTTGGTTTGCTG | ATCAGGGACGCATTGGTG | This paper |
| *Rpl22* | GAGGATAGCATCATGGATGTGG | TGGAAAAGTGAACGTCGGAG | This paper |
| *LysX* | ACAATGGCTGCGATGTGAGT | CCAGGACCTTTAGGGCACAT | This paper |
| *CG9444* | CGACATGGGTTTCGTCAATG | ATGTGGTATTCATCTGGGTGG | This paper |
| *CG17258* | TTATTGAGAACCAGCGGAAGG | AGGAGTTCTTTGTGATCAACCG | This paper |
| *CG33099* | TTATAAGAACCCGTGGCAGAC | TGTATCAGTTCATAGGCGCTG | This paper |
| *rost* | TGTATCAGTTCATAGGCGCTG | CCAGAATGGGATACACGTAGTG | This paper |
| *TBCB* | CGCCTTCGAGGTCAAGTTAG | CCCGTCAGTATCTCAAGTTTGG | This paper |
| *AP-2σ* | GCCAAGCACACCAACTTTG | CAGAAGTACAGACCCGCATAG | This paper |
| *CG40439* | TCGAGCATTGGGAGTTCTTG | GCATGTGACGGTATTGGAAAAC | This paper |
| *GstE3* | GCCGGAATGATTCTCTATACCC | GAATCTCCGTCTTGTTCTCCC | This paper |
| *CG42394* | GCAAGTGTCTGGTCGGAAA | GTAGACAAGAAAGTAGACGGCC | This paper |
| *CG12133* | GAAATGGAAGACACTACAACGAC | CCGGCAATTTGAAAAGGGAAG | This paper |
| *CG8907* | TCCTCATTTTCTCAGTGCCC | TCCCTTGCTTAGCGTCTTTAG | This paper |
| *GstE8* | CGTCAAAATAAACACCCTGGC | TGTGAGTCCCAGATGAAATGG | This paper |
| *CG44251* | ATACGAATTCCAGAAGCCCAG | GTAGTGTCCTCGCCCATAGTA | This paper |
| *CG13315* | ACTACGCGCTACATTGCC | GTTCTGCATCCGTACACTCTG | This paper |
| *SP10* | CAGGATCGAACGGGAAGTC | TATCTTTCGTGAACCCGGATC | This paper |
| *GstE9* | CGGTGAGCACAAGACGAAG | TGAACTTGCCATCGTCCTCC | This paper |
| *Spn27A* | AGTTCCATTTCGACTACCAGC | GTTAGACACCTTTACTTTGCCG | This paper |
| *Tsp42Ed* | GTCTTCCTGGTAGCCTTCATG | CACCCACACGTAGATAATGAGAG | This paper |
| *CG12112* | CGCCACCGAGAATAGTCATATC | TTCCGTAAGTGTCTTAACCGC | This paper |
| *PGRP-SA* | CGAGCTGGACTTCAACGATAT | CCACAAAGTTGCCGATAAAGG | This paper |
| *CG17440* | TGAGCGCAAGTCCAAGATAG | TCTCGGTGATCTCTATACAGTCG | This paper |
| *CG9441* | CTGTACTTCACCAAGGGCTAC | CGTAGAAGGGCACCAGATG | This paper |
| *GstS1* | CGTAGAAGGGCACCAGATG | ATCAGGGACGCATTGGTG | This paper |
| *Rpl39* | AACAGATCCGTTCCCCAATG | CCAGTGACGGCGCTTAG | This paper |
| *CG9129* | TGCTACGAGAGGAATAGGGAG | ATTGCACACCAGTCTCAGG | This paper |
| *CG30172* | ATCCTGGGCAAGAGCGAATG | GGAGCAGTTCCTGCATTTCC | This paper |
| *CG46059* | GCGGATGCTACAACTGCT | GGCATAAATGAGCACTGGATG | This paper |
| *CG13196* | TTAAGAACCAGCGGAGATTCG | GCATTTTGATCGCCTCCATG | This paper |
| *CG11475* | CACGCACAAGGACTTTAACTG | TCGCACAGGATAAAGGAACG | This paper |
| *CG5246* | AGATCCATTGCAGTCACGAC | GTTAGCTTATCTCCCACCTTCG | This paper |
| *CG13482* | CAAGCAACGAGAGCAGTTTTC | TCCACCTCCACCCAAATTC | This paper |
| *CG5948* | CTCCTCAGCCGTCGTATTTG | GTCACCCTAATGTCCGAGTTG | This paper |
| *CG42364* | TGGGAAAGCGAGTGTATCAC | GTACAAGGATCGTGCACAAAG | This paper |
| *Cyp4p3* | CCTGTTTCCTTCTGTTCCCG | TGGCAAGATGAGACCGTTG | This paper |

**Reference**

1. Guillou A, Troha K, Wang H, Franc NC, Buchon N. The Drosophila CD36 Homologue croquemort Is Required to Maintain Immune and Gut Homeostasis during Development and Aging. PLoS Pathog. 2016;12(10):e1005961. Epub 2016/10/26. doi: 10.1371/journal.ppat.1005961. PubMed PMID: 27780230; PubMed Central PMCID: PMCPMC5079587.
