# Supplementary material for: bfc, a novel serpent co-factor for the expression of croquemort, regulates efferocytosis in Drosophila melanogaster
Source: PLoS Genet. 2021 Dec 3;17(12):e1009947. doi: 10.1371/journal.pgen.1009947 (PMC8673676; doi:10.1371/journal.pgen.1009947)
Supplement: S3 Table — (DOCX) [file pgen.1009947.s014.docx]

**S3 Table. dsRNA primers used in Figure 2**

| Gene | Forward | Reverse |
| --- | --- | --- |
| *CecA1* | TGAATCGACGCTTTTCTGTG | GATATGCTGTGCCCCGTG |
| *CG9129* | AGGAGTTCTATTCACAGAACATA | GCGCCGCTCCCTATTC |
| *CG44251* | ATATGGATATGGTTTGGCCG | AGGACGGCGCCATGATATAC |
| *CG46059* | TTAACATTTATAAAATTCGTTT | ATGTGCTGCGGACCCTGT |
| *Rpl39* | CACACAAGTCGTTCAGAATAA | ACTTGGTACGCCTCCAGT |
| *AP-2σ* | GCAACTTCAAGATCGTCTAC | CTCCAGCGAATTTAGCGT |
| *TBCB* | GATACTGACGGGCGGATG | CATCTGCCACCCAGTACAC |
| *rost* | CCCATTCTGGATTGGAACAA | GCGACTCCCTTTTCAGTGTC |
| *GstE8* | CGTCATAGAGATTTACGACTTT | TGTATCGATGACCACGAATAC |
| *GstE6* | AAGGAGCATATCGAAGTCAG | GTTGATTGCCAGCAATGTAAT |
| *CG42364* | AAGCGAGTGTATCACATGTTCG | TAAATAGCTTTGCCCTCTCTGC |
| *Rpl22* | CGGCCAAGAACGTGAAG | GCCACATCCATGATGCTATC |
| *Spn27A* | AAGACACTTAATTCCTTCAAGAA | AGGTTCTTGACCAAGTCATG |
| *PGRP-SA* | CTGCGGCTGTTATCAGTGAA | CTGCGGCTGTTATCAGTGAA |
| *CG40439* | TTACCTGATTGGAAATTTTACTGA | TACCTGGCTAATAAATTTTCTAATA |
| *Cyp6a20* | GTTGTTCCGAAATGGCTGTT | AACTCGCGAATCAGTACGGT |
| *CG13482* | ATGTCGCCGCCACATCA | ATGATGATGATGTCCATGGTG |
| *CG30172* | ATTCAGTTATACTATACCGTCTC | AGTTGAAGTCCCAGTTGGTC |
| *CG3348* | AGTTGAAGTCCCAGTTGGTC | TGTGGACACCGATTGACTGA |
| *CG5948* | TAATGTGGACACCAAGGATGA | ATTTTCCATTGCAGGTGGTG |
| *CG6770* | CAAAAAGCAGACACCAACCA | AGCTTGGTCAGAATCTTGCG |
| *CG34454* | TATGCTTAGTGCAGCTGATT | ACAGAAACGAGCACAATTAAAT |
| *CG42394* | ATCAGTGGATTGCTGGTGGT | CAGCGGTGTTCGTTTCTGTA |
| *CG43175* | TCTGCTCTAATGGGACTAAG | GATTTATTCGGTCCACAAGG |
| *crq* | TAAACTTTATGCTGAACCATGAA | ATCAAATCGCGCCTCATC |
| *drpr* | GGACCCGGCTGCAAGC | TTTCGGTTTTTAGATTGGACAC |
| *rab5* | TCCTGGCCAGCCGTGT | GGCAACCACTCCACGCA |

Add T7 promoter *taatacgactcactatagggaga* to each primer 5’end.
