# Supplementary material for: bfc, a novel serpent co-factor for the expression of croquemort, regulates efferocytosis in Drosophila melanogaster
Source: PLoS Genet. 2021 Dec 3;17(12):e1009947. doi: 10.1371/journal.pgen.1009947 (PMC8673676; doi:10.1371/journal.pgen.1009947)
Supplement: S4 Table — (DOCX) [file pgen.1009947.s015.docx]

**S4 Table. Recombinant DNA used in this paper**

| **Recombinant DNA** | | |
| --- | --- | --- |
| **Plasmid names** | **Application** | **Source** |
| pAC5.1-Crq-GFP | Over-express Crq-GFP in S2 cells | This paper |
| pAC5.1-Bfc-Flag | Over-express Bfc-flag in S2 cells | This paper |
| pAC5.1-Bfc-GFP | Over-express Bfc-GFP in S2 cells | This paper |
| pAC5.1-Srp-mCherry | Over-express Srp-mCherry in S2 cells | This paper |
| pAC5.1-HA-Srp | Over-express HA-Srp in S2 cells,used for CO-IP and luciferase assay | This paper |
| pAC5.1-HA-Srp-1 | Over-express HA-Srp in S2 cells,used for CO-IP | This paper |
| pAC5.1-HA-Srp-2 | Over-express HA-Srp in S2 cells,used for CO-IP | This paper |
| pAC5.1-HA-Srp-3 | Over-express HA-Srp in S2 cells,used for CO-IP | This paper |
| pAC5.1-HA-Srp-4 | Over-express HA-Srp in S2 cells,used for CO-IP | This paper |
| pAC5.1-HA-Srp-5 | Over-express HA-Srp in S2 cells,used for CO-IP | This paper |
| pAC5.1-TK | Express seapanzy under Ac5 promoter, used for luciferase assay | This paper |
| pGL3-*crq*Pro | Express firefly luciferase under *crq* promoter, used for luciferase assay | This paper |
| pMD18T | Used for generating sgRNA plasmid expressed in fly | [1] |
| PCR8 | Used for generating sgRNA plasmid expressed in fly | [1] |
| attB | Used for generating sgRNA plasmid expressed in fly | [1] |
| pABAi-*crq* | Integrating *crq* promoter to yeast, used for yeast one-hybrid | This paper |
| pABAi-*crq-1* | Integrating *crq-1* promoter to yeast, used for yeast one-hybrid | This paper |
| pABAi-*crq-2* | Integrating *crq-2* promoter to yeast, used for yeast one-hybrid | This paper |
| pABAi-*crq-3* | Integrating *crq-3* promoter to yeast, used for yeast one-hybrid | This paper |
| pABAi-*crq-4* | Integrating *crq-4* promoter to yeast, used for yeast one-hybrid | This paper |
| pABAi-*drpr* | Integrating *drpr* promoter to yeast, used for yeast one-hybrid | This paper |
| pET28a-Bfc | Express Bfc-his in *E. coli* | This paper |
| pET28a-Crq | Express Crq-his in *E. coli* | This paper |
| pGEX KG-Srp | Express GST-Srp in *E. coli* | This paper |
| pGADT7-Stat92e | Stat92e-AD, used for yeast two-hybrid | This paper |
| pGADT7-Bfc | Bfc-AD, used for yeast two-hybrid | This paper |
| pGADT7-Srp | Srp-AD, used for yeast two-hybrid | This paper |
| pGADT7-Srp-1 | Srp-1-AD, used for yeast two-hybrid | This paper |
| pGADT7-Srp-2 | Srp-2-AD, used for yeast two-hybrid | This paper |
| pGADT7-Srp-3 | Srp-3-AD, used for yeast two-hybrid | This paper |
| pGADT7-Srp-4 | Srp-4-AD, used for yeast two-hybrid | This paper |
| pGADT7-Srp-5 | Srp-5-AD, used for yeast two-hybrid | This paper |
| pGADT7-Srp^C4^ | Srp^C4^-AD, used for yeast two-hybrid and yeast one-hybrid | This paper |
| pGBKT7-Bfc | Bfc-BD, used for yeast two-hybrid | This paper |

1. Du G, Xiong L, Li X, Zhuo Z, Zhuang X, Yu Z, et al. Peroxisome Elevation Induces Stem Cell Differentiation and Intestinal Epithelial Repair. Dev Cell. 2020;53(2):169-84 e11. Epub 2020/04/04. doi: 10.1016/j.devcel.2020.03.002. PubMed PMID: 32243783.
