# Supplementary material for: bfc, a novel serpent co-factor for the expression of croquemort, regulates efferocytosis in Drosophila melanogaster
Source: PLoS Genet. 2021 Dec 3;17(12):e1009947. doi: 10.1371/journal.pgen.1009947 (PMC8673676; doi:10.1371/journal.pgen.1009947)
Supplement: Attachment — Submitted filename: Zheng et al. Bfc Review.pdf [file pgen.1009947.s017.pdf]

Bfc, a novel Serpent co-factor for the expression of Croquemort, regulates efferocytosis in Drosophila melanogaster

Zheng and colleagues have identified a new gene called bfc (booster for Croquemort) in a transcriptomic analysis (RNA-seq) of Drosophila Schneider cells at different time points in efferocytosis followed by an RNAi-mediated approach. bfc is important for efferocytosis, the apoptotic clearance by macrophages, both in vitro and in vivo. The authors found that bfc in macrophages is required for efficient efferocytosis by increasing crq expression due to the interaction with Srp. Bfc physically interacts with srp and has been proposed as a new srp cofactor. The authors show that bfc interacts with the Srp zinc finger domain in a previously unknown manner and that both proteins work cooperatively to improve crq expression due to the binding of srp via a likewise newly identified GATA recognition sequence in the crq promoter.

My impression is that the publication meets the criteria for publication in PLOS Genetics. The work shows a high degree of originality in the area of genetic control of efferocytosis and is therefore undoubtedly of great importance to researchers in this and the fields of immunology and programmed cell death. In addition to the newly characterized gene Bfc/CG9129, which plays a role in transcriptional activation of Crq via Srp as a potential Srp cofactor, the authors have found further, partially uncharacterized genes for suppressing efferocytosis efficiency. I therefore expect widespread interest from genetic researchers in these disciplines, especially since only further transcription factors as srp cofactors have been identified so far.

The publication has compiled a very large amount of data that is adequately embedded in the literature and employing current methods to show consistent experimentation and evidence to support the conclusions. At some points in the manuscript I have pointed out what I believe are important points that need to be explained further or which could further clarify the results in further experimental approaches, which are all within the scope of the experiments that have already been carried out. The manuscript is clearly written, but in some places the linguistic expression should be improved and the use of abbreviations should always be mentioned the first time they are used. As far as I can tell, the authors have put together all the relevant data from their work in the online Supplements.

However, the large amount of data has also led to many hints, especially towards the end of the very extensive results section, being mixed up and, above all, the material and methods section is not being of the required quality, but this could certainly be compensated by extensive revisions, which are listed below.

RNAseq identified 48 genes with similar expression patterns to *crq*, which was upregulated after incubation with ACs.

- Please indicate why the threshold levels for crq expression are different from stated in M&M or correct this point in M&M.
- The Figure legend points to number of upregulated transcripts, for me it seems confusing at this place, maybe it is better to show them in the Results section.

Minor points:

- Please indicate also 1c.
- I am not sure if ”VS” is a general abbreviation for “versus”?

Bfc is involved in efferocytosis and regulates *crq* expression in S2 cells.

- Please indicate what the criteria to the highly similar expression trend to that of crq are?
- “We found the overexpression of CG9129 in S2 cells also up-regulated *crq* mRNA in the presence of ACs (Figure S2), and…” There is something wrong with this hint and not shown in Figure S2 or S2 is maybe incorrectly labeled. Please explain Figure S2a in more detail and indicate also S2b and correct Figure S2 and the complete Figure Legend and include the Crq-GFP construct and the overexpression in S2 cells in M&M.

Minor points:

- Please avoid abbreviations as in the chapter heading when Bfc is used for the first time same as crq in the previous chapter heading.

The expression of Crq is mediated by Bfc *in vivo.*

- Please correct the doubled sentences in the middle.
- Please indicate in M&M how many embryos are used for western blots.
- Please correct the heading for Supplement 3 with italics for expression and correct the heading for Figure S3 to all shown experiments. The expressions in S3a and S3b are reversed and please also state S3f in the figure legend.

Minor points:

- Include also S3e in the Results to S3d.
- And give an additional hint about S4 in the Results after mentioning simu for the first time.

***bfc* is required in macrophages for efficient efferocytosis.**

- Can you please add an additional quantification for Figure 3a.
- The authors introduce an additional MiMIC insertion as a strong LOF bfc allele and characterize it after generating and describing a newly generated bfc knock-out allele in the previous section of the Results. Please explain why you no longer use the KO allele or combine both descriptions more closely in the text.
- Acridin orange staining and efferocytosis phenotype: To me, the number of macrophages in bfc mutants appears to be reduced in 3e, f and also S5c! It would therefore be interesting to compare in Figure 3e also the bfc MiMIC allele and srp (due to the strong reduction of circulating hemocytes) in these acridin orange staining’s and possibly also to quantify the number of hemocytes in bfc mutant embryos. I think that can be an important point to show that the number of hemocytes is not strongly reduced in bfc mutants. In this context, adding of some information’s about both newly generated bfc mutants as viable or lethal would be helpful.

Minor points:

- Please add reference for PI.
- “To further determine if…” probably exchange to “confirm”.

- “reduced 35%” please indicate also 3i.
- Adapt the heading of the figure legend more to the content of the figure (include efferocytosis in S2 cells). Please indicate in all Figures with embryos or in M&M section the orientation of the Drosophila embryos with the dorsal side to the bottom as the agreed orientation is with the dorsal side to the top.
- S5: Please change Heading to ”bfc MI02020 mutants are defective for the efferocytosis of apoptotic cells” and correct spelling errors as “nuclear” and correct to Hoechst also in the Figure S5d.

Bfc physically interacts with GATA factor Srp.
- 1st sentence and later: Is there more information available on Bfc protein structure or domain organization?
- “Subcellular localization analysis revealed that Bfc-GFP fusion proteins predominantly localized in the nucleus of S2 cells (Figure 4), ~~and Ab staining showed that endogenous Bfc localized in the nucleus of S2 cells (Figure S5d)~~. Since Bfc regulated the transcriptional expression of *crq* and given its localization in the nucleus, we thus proposed that Bfc may act as a cofactor for the transcription factors of Crq.” Please indicate Figure 4d instead of 4 and again avoid abbreviations as “Ab staining”. But the stating is not shown in Figure S5d due to the overexpression situation in S5d. That means when overexpressing GFP::Bfc then you can´t detect endogenous Bfc or how can you differentiate endogenous from induced Bfc? But this is an important point to show! And probably the easiest way of doing is with the help of the Bfc antibody after induction of efferocytosis and with/without co-expressing mCherry::SRP!
- Please change “yeast two-hybrid screen” to “yeast two-hybrid interaction analysis” as no library was screened.
- Please explain the Results of Figure 4c and not only mention the Figure “We further confirmed the Bfc interaction with Srp in co-transfected S2 cells using Bfc-FLAG- and HA-Srp-tagged constructs (Figure 4c).” and please describe Co-IPs also in the M&M section.
- “Bfc co-localized with Srp” please indicate “tagged Bfc co-localized with tagged-Srp”.
- Figure 4a: Please correct the orientation of the Drosophila embryos with the anterior to the left as in all other embryo pictures. 4d: Correct also Bfc to GFP::Bfc and Srp to mCherry::Srp and Hochest to Hoechst.
- Include the generation of GFP::Bfc, Bfc::Flag and mCherry::Srp in the M&M section, possibly in a new subsection “S2 transformation vectors”.

Minor points:
- Figure Legend 4a: it is already stated in the M&M section in which animals the antibodies are made, please indicate here only in which color the 2nd antibodies were detected.

Bfc acts as a co-factor for the Srp transcription factor to regulate Crq expression and efferocytosis.
- Please indicate in the Results section, the Figure and Figure Legend the used srp allele.
- “The results showed Crq levels were decreased in the *bfc ko* and *srp* double heterozygous embryos (Figure 5a to c).” And also in srp or?
- “single heterozygous macrophages had no clear phenotypic difference from wild type”, please indicate “data not shown”.
- Please instead of “obtained … mutants” use “generated mutants … with Crispr/cas9 mutagenesis”.

Minor points:
- Please indicate the generation of crq ko also in the M&M section
- Please indicate in the M&M section by Statistical analysis what 4 **** mean in 5f and 5i?

Srp directly binds the *crq* promoter through GATA site recognition enhanced by Bfc.

- Please change S5a to S6a, S5b to S6b, S5e to S6e and indicate S6d when speaking about mutations in the ZnF domain and avoid abbreviations as "ZnF" before using the complete word for the first time.
- Please include the shown Co-IP results from S6c in the Results section.
- Immunofluorescence and WBs with FLAG-tagged proteins are shown in S7a and b, not in S6a and b.
- "…Immunofluorescent staining showed that the ectopic expression of Crq-Flag was much lower than that in the control…" An important point is how it is ensured that the newly generated and mutated crq::GAL4 construct shows the same expression as the Bloomington crq-GAL4 driver?
- Y1H assays: Please indicate "data not shown" for Bfc and Stat.
- "The Y1H results showed that only Srp-pADT7 interacted…" Please indicate Srp-AD as in the Figure.
- "Sequence analysis of the -1000 bp *crq* promoter ~~element~~ led to the discovery…" please change to " and further sequence analysis of the -1600 bp promotor led to the…" as shown in the Figure and indicate in the Figure 1.6 kb instead of 1.6k.
- "To confirm that the GATA sequence in the *crq* promoter is required for *crq* expression, we mutated the Srp binding site in the GATA sequence and integrated this *crqM*-Gal4 construct into the same genomic location to drive Crq-Flag expression in the wild type." I am confused by this sentences. What do you mean with "the same genomic location"? The same as what? I assume as crq-GAL4? I missed that in the M&M section? However, your M&M section describe the Bloomington crq-GAL4 driver and how did you know where the construct is located and how can you made sure if both driver lines produce the same strong signal (see above)?
- "Our results showed that *crqM*-Gal4 **failed to activate the transcription of *crq*** (Figure S7a and c)" Presumably because of the mutated GATA site, it cannot activate all other UAS constructs such as unspecific UAS-lacZ or UAS-GFP constructs, right?
- "Moreover, CHIP qPCR assays confirmed that Srp protein GATA sequence exhibits high specificity binding to the *crq* promoter (Figure 6c)…" What do you mean with that?
- The ChIP assay in Figure 6d: please indicate **n**! Is it usual to compare the whole input with the HA-beads with p-value? I know the way to show control and HA as percent of input and comparing control to HA with p-values.

Minor points:

- Why are the Figure Legends of S6 and S7 no longer in block set as all other Figure Legends?
- "In addition, the mutated Srp ZnF from Cys to Arg eliminated binding to the *crq* promoter (Figure S6f)." Please indicate also S6d in the Results section.
- Please correct Figure Legend heading S7: "The mutated GATA sites result to defective phagocytotic ability in Drosophila embryos." That is not shown in S7 since only expression patterns are traced!
- Please indicate also S7d-f in the Results section.
- Figure Legend 6: "black arrow head indicates…" Please correct the black arrow head to a "red arrow"
- "~~Positive and negative control were present. Yeast cultures were diluted (1:10 successive dilution series), spotted onto selective medium containing 300 ng/ml ABA and no Histidine (-His), then incubated for 3 d at 30°C~~." please put that information's to the M&M section.
- For the EMSA assay in M&M, please indicate which control exists, for what the S2 nucleoprotein (correct Nuceo to Nucleo) and the anti-HA were used?

Discussion

- "Here, we show a novel gene, bfc (Booster for Crq), that specifically regulates crq expression in a manner dependent the amount of apoptosis…" Please revise language.
- "Then, in vivo we found that developmentally programmed apoptosis, and during the first wave of apoptosis beginning at stage 11, when macrophage develop, crq expression is activated and subsequently becomes widespread throughout the 12 embryo." Dito.
- "We found *crq* had a curve expression trend in S2 cells incubated with ACs…" what does curved expression mean?
- Please indicate Figure 7 in the Discussion section.
- Please completely revise the language of Figure Legend 7 and change Acs to ACs.
- Figure Legend 7: "bfc is transcriptionally activated earlier than crq…". AND "We found that up-regulation of *bfc* expression occurred earlier than *crq* in S2 cells incubated with ACs…" Please refer to the corresponding Result illustration.
- In the paragraph on the role of srp and crq transcription I missed a reference to the already known srp cofactors and what distinguishes them from Bfc.

Material & Methods

Some parts of M&M section (e.g. "Apoptotic cells preparation…") is incomprehensible to non-familiar readers. "Observe efferocytosis und microscope (Zeiss)"? Please indicate microscope and used software etc. For RNAi treatment: "exact S2 cells DNA templates"? "Phagocytotic cells after RNAi treatment were collected for qPCR…" only these cells or all cells? The section needs a revision in the description of methodology and please use the same way for all units all over the section. Please indicate for the Western Blots on embryos how many embryos you used for protein extracts? Please revise the AO staining. Please indicate the reference for phagocytic index.

Please include the production of the bfc antibody to the M&M section, also the antibody staining on S2 cell cultures and Drosophila embryos and include all used antibodies (Flag, mCherry, etc.). Describe Co-IPs and include the generation of all constructs (e.g. UAS-crqFLAG, GFP::Bfc, Bfc::Flag and mCherry::Srp)!

For what purpose you need the bfc sgRNA? Please refer in the Result section.

Check whether the order of methods can be improved.

English

Correct several spelling errors all over the manuscript and several expressions, some examples are stated here:

Graph shows the ratio of twenty-four gene mRNA expression in each gene RNAi treated S2 cells when compared to control-treated control S2 cells, as determined by qPCR…

Which consistent with previous study…

Moreover, CHIP qPCR assays confirmed that Srp protein GATA sequence exhibits high specificity binding to the crq promoter…
