# Supplementary material for: bfc, a novel serpent co-factor for the expression of croquemort, regulates efferocytosis in Drosophila melanogaster
Source: PLoS Genet. 2021 Dec 3;17(12):e1009947. doi: 10.1371/journal.pgen.1009947 (PMC8673676; doi:10.1371/journal.pgen.1009947)
Supplement: Attachment — Submitted filename: Reviewer 2 comments_Zheng et al_revision1.pdf [file pgen.1009947.s019.pdf]

The authors have largely satisfied my queries and the manuscript is much improved. Some minor points remain to be resolved before the manuscript can be accepted in my opinion but these should be easy to address. I hope the authors have found my comments constructive and helpful; I have enjoyed reading this paper. My comments follow the lead author's response to my original comments and are prefixed by "Reviewer:"

Major points

1.I am afraid that I found the manuscript hard to follow in many places. The figure legends and methods are insufficiently detailed and in many places the results are not described well enough to follow what has been done. The methods are written in a note form that is closer to a protocol than the methods section of a publication. Therefore, the entire manuscript would benefit from careful rewriting to ensure sufficient detail is included to aid the reader. This is particularly the case in the section relating to Figure 6 and 7. Additionally, in some places the language used is not grammatically correct or could be improved to help understanding.

RESPONSES: We thank the reviewer for this very insightful suggestion. We have rephrased the figure legends and methods with sufficient detail and rewrite the results in the revised manuscript, particularly in the section relating to Figure 6 and 7. We also thank the reviewer very much for reminding us of these grammar flaws in our original manuscript. We have examined the text more carefully and corrected the erroneous sentences as well as other spelling grammar mistakes.

Reviewer: The manuscript is much improved but some sections still need attention and very careful copy editing. For example, several sections of the methods remain in note form as per a protocol (e.g. AO, ChIP and Y1H sections). There are quite a few typos or sentences in which words are missing making it hard to follow in places.

2. The statistics section in the methods is too brief and it appears that inappropriate statistical analyses have been used in many of the figures: according to the methods section it seems that multiple t-tests have been used to compare >2 experimental conditions. Details of the statistical tests used are not described in the figure legends. Therefore, all numerical data and the statistical tests used to compare experimental groups need careful checking and (if needed) a different statistical analysis performing. This may change whether certain data remains statistically significant, so conclusions may need to be modified.

RESPONSES: We thank the reviewer for this very good suggestion. We have rephrased the "Statistical analysis" part of M&M section with more detail and described it in the figure legends. We have carefully checked all numerical data and the statistical tests used to compare experimental groups. We used t-test to compare the means of two groups. For the statistics of those results which have more than two experimental samples to be compared were derived using ANOVA in revised manuscript.

Reviewer: If using ANOVA's please state the post-test used in the Figure legends. Otherwise, point 2 has been addressed. Should Fig 2E not also be a 2-way ANOVA rather than a 1-way ANOVA? Maybe this is a mistake in the legend? Fig 3C it is probably ok to use a t-test rather than an ANOVA (as written in the legend at least).

3. While I am largely convinced that Srp and Bfc interact and that Srp is binding the crq promoter via a GATA site, I am not convinced that the mechanism by which Bfc is working

with Srp is correct or supported by the data presented. This model and Figure 7 are not explained very clearly in the manuscript. I am not sure the data supports the idea that Bfc increases the amount of Srp bound to the crq promoter. However, the way in which the IP and EMSA data are described does not help the reader. Can the authors show that the presence or absence of Bfc regulate the interaction of Srp with the crq promoter (e.g. via ChIP)? For instance: P11 "Bfc interacts with the zinc-finger domain of transcription factor Srp as a co-factor to enhance Srp binding to the crq promoter to elevate its expression and induce efferocytosis in Drosophila melanogaster." I am not sure that the EMSA data is consistent with this conclusion, but it is not adequately described in the manuscript.

Aditionally, it is not clear what the controls on the left of Fig 6d are or what the panel on the furthest right is demonstrating, as this is not explained anywhere. Authors claim an increase in the migration efficiency of the Srp-DNA complex in the presence of Bfc. Is this the very faint band that can just about be made out below the main band? If so, it is not clear what this means in terms of the mechanism and does not appear to be consistent with Bfc binding to the DNA in concert with Srp, as that should decrease mobility of the band. It is thus not clear how the interaction of Bfc with Srp is functioning to increase Crq levels from this data. Also, why is the Bfc + Srp condition repeated so many times on this gel?

On page 14: "Furthermore, Bfc-Srp interaction occurred through the ZnF GATA domain and that the presence of Bfc increased the ability of Srp to bind the crq promoter." I don't think this is substantiated by the data – if it refers to the slight increase in intensity in Fig 6d (not quantified) and this seems an inappropriate conclusion as there is not an excess of DNA (no free probe visible at the bottom). Therefore, probe is rate limiting for complex formation and so adding a fraction more in one lane would change intensity.

RESPONSES: We totally agree with the reviewer that the way in which the IP and EMSA data are described does not help the reader. We have reperformed the ChIP experiment and showed the input percent used in ChIP as well as added the blank S2 cells without transfection of HA-Srp as a negative control (please see Figure 6). Moreover, we have added the luciferase assays to prove that bfc knock-down resulted in a decreased activity of crq promoter in HA-Srp transfected S2 cells (please see Figure 6E-F). In addition, as instructed by the reviewer, we reperformed the EMSA experiment and found the addition of purified Bfc protein to purified Srp-DNA complex resulted in a super-shift band when compared to Srp-DNA shift band, and the more Bfc were added, the more super-shift band can be observed. (please see Figure 6 and page 16, lines 442-445). We described the method in more detail in M&M (please see pages 28-29, line 774-800).

Reviewer: The new ChIP data with additional controls etc. supports the conclusions more effectively and shows a requirement for Bfc in the effects of Srp-HA overexpression on crq promoter activity. The new data more fully supports the original conclusions but the paragraph describing this part of the project could still be written more clearly. If there is an issue with the word count, I suggest the editors relax word count restrictions or other sections are abridged to enable a fuller, more clear description of these important experiments. There are several typos in this section. E.g., line 439 used > using; missing "extract" after nuclear from this line? Line 440 missing "DNA/promoter" after crq? Figure 6E' described before 6E in the text – consider reordering the text or the figure.

4. Authors claim to have analysed STAT92E, but no data is presented: "To detect the physical interactions between regulatory transcription factor proteins and crq in the genome, we then performed yeast one-hybrid (Y1H) assays (Reece-Hoyes and Marian Walhout, 2012) between Bfc, Srp, Stat92E, and the crq promoter locus, respectively."

RESPONSES: We thank the reviewer for this very good suggestion. We have added this data in the revised manuscript (please see Figure S9).

Reviewer: Thank you for including this data.

5.Apoptotic cell clearance is only analysed in the head. Can defects in apoptotic cell clearance be detected elsewhere in the embryo in bfc mutants? Dcp-1/Crq staining is only shown for the head region. Acridine orange staining looks like it may be less affected in the absence of Bfc along the ventral regions of the embryo. This is particularly important due to the emerging idea that different subtypes of macrophage may be present in the fly (e.g., Cattenoz et al., 2020; Tattikota et al., 2020).

RESPONSES: We apologize for not providing a clearer description of our apoptotic cell clearance assay, which was modified from the previous study (Silva E et al., 2007, Immunity 27:585-96). We agree with the reviewer that the amount of engulfment also depends on the location of the macrophages in the stage 13 embryo. Thus, we ensure that measurements are taken from at least five different embryos of any given genotype, and also ensure that we have taken images of macrophages in all three regions (head, tail, and CNS) for calculating the phagocytic index. We have added the details of how we calculated the PI in Drosophila embryos in M&M section (please see page 27, lines 729-736).

Reviewer: Authors should confirm with editor that the methods describe the original analysis of PI rather than a new analysis since the graphs have not changed between submissions. Otherwise I am satisfied with this.

6.Is anti-Crq the best marker to stain and visualise macrophages accurately in bfc mutants if Crq protein levels are so decreased in the absence of Bfc (Figure S5c and other similar figures)? I have always found it difficult to see how phagocytic index is accurately measured using anti-Crq staining in comparison to, for example, use of srpHemo-GAL4, UAS-GFP and other such tools; it is hard to see where one cell begins / ends and how many cells are present in these images. It is also hard to see what is going on due to the overlap of red and green staining in these images (can single channel images be displayed with the merge?) Also, please consider using green/magenta rather than green/red due to those suffering from colourblindness, especially if separate channels are not to be displayed. What do the white dotted circles represent? Individual cells? Lastly, the use of anti-Crq makes it difficult to score these images blindly as mutant and wt/rescues can easily be discriminated and this appears to be quite subjective scoring anyway (precise details are missing from the methods). N.b., these comments apply to all figures that show anti-Crq/anti-Dcp-1 staining.

RESPONSES: We thank the reviewer for this very insightful suggestion. We performed our apoptotic cell clearance assay, which was modified from the previous study (Silva E et al., 2007, Immunity 27:585-96). We took confocal Z-stacks of embryos that have been labeled for apoptotic corpses by use of anti-Dcp-1 staining and for macrophages by use of anti-Crq staining. By taking a stack of images through a labeled macrophage, it is possible to count the number of Dcp-1–stained corpses inside one macrophage. The white dotted circles

represented individual macrophage. As suggested by the reviewer, we used green/magenta rather than green/red. We agree with the reviewer that by using of srp-GAL4, UAS-GFP, it is easy to see where one cell begins and ends. Following the reviewer's suggestion, we used the fly strain srp- gal4>UAS-GFP(BDSC#78565) to mark macrophages and stained corpses by anti- Dcp1.We found the apoptotic cell clearance phenotype such as phagocytic index (PI) was quite similar to our anti-Crq/anti-Dcp-1 staining assay (please see S5 Figure).

Reviewer: This is reassuring. Thank you for this and apologies for being a bit pedantic.

7.Figure 4d shows Bfc is nuclear in S2 cells. Can a nuclear marker/dye and higher magnification imaging show a similar localisation in macrophages in the embryo? What happens to Srp localisation on RNAi of Bfc from S2 cells? This should be fairly easy to do with the reagents already in hand and may give an insight into how the Bfc-Srp interaction works to regulate Crq levels (especially as I am not sure about the EMSA data).

RESPONSES: We agree with the reviewer. We used Hoechst 33342 to label the nuclear together with anti-Bfc and anti-Crq in vivo. We can confirm that Bfc is localized in macrophages but not the subcellular localization of Bfc which may be due to the quality of our Bfc antibody or the limited resolution of laser confocal. We have isolated the macrophages from larva and stained them with anti-Bfc to confirm that Bfc is localized in the nuclear (please see Figure 4B"). Moreover, we performed bfc RNAi in S2 cells, but the localization of Srp seemed no different when compared to control (please see Fig 4E-E').

Reviewer: The anti-Bfc staining in B'' is not hugely convincing and the Bfc-GFP localisation in S2 cells is rather odd. Can the authors show a *Bfc*^{KO} mutant macrophage as a negative control and/or controls corresponding to pre-immune sera or a no primary antibody control?

8. Why are immune genes like cecropin coming up in the transcriptional profiling? This seems counterintuitive to the silent clearance of apoptotic cells, as it suggests immune activation. We ourselves have had issues generating apopototic cell samples via similar methods – we obtain apoptotic cells but also a large amount of necrotic cells. The AC samples used/method therefore require characterisation/validation (e.g., caspase activity probes, annexin, PI – all widely available). It therefore is possible that some of the genes that are being upregulated are in response to necrotic cells present in the samples in addition to the apoptotic ones. The AC samples should be characterised and if necrotic cells are present the conclusions need to be modified to acknowledge that the transcriptional changes observed may not be specific to contact with apoptotic cells.

RESPONSES: We thank the reviewer for this very good suggestion. As the anti-inflammatory nature of the apoptotic process, factors released from apoptotic cells should suppress inflammation. But there were some references that also showed that some signals from ACs activated the inflammation of macrophages. We added this in the discussion part (please see page 18, line 493-499). As instructed by the reviewer, we validated the ACs samples added to live S2 cells and showed that most of them is apoptotic cells rather than necrotic cells (please see S1A-S1C Figure). However, we really cannot totally eliminate the up-regulated genes that may be induced by bits of necrotic cells, so we modified the conclusions to acknowledge that the transcriptional changes observed may not be specific to contact with apoptotic cells (please see page 18, line 485-490).

Reviewer: I am satisfied with these changes. The Although at the start of the sentence on line 485 can be deleted. Legend should state which %/quadrant relates to AC.

9. In general, the discussion needs to discuss the findings in a broader context, as it mostly simply repeats the conclusions/findings of this paper. In particular there is barely any discussion of the work in relation to other organisms (only 1 mammalian paper is cited). For instance, do homologs of Bfc exist in vertebrates? Is this a fly specific mechanism? A careful re-write of this section will make it much more appealing and relevant for non-Drosophilists.

RESPONSES: We appreciate the reviewer for pointing out our discussion needs to discuss the findings in a broader context and apologize for our neglect of citing and discussing the work in relation to other organisms. We have now provided a discussion on these points and rephrased the discussion part in the revised manuscript.

Reviewer: This is much improved.

Minor points

1. Figure 1d-e what does % phagocytosis mean? I presume % of S2 cells phagocytosing AC – please make clearer in legend and methods.

RESPONSES: We apologize for not providing a clearer description of our apoptotic cell clearance assay. We made it clearer in legend and added the detailed method in M&M section (please see page 24, lines 653-665).

Reviewer: This is still not adequately explained. Is it the % of cells phagocytosing/efferocytosing AC? Please state this in the legend.

2.I am not sure that it is possible to make the conclusion that "as an engulfment receptor, Crq contributed to early recognition of ACs, while engulfment at later stages likely depended on other regulatory factors to mediate further AC clearance." It is not clear whether the internalised AC were internalised at earlier times and have not yet been degraded or have been internalised more recently. In my opinion to test this properly you would need to stimulate with AC, remove excess AC and then challenge again, or something similar (if this was actually how the experiment was carried out it is not clear from the results, methods or figure legend). I don't propose this experiment is repeated as suggested, as this doesn't seem a key point, just that the conclusions are modified.

RESPONSES: We totally agree with the reviewer that it is not clear whether the internalized AC were internalized at earlier times and have not yet been degraded or have been internalized more recently based on our experiment. We modified the conclusion (please see page 4-5, line 108-110, line 121-126).

Reviewer: This has been addressed.

3.What are the experimental conditions for Figure 2c,d? This is not clear from the text, methods or legend. I presume this is with AC treatment, but for how long etc?

RESPONSES: We apologize for not providing a clearer description of experimental conditions for Figure 2c,d. We modified the Figure legend to make it more clearly (please see page 7, line 188-194).

Reviewer: Addressed but please carefully proof the legend(s) as there are some missing words that make it hard to read, e.g., "adding ACs 6h" -> probably should read "adding ACs for 6h".

4.The effect of knockdown of CG9129/bfc is far stronger than for crq itself (Figure 2c), what other processes may be affected given bfc seems to play no role for SIMU and drpr? It may be nice to speculate on this in the discussion.

RESPONSES: We thank the reviewer for this very good suggestion. The effect of knockdown of CG9129/bfc is far stronger than for crq itself, which may be caused by the different RNAi knockdown efficiency of bfc and crq. We totally agree with the reviewer that although there were no effects on drpr and simu expression in bfc^{ko} mutants, other processes may be affected by bfc, such as the clearance of neuronal debris and bacterial clearance. We speculate on this in the discussion part.

Reviewer: Now included.

5.Figure 3a appears after Figure 3c-d. Authors may want to reorder the text / figure slightly to improve how the manuscript reads. There are quite a few panels not cited (e.g. Fig S3e) in the text or mistakes in which figures are being cited throughout the manuscript.

RESPONSES: As instructed by the reviewer, we have reordered Figure 3 in the text (please see page 8, line 205-208) and corrected the mistakes in citing the figures.

Reviewer: Addressed.

6.Not all of the inset can be seen in the main field of view for the central panel of Figure 3a, while the zooms could have been a bit bigger. I also struggled to see where the regions were taken from (in fact I am unable to see where the zoom for bfc RNAi came from at all for that field of view).

RESPONSES: This is a very good suggestion. We have shown the magnified regions by the white dotted squares in Figure 3A.

Reviewer: The white box for bfc RNAi is not quite in the right place and the brightness of the zoomed regions is different from the main panel. Authors may want to address this to be consistent/make their manuscript as robust as possible.

7.Please provide details of how the mouse anti-Bfc was generated (what epitope was used etc). Authors should check for other methods that are not described in the paper.

RESPONSES: As instructed by the reviewer, we have provided details of how the mouse anti-Bfc was generated to the M&M section (please see pages 25-26, lines 691- 700). We also rephrased our M&M section.

Reviewer: Thank you.

8. Figure 4a - why is the green channel so bright in the bfc[ko] embryo? Have these embryos been imaged using the same settings/enhanced similarly? Why is the green channel so faint in the boxed region of the bfc[ko] panel compared to the main image? Individual channels should be shown and advisable to use magenta in place of red for merges. Also, it would be better not to inset zoomed regions over the posterior of the embryo as this prevents the reader from analysing expression in posterior macrophages.

RESPONSES: We apologize for not providing a clearer description of experimental conditions for Figure 4a in the original manuscript. Because we used the intensify exposure of Crq staining to show the macrophages in the bfc^{ko} embryo. We have imaged WT and bfc^{ko} embryos by the same settings and used magenta in place of red to show individual channels as well as merges. As instructed by the reviewer, we put the zoomed regions in the upper right corner of each embryo to avoid covering the posterior macrophages (please see Figure 4A).

Reviewer: Thank you.

9.References required for this statement on p8: "Previous researches have demonstrated that several signal pathways regulated the expression of engulfment receptor Drpr, such as d-JNK, PI3K and insulin."

RESPONSES: We thank the reviewer for reminding us of this point. We have added the corresponding references in the revised manuscript (please see page 12, lines 320- 321).

Reviewer: Thank you.

10. page 10 – some confusion / mix ups for figure labelling for Figures S5-7. Not all panels are cited in the text.

RESPONSES: We apologize for these mistakes. We have corrected the text in page 10 for S5 Figures (now it's S7 Figure, please see page 14-15).

Reviewer: Thank you.

11. Why does bfc not work to regulate the amounts of other Srp-dependent receptor genes? Authors suggest other co-factors regulate Srp's activity at the drpr and simu promoters but I don't think their model is sufficient to explain why Bfc would not assist at those promoters given their regulation by Srp. Perhaps it would be clearer with more explanation?

RESPONSES: This is another very good point. The transcriptional factor Serpent (Srp) was found to be required for sufficient phagocytosis of ACs in Drosophila embryonic macrophages by regulating SIMU, Drpr and Crq (Shlyakhover E et al., 2018, Front Immunol 9:266). However, there is no evidence that Srp directly regulated the expression of SIMU, Drpr and Crq. Moreover, we did not detect the binding of Srp to drpr promoter (Please see S9 Figure). Another explanation is that Bfc would assist Srp at promoters of simu and drpr, but have very minor effects, thus we could not detect the effect in bfc mutant by our current methods. We added more explanation in the discussion part (please see page 19, lines 521-525).

Reviewer: Addressed.

12.P13 “These results imply that the expression of phagocytotic receptors could be stimulated by the presence of excessive ACs to improve the phagocytotic activity of macrophages.” This has been shown by a number of other labs in the fly (e.g., Wood and McCall) in flies and is well-documented in vertebrates. These and other papers should be cited and this new mechanism contrasted with how this is regulated in other systems.

RESPONSES: We appreciate the reviewer for pointing out our carefulness and apologize for our neglect of citing the findings of other labs in flies and in vertebrates. We have now correctly cited their works and discussed their mechanism in our revised manuscript (please see page 19, line 508-514).

Reviewer: Addressed.

13. Authors may wish to include Han et al. (2014) and Guillou et al. (2016) in the discussion since these suggest functions for Crq other than a role as a receptor for apoptotic cells.

RESPONSES: We thank the reviewer for reminding us of this point. we have now correctly cited Han and Guillou works in our revised manuscript (please see page 20, line 554).

Reviewer: Thank you.

14. Supplemental tables 3 and 4 are not cited in the manuscript.
RESPONSES: We apologize for this mistake. We have now correctly cited S3 Table and S4 Table in the M&M section (please see page 25, line 671 and page 26, line 696).

Reviewer: Thank you.
