# Supplementary material for: bfc, a novel serpent co-factor for the expression of croquemort, regulates efferocytosis in Drosophila melanogaster
Source: PLoS Genet. 2021 Dec 3;17(12):e1009947. doi: 10.1371/journal.pgen.1009947 (PMC8673676; doi:10.1371/journal.pgen.1009947)
Supplement: Attachment — Submitted filename: Zheng et al. Bfc Review 2.pdf [file pgen.1009947.s020.pdf]

Bfc, a novel _Serpent_ co-factor for the expression of _Croquemort_, regulates efferocytosis in _Drosophila melanogaster_

Thank you very much for providing me with a lined manuscript!

I see the authors have met the main criticisms, concerns and suggestions of the first review. At some points in the revised manuscript, however, further explanations have to be provided in order to make the results clearer. The manuscript is now clearly written, but the linguistic expression can be further improved and please check again the use of abbreviations especially in headings. This can certainly be compensated by some minor revisions that are listed below.

Author summary
Line 32: ". such…"

Introduction
Line 74: "Drosophila; …"

RNAseq identified 48 genes with similar expression patterns to _crq_, which was upregulated after incubation with ACs.
Line 102-103: *crq* and ACs!
Line 153: The numbers in the Venn diagram differ from the information in the Figure Legend. In addition, there was a correction that should be shown on lines 141-142, but this line only shows the title of the figure legend.
Line 141: "The ~~significantly~~ upregulated…"

Bfc is involved in efferocytosis and regulates _crq_ expression in S2 cells.
Line 184: Please avoid abbreviations as in the chapter heading when Bfc is used for the first time, the same as crq in the previous chapter heading.

The expression of Crq is mediated by Bfc _in vivo._
Line 253-254: Please rephrase or explain.
Line 256: …blot analysis of Crq protein levels… please add also Bfc!
Please correct the heading for Supplement 3.

bfc is required in macrophages for efficient efferocytosis.
Line 282: Please explain the Result of the newly designed Figure S5 and not only mention it.
Line 299: ~~meanwhile~~
The hint to Figure 3J is missing!

Bfc physically interacts with GATA factor Srp.
Line 304: Bfc ~~is~~ expressed…
Line 307: "premised it was a transcription factor" please rephrase "premised it encodes a transcription factor".
Line 312: "…examine the ~~expression location~~ of Bfc…" please rephrase "protein localization".
Line 317: Fig 4B´-B´´ please change because B´ and B´´ are not similar as B! The same for E and E´ in Fig. 4.
Line 345: Please insert "as well as" after control S2 cells!

Bfc acts as a co-factor for the Srp transcription factor to regulate Crq expression and efferocytosis.

Srp directly binds the *crq* promoter through GATA site recognition enhanced by Bfc.
Line 403-404: …that ~~**only**~~ Srp-AD interacted **only** with the crq…
Line 408-409: …mutated Srp Znf from Cys to Arg eliminated binding to the *crq* promoter (S7D and S7F Fig). This is not shown in S7f due to only binding of Srp-AD to the *crq* promoter!
Line 412-414: Our results showed that the ability of *crqM*-Gal4 **to activate the transcription of *crq*** was much weaker than *crq-GAL4*"
This statement sounds to me like the failure of a specific transcriptional activation of *crq* due to the mutated GATA site. But due to the mutated GATA site, all other UAS constructs such as unspecific *UAS-lacZ* or *UAS-GFP* constructs cannot be activated either, right?
Line 414: …in *bfc* also has an insufficient activation to *crq*… Please rephrase to …in *bfc* also leads to insufficient activation of *crq*…
Line 421: Please correct the red arrow in Fig. 6A to the indicated red arrowhead or vice versa.
Line 430: E´ please change because E´ is not the same as E!
Line 439-441: HA-Srp… Can you explain or please rephrase?
Line 445-447: Our results showed… Can you explain the results of Figure 6F in more detail or please rephrase?

Discussion
Line 528: Meanwhile?

Material & Methods
Line 776- 778: This technique… avoid such sentence please.

Supplemental Information
Line 1125: S1 Fig. ACs were ~~successfully~~ induced… Please check for abbreviations used for the first time!
Line 1128-1129: …~~as described in the method~~.
Line 1174-1175: S4 Fig. Title? Please indicate used arrowheads and indicate all micrometer steps. Please explain the newly designed Figure in the Results section and not only as a hint to S5.
Line 1206-1207: ~~Yeast two-hybrid shows the interaction between Bfc and C4 M of Srp~~.
Line 1209: …interaction between C4 M of Srp and crq promoter. Please indicate „C4 M of Srp" in S7F.
Line 1211: Please correct the heading of S7 to: "…of *UAS-Crq* expression in *Drosophila* embryos." Since only expression patterns of effector lines are traced!

English
Please check again the linguistic expression all over the manuscript.
