# Supplementary material for: bfc, a novel serpent co-factor for the expression of croquemort, regulates efferocytosis in Drosophila melanogaster
Source: PLoS Genet. 2021 Dec 3;17(12):e1009947. doi: 10.1371/journal.pgen.1009947 (PMC8673676; doi:10.1371/journal.pgen.1009947)
Supplement: Attachment — Submitted filename: Zheng et al. Bfc Review 3.pdf [file pgen.1009947.s022.pdf]

***Bfc*, a novel *Serpent* co-factor for the expression of *Croquemort*, regulates efferocytosis in *Drosophila melanogaster***

I see the authors have met the main criticisms, concerns and suggestions of the first two reviews. The manuscript is now clearly written, but please check again the use of abbreviations especially in headings. In some places in the revised manuscript, however, further explanations are required to clarify the results. In addition, please check again the order in the material and methods section and the use of formal genetic nomenclature in the manuscript (except for the discussion) needs to be re-examined. This can certainly be compensated by some revisions that are listed below.

**Title page**
Line 1 and 12: ~~*Bfc*~~=*bfc*, ~~*Serpent*~~=*serpent* ~~*Croquemort*~~=*croquemort*, ~~*Bfc*~~=*bfc*
Possibly because of the title page, but formally incorrect.

**Abstract**
Line 16: ~~in other words~~
Line 21: ~~*Bfc* (booster for croquemort)~~ = *bfc* (*booster for croquemort*)!
Here the novel gene is described. Please always use lowercase and italic notation, while uppercase and non-italic notation have to be used for the corresponding protein

**Key words**
Line 31-32: Alphabetical order?

**Authors summary**
Line 40: **L**upus
Line 42-43: Sentence construction?
Line 44-45: gene: *bfc* ~~(Booster for Croquemort)~~ = *bfc* (*booster for croquemort*)

**Introduction**
Line 56: **whereby** apoptotic cells…
Line 80: …**s**eemed…
Line 88: the transcription~~**al**~~ factor…
Line 95: gene: *bfc* (*booster for croquemort*)

**Results**
**RNAseq discloses 48 genes with expression patterns comparable to that of *crq* in S2 cells co-incubated with ACs**
Line 106: ~~Here~~
Line 118: above mentioned…
Line 146: AC **e**fferocytosis…

***CG9129* is involved in efferocytosis and regulates *crq* expression in S2 cells**
Line 184: Protein: Booster for crq (Bfc)

***crq* expression is regulated by Bfc *in vivo***
Line 210: Crq (mRNA or protein?) for me it seems *crq* expression?
Line 223: (~~Figure S3E~~) allover the manuscript it is (S3E Fig) and also include (S3D-S3E Fig)
Line 229: Please explain use of *H99* embryos as in the corresponding Figure legend

Line 231: …embryo**nic** development…
Line 232: …Crq expression… mRNA or protein?
Line 237: …expression of *drpr* and *simu*… Data not shown for *simu*?
Line 243-246: ~~Moreover~~, we did not observe… Why moreover? And please explain the following part in more detail for better understanding!
Line 261: Please change UAS-Bfc to *UAS-bfc*
Line 264: stage 13 embryos ~~on the ventral view~~. As far as I can see, it is a lateral view
Line 266-267: These embryos… Please clarify!
Line 270: Please change UAS-Bfc to *UAS-bfc*

M**acrophages require Bfc for efficient efferocytosis**
Line 294-295: …both *bfc*…, while *bfc*…? Please explain
Line 300, 301, 303: Bfc: all *bfc*?
Line 308: …we generated a mutant with a MiMIC insertion… But you write in the M&M section that you got the mutant from the Bloomington stock keeping center. Please clarify!

***CG9129* physically interacts with GATA factor *Srp***
Line 322: Italics for *CG9129* and *Srp* in the context of physical interaction?
Line 330-331: …revealed ~~a strong~~ Bfc ~~expression~~ protein localization in macrophages…
Line 350-351: Title of the figure legend seems not appropiate to me due to protein extracted from S2 cells and not from embryonic hemocytes as stated in the heading

***CG9129* and Srp act together to regulate Crq expression in context of efferocytosis**
**Heading:** Spelling of *CG9129,* Srp and Crq expression? …**the** context…?
Line 371: …*bfc*/*srp* ~~**mutant**~~ **double** heterozygous embryos
Line 374: …exhibited similar defects… Please also explain the defects!
Line 382: *crq*/*bfc* **double** heterozygous mutants
Line 384: …and phagocytic defects as those observed in… Please explain the defects!
Line 388: Spelling of *CG9129*?

***CG9129* enhances direct binding of GATA factor Srp to the *crq* promoter**
**Heading:** Spelling of *CG9129* and Srp?
Line 430: …we mutated the Srp-binding site in the GATA sequence and integrated this construct, or the *crqM-Gal4* construct as a control…? Maybe: we mutated the GATA sequence in the Srp-binding site and integrated this *crqM-Gal4*…
Line 435: Meanwhile?
Line 435-438: Can you please rephrase for better understanding
Line 438-444: Please also refer to the corresponding Figures
Line 432-437: Please refer also S8F
Line 471: …*bfc* ~~**and**~~ **or** *srp*…
Line 477: …Srp protein~~**s**~~…
Line 479: …labeled crq… mRNA or protein?
Line 486-491: Please refer to corresponding subfigures S10 A-D not only S10

**Discussion**
Line 558: Please change CO-IP to Co-IP
Line 573: As a **potential** Srp cofactor…
Line 579- 582: Also… Please rephrase sentence for better understanding

**Material & Methods**
Line 640: Please change vasa Cas9 to *vasa Cas9*
Line 643, 644,648: Change *gal4* to G*al4*
Line 655: Please change UAS-Bfc/UAS-CrqFlag to *UAS-bfc*/*UAS-crqFlag*
Line 709: Please change …~~away from the light~~ to …in the dark
Line 735: …according ~~to the report of~~…

**Figures**
Figure 4: Please indicate in B´-B´´´ S2/S2/Mp/Mp for better understanding

**Supplemental Figures and Information**
Line 1080: Please change …~~annexin~~ to …Annexin V-FITC
Line 1081: Bar~~**s**~~ and … ~~**a**~~**A**ctinomycin D
Line 1100: Figure title indicates expression *in vivo* but G and H display mRNA levels in S2 cells
Line 1121: Please rephrase Figure title
**S4C** Please indicate bars 3+4
Line 1130: Please rephrase Figure title and change UAS-GFP to *UAS-GFP*
Line 1140-1141: The *crq* and *bfc* mRNA levels were quantified by qPCR using wild-type and *bfcMI02020* cells. Can you please explain what does cells mean?
Line 1144, Line 1147: Please change UAS-Bfc and UAS-Crq to italics
Line 1149: Please change **E to F**
Line 1151-1152: ~~The white arrowheads point to~~... There is no arrowhead in F
**S9D** Please correct Srq to Srp
