# Supplementary material for: bfc, a novel serpent co-factor for the expression of croquemort, regulates efferocytosis in Drosophila melanogaster
Source: PLoS Genet. 2021 Dec 3;17(12):e1009947. doi: 10.1371/journal.pgen.1009947 (PMC8673676; doi:10.1371/journal.pgen.1009947)
Supplement: Attachment — Submitted filename: Zheng et al. bfc Review 4.pdf [file pgen.1009947.s024.pdf]

***bfc*, a novel *serpent* co-factor for the expression of *croquemort*, regulates efferocytosis in *Drosophila melanogaster***

The authors have taken into account all of the criticisms, concerns and suggestions made in the reviews. The manuscript is now clearly written and, in my opinion, has been significantly improved.
