# Supplementary material for: bfc, a novel serpent co-factor for the expression of croquemort, regulates efferocytosis in Drosophila melanogaster
Source: PLoS Genet. 2021 Dec 3;17(12):e1009947. doi: 10.1371/journal.pgen.1009947 (PMC8673676; doi:10.1371/journal.pgen.1009947)
Supplement: Attachment — Submitted filename: bfc response to reviewer 6.10.docx [file pgen.1009947.s018.docx]

**Listed below are our point-by-point responses to the reviewer’s comments.**

**Reviewer #1**

**Summary**

In their manuscript, Zheng et al. identify CG9129/Bfc as a gene required for proper expression of the apoptotic receptor Croquemort in Drosophila S2 cells and embryonic macrophages. Consistent with this observation, they found that Bfc loss is associated with a decrease in apoptotic cell phagocytosis. They further show that Bcf interacts with the GATA transcription factor Srp and their results suggest that Bfc could promote the direct binding of Srp to crq, thereby favoring crq transcription.

These findings are of potential interest but several conclusions are poorly substantiated. Importantly too, key controls and information are missing. Moreover, the level of analysis is rather limited and, in my opinion, beyond the expectations for PLoS Genetics.
Finally, the manuscript is poorly presented: several pieces of results are not well described or presented in a logical order, the figure calling is not accurate and the figure layout is often difficult to grasp, the text is plagued with typos and grammatical errors, and the “methods and materials” section is not appropriately written.

The comments below may help the authors to improve their manuscript.

**Major points**

1.The authors should mention how many replicates were used for the RNAseq. Page 4: it does not make any sense to write that ±9000 genes (i.e. ±70% of the genes encoded by the genome) are differentially expressed at the different time points; differential expression must be defined with clear cutoff (fold change and p values, as used page 5).

**Responses**: We thank the reviewer for this very insightful suggestion. We performed one transcriptomic analysis of S2 cells engulfing ACs, so we used quantitative real time RT-PCR (qPCR) in triplicate experiments independently to validate the RNA-seq data. We have defined differential genes expression with a clear cutoff. (**please see pages 5, line 130-134**).

2. Page 5: “engulfing S2 cells displayed changes in multiple transcriptional programs”: where are the analyses /tables for such conclusion?

**Responses**: We apologize for this mistake. We have added the related figure to support this conclusion (**please see Page 5-6, line 137-139, Figure 1F**).

3. Page 5 / Figure 2: the criteria used to select the 24 genes are not very clear; some genes that were kept/eliminated do not seem to strictly follow the threshold/statistical significance criteria (eg: PGRP-SA, CG12112…).

**Responses**: We thank the reviewer for this very good suggestion. The criteria used to select the 50 genes for qPCR is presented in **S1 Table**, and the criteria used to select the 24 genes is presented in **Figure 2** according to the significant difference in the revised manuscript. Genes were kept or eliminated according to the qPCR results.

4. How the author explain that several genes tested by RT-qPCR did not behave as in the RNAseq experiment ?

**Responses**: We apologize for not providing a clearer description of our RNAseq and RT-qPCR experiment. The RNAseq samples were collected from one experiment, and RT-PCR (qPCR) were performed in triplicate experiments independently to validate the RNA-seq data.

5. Page 5 / Fig. 2c: it is not clear how efferocytosis efficiency is measured. Also the effect of crq knock-down seems very mild and it is thus questionable whether this assay is a good readout.

**Responses**: This is another very good point. The calculation method of efferocytosis efficiency is performed as previous study (Cuttell L et al., 2008, Cell, 135(3):524-34). We have described it in Methods and Materials Section in the revised manuscript (**please see** **Page 24, line 652-665**). The positive control, rab5 RNAi-treatments, was more effective than *crq* knockdown. However, crq RNAi treatment still had a significant difference when compared to mock control.

6. Fig 2d: at which time point is crq expression assessed?

**Responses**: We thank the reviewer for this suggestion. *crq* level is tested at 6 h after adding Acs in each RNAi sample cells, and we have added the time point in **Figure 2** **legend**.

7. Page 6/7: the parallel analyses for the two alleles of Bfc should be presented at the same time.

**Responses**: This is a very good suggestion. We have presented the MiMIC insertion of *bfc* and *bfc^ko^* mutants in the same result section in our revised manuscript (**please see** **pages 11, line 281-298**).

8. Why is there more crq mRNA in stage 11 bfc null embryos (fig S3e)? This is not consistent with the author hypothesis.

**Responses**: We apologize for not providing a clearer description of figure S3. There is less *crq* mRNA in stage 11 *bfc* null embryos (**please see** **Fig S3c**). There is more Crq protein in stage 11 bfc null embryos when compared to wild type in **Fig S3e**. One possible explanation is that some Crq undergo post-translational modifications in stage 11 *bfc* null embryos to become more stable. We have added this in the revised manuscript (**please see page 8, line 214-219**).

9. The legend is missing for Fig S3F.

**Responses**: We apologize for this mistake. We have added the legend to **Fig S3f** in the revised manuscript.

10.Figure 3a: the effect of Bcf RNAi on S2 cells AC engulfment is not quantified.

**Responses**: We apologize for not providing a clearer description of Figure 3a. We have quantified the efferocytosis in each RNAi-treatment S2 cells (please see **Figure 2C**), including *crq* and *bfc*. **Figure 3A** was the representative images taken from *crq* and *bfc* RNAi treatment S2 cells. We have added this description to Figure 3A legend and showed the RNAi efficiency in **Figure S1D**.

11. Figure 3e: why is acridine orange staining so low in Bcf KO embryos?

**Responses**: This is another very good point. The Clustering of apoptotic cells represents several corpses within a single macrophage. We can observe them both in wild type and *bfc^ko^* mutants, which means the *bfc^ko^* embryos can successfully engulf corpses. The low AO staining in *bfc^ko^* embryos may be due to the low Crq level in macrophages, which result in less corpses engulfed in a macrophage. And we have showed the number of macrophages did not reduce in *bfc^ko^* embryos (**please see** **Fig S4B-S4C**) by intensifying exposure of Crq staining. And we used fly strain *srp-gal4*>UAS-GFP(BDSC#78565) to mark macrophages and stained Acs by anti-Dcp1. We did not see the difference in macrophage development between WT and *bfc^ko^* (**please see** **Fig S4C and Fig S5**).

12. Page 7: the authors can not conclude that Bfc KO did not affect macrophage development. They should use other markers than Crq to quantify macrophage number / repartition. Actually, with Crq immunostaining (Fig 3f), it seems that there are less macrophages in the head region (and the level of Crq in each cell does not seem reduced as compared to control!).

**Responses**: We thank the reviewer for this very good suggestion. we have tried to use Draper antibody and Pxn antibody made by our lab to label macrophage. However, none worked well in our hands. We have shown the number of macrophages did not reduce in *bfc^ko^* embryos by intensify exposure of Crq staining (**please see** **Fig S4B-S4C**). And we used fly strain *srp-gal4*>UAS-GFP(BDSC#78565) to mark macrophages and stained Acs by anti-Dcp1, we did not see the difference in macrophage development between WT and *bfc^ko^* (**please see** **Fig S4C and Fig S5**)*.* As the reviewer commented, level of Crq in each cell does not seem reduced as compared to control in Figure 3f. This is because we imaged these embryos by using the enhanced similarly to show macrophages. We apologize for not providing a clearer description of experimental conditions for Figure 3a in the original manuscript. We now added the description to Figure 3A legend in the revised manuscript.

13.Page 8: “down-regulation of crq in the absence of Bfc caused the defect in AC clearance”: this is an important point that needs to be formally addressed. In addition to their data that support this hypothesis, the author should rescue Bfc KO by re-expressing Crq and assess AC clearance.

**Responses**: We agree with the reviewer. We have done these experiments, but we did not show the data in our original manuscript. This data is now included as **Figure S6D** and **Figure S6E** in the revised manuscript.

14. Fig 4a: the author should show a higher magnification of Bfc immunostaining together with DAPI or another nuclear marker to demonstrate that Bfc is in the nucleus in vivo. The quality of the present image is clearly insufficient.

**Responses**: We thank the reviewer for this very good suggestion. We used Hoechst 33342 to label the nuclear together with anti-Bfc and anti-Crq in vivo. We can confirm that Bfc is localized in macrophages but not the subcellular localization of Bfc, which may due to the quality of our Bfc antibody or the limited resolution of laser confocal. We have isolated the macrophages from larva and stained them with anti-Bfc to confirm that Bfc is localized in the nuclear (**please see** **Figure 4B”**).

15. Fig 4c: an important negative control is missing (IP with anti-Flag on S2 cells transfected only with HA-Srp). The authors should also try to perform the IP with anti-HA and with the endogenous Bfd (using the anti-Bfd).

**Responses:** This is a very good suggestion. The extracts of HA-Srp transfected S2 cells has been done IP with anti-Flag, which showed no bands of flag- or HA- in the immunoprecipitated products (**please see Figure 4D**). We have tested the Bfc antibody produced by our lab. However, its immunoprecipitation effect was not as good as the commercial Flag antibody, we got too dirty background to judge the results.

16. Fig S5d: this may not be “endogenous” Bfc in S2 cells since these cells are transfected with Bfc-FLAG.

**Responses:** We agree with the reviewer. We have changed Fig S5d in the original manuscript, and stained S2 cells and macrophages with anti-Bfc. This data is now included in new **Figure 4B”** in the revised manuscript.

17. the immunostainings with Bcf and Flag antibodies in Fig S5d look very different from the one shown Fig 4d (GFP-Bfc). This casts doubt on the results. The immunostainings in 4d is very strange as Srp is not known to form such kind of foci. Again some negative controls are missing (single transfections + double immunostaining).

**Responses**: We thank the reviewer for this very good suggestion. We have modified Fig S5d in the original manuscript since tagged Srp form such kind of foci. We have detected endogenous Bfc and endogenous Srp in S2 cells by their antibody. These data are now included in **Figure 4B” and Figure 4E**. But both the antibody of Bfc and Srp were made in mice in our lab, thus we did not show colocalization of Bfc with Srp in the revised manuscript.

18. Page 9 / Figure 5: the authors claim that single heterozygous did not show differences but these results are not shown/quantified. They must be shown.

**RESPONSES:** We apologize for missing these data. We have added these results in **Fig S6E-F** in the revised manuscript (**please see** **page 13, line 357-358**).

19. Page 9: the last paragraph is unclear and not connected with Srp/Bfc interaction

**RESPONSES:** We thank the reviewer for this suggestion. We have changed the heading of this part (**please see page 13, line 348, lines 360-367**).

20. Page 10: the two-hybidrid experiments described here are not displayed Fig S5 but Fig S6. Moreover, there is a clear decrease in the binding of Srp to Bfc when the Cys are mutated to Arg.

**RESPONSES:** We apologize for this mistake. This new data is now included as **S7D-S7E** **Figure** in the revised manuscript (**please see page15, line 398-400**). We performed the two-hybrid assays to test the binding between Srp C4M mutants and Bfc in triplicate experiments, it seemed a decrease in the binding of Srp to Bfc when compared to the binding of ZnF domain of Srp to Bfc. But Srp C4M mutants displayed a similar binding ability to Bfc when compared to the wild type Srp. Our explanation is that other domains of Srp may regulate its binding capacity to Bfc, and removed those structures may lead to the ZnF domain of Srp strongly binding to Bfc. Our two-hybrid assay enables the test of specific protein–protein interactions, but it is not good for efficient, quantitative estimation of their binding affinities. A further experiment is needed to test the binding affinities between Srp C4M mutants and Bfc. We apologize for not including this data in our revised manuscript.

21. Page 10 / Figure 6: the authors claim that they assess Bfc and STAT92E (why?) binding to crq regulatory sequence but the data are not shown.

**RESPONSES:** We apologize for missing this data. We have included this data in **Figure S9**.

22. Page 10/ last line: it is inaccurate to state that “crqM-Gal4 failed to activate transcription”. This driver is just weaker than crq-Gal4. In addition, the authors should test whether crqM-Gal4 activity is (still) affected by Bfc KO and/or srp KO.

**RESPONSES:** We thank the reviewer for this very good suggestion. We have changed the sentence to “Our results showed that the ability of crqM-Gal4 to activate the transcription of crq was much weaker than crq-Gal4” (**please see page 15, line 412-413**). Furthermore, following the reviewer’s suggestion, we have been trying very hard to test the crqM-Gal4 activity in *bfc^ko^* and *srp* mutants. Unfortunately, the transgenic crqM-Gal4 was integrated on Chromosome III, which is the same as *bfc* and *srp* locus. We tried several times but failed to get the recombinant strains of crqM-Gal4/UAS-CrqFlag; *bfc^ko^* or crqM-Gal4/UAS-CrqFlag; *srp* in our hands. We apologize for not being able to show these data in our revised manuscript.

23. The ChIP experiments results are weird. Anti-HA ChIP with non-transfected S2 cells would be a better control. Also it is very unlikely that you can get around 40% of Srp ChIPed to crq as compared to input (even with Histones, people usually end up with a few % of efficiency).

**RESPONSES:** We apologize for this mistake. We have shown the input percent used in ChIP, and added the blank S2 cells without transfection of HA-Srp as a negative control (**please see** **Figure 6C**).

24. The EMSA experiments are ill-described and not performed properly (100% of the probe is shifted in the presence of GST-Srp). The evidence that Bcf promotes Srp binding are very weak. Bfc dose-dependant effect should be demonstrated in EMSA; and reciprocal experiments with Bfc knock-down should be performed. Also it should be checked by western blot that Srp level is not affected upon Bfc overexpression.

**RESPONSES:** We thank the reviewer for this very good suggestion. We have described the control samples and added this part in M&M section (**please see page 28-29, line 780-782**). Moreover, we have repeated the EMSA assay to get more convincing results (**please see Fig 6E-E'**). In addition, we used luciferase assays to show that *bfc* knockdown resulted in a decreased activity of *crq* promoter in HA-Srp transfected S2 cells (**please see Fig 6F**).

25.TheM&M does not give sufficient details concerning many experiments. Notably:

- none of the plasmids used in the transfection assays are described.

**RESPONSES:** We thank the reviewer for this suggestion. We have rephrased our M&M section and have listed the plasmids used in the paper in the revised manuscript (**please see S4 Table**).

26. The RNAseq analysis is poorly described.

**RESPONSES:** We apologize for not providing a clearer description of the RNAseq analysis. We have revised this part in the M&M section in revised manuscript **(please see page 24, lines 639-651**).

27.The efferocytosis assays and quantification is not clearly presented.

**RESPONSES:** We have added the reference of efferocytosis assays and quantification in the revised manuscript **(please see page 10, line 277**) and have now indicated this part more clearly in Material and Methods (**please see** **Page 27, lines 729-736**).

28.Details concerning the anti-Bfc antibody production are missing.

**RESPONSES:** We apologize for this mistake. We have added the details of anti-Bfc antibody production to the M&M section (**please see pages 25-26, lines 691-700**).

29.The EMSA assays are not presented with sufficient details (eg: quantity of poly dIdC, fold excess for crq probe competition…).

**RESPONSES:** The reviewer is absolutely right in pointing our EMSA assays are not presented with sufficient details. We have presented the binding reactions used in EMSA (including poly dIdC) in M&M section (**please see page 28-29, line 775-780, line 790-794**). The binding reaction was following the instruction of LightShift Chemiluminescent EMSA Kit (Thermo #20148).

30. Protocols for nuclear extract preparation and coIP protocol are not given.

**RESPONSES:** We thank the reviewer for this suggestion. We have shown the protocols for nuclear extract preparation in EMSA assays (**please see page 29, lines 790-794**) and added the Co-IP method to the M&M section (**please see page 26, lines 701-708**).

**Minor points**

1.The RNAseq data should have been deposited in a public database and a link provided for reviewer access if the data have not been released yet.

**RESPONSES:** We thank the reviewer for this good suggestion. We have put the raw RNAseq data in NCBI SRA with the accession number: PRJNA733098, the link is <https://dataview.ncbi.nlm.nih.gov/object/PRJNA733098?reviewer=795dqjmq54hu7r2p94m1uq2tbt> which was included in M&M section (**please see page 24, lines 650-651**).

2. the standard deviations for the control situations are apparently missing in several graphs (for example: Fig 1a, c, 1e, 2a, 2b, 2d, 2e, 5f, 6c…); it is thus impossible to evaluate the significance of the differences between control and test situations.

**RESPONSES:** We thank the reviewer for pointing out our neglect in providing standard deviations. We have shown the standard deviations in the “Statistical analysis” part of M&M section, as the graphs with SEM were very important and represented different results in several figures. “*” means P< 0.05; “**” means P<0.01; “***” means P<0.001, “****” means P<0.0001. Error bars in each column diagram represent SEM (**please see page 30, line 810-813**). For the standard deviations of control (e.g.: Fig1a, c, e…), we set the control as 100% in each independent experiment as the graph showed the relative mRNA or protein level, so the standard deviations of control were 0.

3. It is generally accepted that figure calling follow their order of appearance in the main text, which is not always the case here (e.g.: Fig 3a, Fig 4c /d....).

**RESPONSES:** We apologize for this mistake. We have revised the Figures orders and changed them in order in our revised manuscript.

4. Some figures / part of figures are not presented in the text (for example figure 6d right panel).

**RESPONSES:** We thank the reviewer for this suggestion. We have added the figures in the revised manuscript.

5. The arrangement of the panels could be improved to follow a more logical order and facilitate the interpretation by the reader. For example, Figure 2: the “positive” and “negative” genes could be displayed in 2a and 2b respectively and the order of the genes should be kept constant (alphabetical?) in the different panels. Figure 5: place the protein level quantifications below or beside the corresponding western blots and the phagotytic index graphs next to the corresponding images).

**RESPONSES:** We thank the reviewer for this very good suggestion. We have adjusted the genes arrangement in the graphs to make the order in **Figure 2**. We have also changed the arrangement of the panels to make it more logical in **Figure 5**.

6.It is customary to present lateral views of embryos dorsal side down.

**RESPONSES:** We thank the reviewer for this very good suggestion. We have changed the orientation of embryos to present lateral views of embryos dorsal side down.

7.There is a number of recurrent typos; notably: space missing between word and bracket when citing a paper, um instead of µm, CHIP instead of ChIP.

**RESPONSES:** We apologize for these mistakes. We have corrected the writing errors in the revised manuscript.

8. The material and methods sections should be rewritten more clearly, not as a lab protocol, and taking care of correcting grammatical errors as well as scientific mistakes (example: DNA primers do not have a N-terminal but a 5’)

**RESPONSES:** We thank the reviewer for this very good suggestion. We have checked the M&M section and rephrased it in the revised manuscript.

**Reviewer #2**

We are glad that the reviewer #2 appreciated our research findings by commenting “A number of other interesting genes that are modulated via exposure to apoptotic cells are described but not followed up further and these too will be interesting to the field”. He/she further noted: “The paper is very interesting and contains significant amounts of work”. The reviewer also raised some concerns regarding further work is required to strengthen and support the conclusions that have been made. Here are addressing these concerns as follows:

**Major points**

1.I am afraid that I found the manuscript hard to follow in many places. The figure legends and methods are insufficiently detailed and in many places the results are not described well enough to follow what has been done. The methods are written in a note form that is closer to a protocol than the methods section of a publication. Therefore, the entire manuscript would benefit from careful rewriting to ensure sufficient detail is included to aid the reader. This is particularly the case in the section relating to Figure 6 and 7. Additionally, in some places the language used is not grammatically correct or could be improved to help understanding.

**RESPONSES:** We thank the reviewer for this very insightful suggestion. We have rephrased the figure legends and methods with sufficient detail and rewrite the results in the revised manuscript, particularly in the section relating to Figure 6 and 7. We also thank the reviewer very much for reminding us of these grammar flaws in our original manuscript. We have examined the text more carefully and corrected the erroneous sentences as well as other spelling grammar mistakes.

2. The statistics section in the methods is too brief and it appears that inappropriate statistical analyses have been used in many of the figures: according to the methods section it seems that multiple t-tests have been used to compare >2 experimental conditions. Details of the statistical tests used are not described in the figure legends. Therefore, all numerical data and the statistical tests used to compare experimental groups need careful checking and (if needed) a different statistical analysis performing. This may change whether certain data remains statistically significant, so conclusions may need to be modified.

**RESPONSES:** We thank the reviewer for this very good suggestion. We have rephrased the “Statistical analysis” part of M&M section with more detail and described it in the figure legends. We have carefully checked all numerical data and the statistical tests used to compare experimental groups. We used t-test to compare the means of two groups. For the statistics of those results which have more than two experimental samples to be compared were derived using ANOVA in revised manuscript.

3. While I am largely convinced that Srp and Bfc interact and that Srp is binding the crq promoter via a GATA site, I am not convinced that the mechanism by which Bfc is working with Srp is correct or supported by the data presented. This model and Figure 7 are not explained very clearly in the manuscript. I am not sure the data supports the idea that Bfc increases the amount of Srp bound to the crq promoter. However, the way in which the IP and EMSA data are described does not help the reader. Can the authors show that the presence or absence of Bfc regulate the interaction of Srp with the crq promoter (e.g. via ChIP)? For instance: P11 “Bfc interacts with the zinc-finger domain of transcription factor Srp as a co-factor to enhance Srp binding to the crq promoter to elevate its expression and induce efferocytosis in Drosophila melanogaster.” I am not sure that the EMSA data is consistent with this conclusion, but it is not adequately described in the manuscript.

Aditionally, it is not clear what the controls on the left of Fig 6d are or what the panel on the furthest right is demonstrating, as this is not explained anywhere. Authors claim an increase in the migration efficiency of the Srp-DNA complex in the presence of Bfc. Is this the very faint band that can just about be made out below the main band? If so, it is not clear what this means in terms of the mechanism and does not appear to be consistent with Bfc binding to the DNA in concert with Srp, as that should decrease mobility of the band. It is thus not clear how the interaction of Bfc with Srp is functioning to increase Crq levels from this data. Also, why is the Bfc + Srp condition repeated so many times on this gel?

On page 14: “Furthermore, Bfc-Srp interaction occurred through the ZnF GATA domain and that the presence of Bfc increased the ability of Srp to bind the crq promoter.” I don’t think this is substantiated by the data – if it refers to the slight increase in intensity in Fig 6d (not quantified) and this seems an inappropriate conclusion as there is not an excess of DNA (no free probe visible at the bottom). Therefore, probe is rate limiting for complex formation and so adding a fraction more in one lane would change intensity.

**RESPONSES:** We totally agree with the reviewer that the way in which the IP and EMSA data are described does not help the reader. We have reperformed the ChIP experiment and showed the input percent used in ChIP as well as added the blank S2 cells without transfection of HA-Srp as a negative control (**please see Figure 6**). Moreover, we have added the luciferase assays to prove that *bfc* knock-down resulted in a decreased activity of *crq* promoter in HA-Srp transfected S2 cells (**please see Figure 6E-F**). In addition, as instructed by the reviewer, we reperformed the EMSA experiment and found the addition of purified Bfc protein to purified Srp-DNA complex resulted in a super-shift band when compared to Srp-DNA shift band, and the more Bfc were added, the more super-shift band can be observed. (**please see Figure 6 and page 16, lines 442-445**). We described the method in more detail in M&M (**please see pages 28-29, line 774-800**).

4. Authors claim to have analysed STAT92E, but no data is presented: “To detect the physical interactions between regulatory transcription factor proteins and crq in the genome, we then performed yeast one-hybrid (Y1H) assays (Reece-Hoyes and Marian Walhout, 2012) between Bfc, Srp, Stat92E, and the crq promoter locus, respectively.”

**RESPONSES:** We thank the reviewer for this very good suggestion. We have added this data in the revised manuscript (**please see Figure S9**).

5.Apoptotic cell clearance is only analysed in the head. Can defects in apoptotic cell clearance be detected elsewhere in the embryo in bfc mutants? Dcp-1/Crq staining is only shown for the head region. Acridine orange staining looks like it may be less affected in the absence of Bfc along the ventral regions of the embryo. This is particularly important due to the emerging idea that different subtypes of macrophage may be present in the fly (e.g., Cattenoz et al., 2020; Tattikota et al., 2020).

**RESPONSES:** We apologize for not providing a clearer description of our apoptotic cell clearance assay, which was modified from the previous study (Silva E et al., 2007, Immunity 27:585-96). We agree with the reviewer that the amount of engulfment also depends on the location of the macrophages in the stage 13 embryo. Thus, we ensure that measurements are taken from at least five different embryos of any given genotype, and also ensure that we have taken images of macrophages in all three regions (head, tail, and CNS) for calculating the phagocytic index. We have added the details of how we calculated the PI in *Drosophila* embryos in M&M section (**please see page 27, lines 729-736**).

6.Is anti-Crq the best marker to stain and visualise macrophages accurately in bfc mutants if Crq protein levels are so decreased in the absence of Bfc (Figure S5c and other similar figures)? I have always found it difficult to see how phagocytic index is accurately measured using anti-Crq staining in comparison to, for example, use of srpHemo-GAL4, UAS-GFP and other such tools; it is hard to see where one cell begins / ends and how many cells are present in these images. It is also hard to see what is going on due to the overlap of red and green staining in these images (can single channel images be displayed with the merge?) Also, please consider using green/magenta rather than green/red due to those suffering from colourblindness, especially if separate channels are not to be displayed. What do the white dotted circles represent? Individual cells? Lastly, the use of anti-Crq makes it difficult to score these images blindly as mutant and wt/rescues can easily be discriminated and this appears to be quite subjective scoring anyway (precise details are missing from the methods). N.b., these comments apply to all figures that show anti-Crq/anti-Dcp-1 staining.

**RESPONSES:** We thank the reviewer for this very insightful suggestion. We performed our apoptotic cell clearance assay, which was modified from the previous study (Silva E et al., 2007, Immunity 27:585-96). We took confocal Z-stacks of embryos that have been labeled for apoptotic corpses by use of anti-Dcp-1 staining and for macrophages by use of anti-Crq staining. By taking a stack of images through a labeled macrophage, it is possible to count the number of Dcp-1–stained corpses inside one macrophage. The white dotted circles represented individual macrophage. As suggested by the reviewer, we used green/magenta rather than green/red. We agree with the reviewer that by using of *srp*-GAL4, UAS-GFP, it is easy to see where one cell begins and ends. Following the reviewer’s suggestion, we used the fly strain *srp*-gal4>UAS-GFP(BDSC#78565) to mark macrophages and stained corpses by anti-Dcp1.We found the apoptotic cell clearance phenotype such as phagocytic index (PI) was quite similar to our anti-Crq/anti-Dcp-1 staining assay (**please see S5 Figure**).

7.Figure 4d shows Bfc is nuclear in S2 cells. Can a nuclear marker/dye and higher magnification imaging show a similar localisation in macrophages in the embryo? What happens to Srp localisation on RNAi of Bfc from S2 cells? This should be fairly easy to do with the reagents already in hand and may give an insight into how the Bfc-Srp interaction works to regulate Crq levels (especially as I am not sure about the EMSA data).

**RESPONSES:** We agree with the reviewer. We used Hoechst 33342 to label the nuclear together with anti-Bfc and anti-Crq in vivo. We can confirm that Bfc is localized in macrophages but not the subcellular localization of Bfc which may be due to the quality of our Bfc antibody or the limited resolution of laser confocal. We have isolated the macrophages from larva and stained them with anti-Bfc to confirm that Bfc is localized in the nuclear (**please see Figure 4B”**). Moreover, we performed bfc RNAi in S2 cells, but the localization of Srp seemed no different when compared to control (**please see Fig 4E-E'**).

8. Why are immune genes like cecropin coming up in the transcriptional profiling? This seems counterintuitive to the silent clearance of apoptotic cells, as it suggests immune activation. We ourselves have had issues generating apopototic cell samples via similar methods – we obtain apoptotic cells but also a large amount of necrotic cells. The AC samples used/method therefore require characterisation/validation (e.g., caspase activity probes, annexin, PI – all widely available). It therefore is possible that some of the genes that are being upregulated are in response to necrotic cells present in the samples in addition to the apoptotic ones. The AC samples should be characterised and if necrotic cells are present the conclusions need to be modified to acknowledge that the transcriptional changes observed may not be specific to contact with apoptotic cells.

**RESPONSES:** We thank the reviewer for this very good suggestion. As the anti-inflammatory nature of the apoptotic process, factors released from apoptotic cells should suppress inflammation. But there were some references that also showed that some signals from ACs activated the inflammation of macrophages. We added this in the discussion part (**please see page 18, line 493-499**). As instructed by the reviewer, we validated the ACs samples added to live S2 cells and showed that most of them is apoptotic cells rather than necrotic cells (**please see S1A-S1C Figure**). However, we really cannot totally eliminate the up-regulated genes that may be induced by bits of necrotic cells, so we modified the conclusions to acknowledge that the transcriptional changes observed may not be specific to contact with apoptotic cells (**please see page 18, line 485-490**).

9. In general, the discussion needs to discuss the findings in a broader context, as it mostly simply repeats the conclusions/findings of this paper. In particular there is barely any discussion of the work in relation to other organisms (only 1 mammalian paper is cited). For instance, do homologs of Bfc exist in vertebrates? Is this a fly specific mechanism? A careful re-write of this section will make it much more appealing and relevant for non-Drosophilists.

**RESPONSES:** We appreciate the reviewer for pointing out our discussion needs to discuss the findings in a broader context and apologize for our neglect of citing and discussing the work in relation to other organisms. We have now provided a discussion on these points and rephrased the discussion part in the revised manuscript.

**Minor points**

1. Figure 1d-e what does % phagocytosis mean? I presume % of S2 cells phagocytosing AC – please make clearer in legend and methods.

**RESPONSES:** We apologize for not providing a clearer description of our apoptotic cell clearance assay. We made it clearer in legend and added the detailed method in M&M section (**please see page 24, lines 653-665**).

2.I am not sure that it is possible to make the conclusion that “as an engulfment receptor, Crq contributed to early recognition of ACs, while engulfment at later stages likely depended on other regulatory factors to mediate further AC clearance.” It is not clear whether the internalised AC were internalised at earlier times and have not yet been degraded or have been internalised more recently. In my opinion to test this properly you would need to stimulate with AC, remove excess AC and then challenge again, or something similar (if this was actually how the experiment was carried out it is not clear from the results, methods or figure legend). I don't propose this experiment is repeated as suggested, as this doesn't seem a key point, just that the conclusions are modified.

**RESPONSES:** We totally agree with the reviewer that it is not clear whether the internalized AC were internalized at earlier times and have not yet been degraded or have been internalized more recently based on our experiment. We modified the conclusion (**please see page 4-5, line 108-110, line 121-126**).

3.What are the experimental conditions for Figure 2c,d? This is not clear from the text, methods or legend. I presume this is with AC treatment, but for how long etc?

**RESPONSES:** We apologize for not providing a clearer description of experimental conditions for Figure 2c,d. We modified the Figure legend to make it more clearly (**please see page 7, line 188-194**).

4.The effect of knockdown of CG9129/bfc is far stronger than for crq itself (Figure 2c), what other processes may be affected given bfc seems to play no role for SIMU and drpr? It may be nice to speculate on this in the discussion.

**RESPONSES:** We thank the reviewer for this very good suggestion. The effect of knockdown of CG9129/bfc is far stronger than for crq itself, which may be caused by the different RNAi knockdown efficiency of *bfc* and *crq*. We totally agree with the reviewer that although there were no effects on *drpr* and *simu* expression in *bfc^ko^* mutants, other processes may be affected by *bfc,* such as the clearance of neuronal debris and bacterial clearance. We speculate on this in the discussion part.

5.Figure 3a appears after Figure 3c-d. Authors may want to reorder the text / figure slightly to improve how the manuscript reads. There are quite a few panels not cited (e.g. Fig S3e) in the text or mistakes in which figures are being cited throughout the manuscript.

**RESPONSES:** As instructed by the reviewer, we have reordered **Figure 3** in the text (**please see page 8, line 205-208**) and corrected the mistakes in citing the figures.

6.Not all of the inset can be seen in the main field of view for the central panel of Figure 3a, while the zooms could have been a bit bigger. I also struggled to see where the regions were taken from (in fact I am unable to see where the zoom for bfc RNAi came from at all for that field of view).

**RESPONSES:** This is a very good suggestion. We have shown the magnified regions by the white dotted squares in **Figure 3A**.

7.Please provide details of how the mouse anti-Bfc was generated (what epitope was used etc). Authors should check for other methods that are not described in the paper.

**RESPONSES:** As instructed by the reviewer, we have provided details of how the mouse anti-Bfc was generated to the M&M section (**please see pages 25-26, lines 691-700**). We also rephrased our M&M section.

8. Figure 4a - why is the green channel so bright in the bfc[ko] embryo? Have these embryos been imaged using the same settings/enhanced similarly? Why is the green channel so faint in the boxed region of the bfc[ko] panel compared to the main image? Individual channels should be shown and advisable to use magenta in place of red for merges. Also, it would be better not to inset zoomed regions over the posterior of the embryo as this prevents the reader from analysing expression in posterior macrophages.

**RESPONSES:** We apologize for not providing a clearer description of experimental conditions for Figure 4a in the original manuscript. Because we used the intensify exposure of Crq staining to show the macrophages in the *bfc^ko^* embryo. We have imaged WT and *bfc^ko^* embryos by the same settings and used magenta in place of red to show individual channels as well as merges. As instructed by the reviewer, we put the zoomed regions in the upper right corner of each embryo to avoid covering the posterior macrophages (**please see Figure 4A**).

9.References required for this statement on p8: “Previous researches have demonstrated that several signal pathways regulated the expression of engulfment receptor Drpr, such as d-JNK, PI3K and insulin.”

**RESPONSES:** We thank the reviewer for reminding us of this point. We have added the corresponding references in the revised manuscript (**please see page 12, lines 320-321**).

10. page 10 – some confusion / mix ups for figure labelling for Figures S5-7. Not all panels are cited in the text.

**RESPONSES:** We apologize for these mistakes. We have corrected the text in page 10 for **S5** **Figures (now it’s S7 Figure, please see page 14-15**).

11. Why does bfc not work to regulate the amounts of other Srp-dependent receptor genes? Authors suggest other co-factors regulate Srp’s activity at the drpr and simu promoters but I don’t think their model is sufficient to explain why Bfc would not assist at those promoters given their regulation by Srp. Perhaps it would be clearer with more explanation?

**RESPONSES:** This is another very good point. The transcriptional factor Serpent (Srp) was found to be required for sufficient phagocytosis of ACs in Drosophila embryonic macrophages by regulating SIMU, Drpr and Crq (Shlyakhover E et al., 2018, Front Immunol 9:266). However, there is no evidence that Srp directly regulated the expression of SIMU, Drpr and Crq. Moreover, we did not detect the binding of Srp to *drpr* promoter (**Please see S9 Figure**). Another explanation is that Bfc would assist Srp at promoters of *simu* and *drpr*, but have very minor effects, thus we could not detect the effect in *bfc* mutant by our current methods. We added more explanation in the discussion part (**please see page 19, lines 521-525**).

.

12.P13 “These results imply that the expression of phagocytotic receptors could be stimulated by the presence of excessive ACs to improve the phagocytotic activity of macrophages.” This has been shown by a number of other labs in the fly (e.g., Wood and McCall) in flies and is well-documented in vertebrates. These and other papers should be cited and this new mechanism contrasted with how this is regulated in other systems.

**RESPONSES:** We appreciate the reviewer for pointing out our carefulness and apologize for our neglect of citing the findings of other labs in flies and in vertebrates. We have now correctly cited their works and discussed their mechanism in our revised manuscript (**please see page 19, line 508-514**).

13. Authors may wish to include Han et al. (2014) and Guillou et al. (2016) in the discussion since these suggest functions for Crq other than a role as a receptor for apoptotic cells.

**RESPONSES:** We thank the reviewer for reminding us of this point. we have now correctly cited Han and Guillou works in our revised manuscript (**please see page 20, line 554**).

14. Supplemental tables 3 and 4 are not cited in the manuscript.

**RESPONSES:** We apologize for this mistake. We have now correctly cited **S3 Table** and **S4 Table** in the M&M section (**please see page 25, line 671** and **page 26, line 696**).

**Reviewer #3**

**Summary**

Zheng and colleagues have identified a new gene called bfc (booster for Croquemort) in a transcriptomic analysis (RNA-seq) of Drosophila Schneider cells at different time points in efferocytosis followed by an RNAi-mediated approach. bfc is important for efferocytosis, the apoptotic clearance by macrophages, both in vitro and in vivo. The authors found that bfc in macrophages is required for efficient efferocytosis by increasing crq expression due to the interaction with Srp. Bfc physically interacts with srp and has been proposed as a new srp cofactor. The authors show that bfc interacts with the Srp zinc finger domain in a previously unknown manner and that both proteins work cooperatively to improve crq expression due to the binding of srp via a likewise newly identified GATA recognition sequence in the crq promoter.

**Importance of the findings**

My impression is that the publication meets the criteria for publication in PLOS Genetics. The work shows a high degree of originality in the area of genetic control of efferocytosis and is therefore undoubtedly of great importance to researchers in this and the fields of immunology and programmed cell death. In addition to the newly characterized gene Bfc/CG9129, which plays a role in transcriptional activation of Crq via Srp as a potential Srp cofactor, the authors have found further, partially uncharacterized genes for suppressing efferocytosis efficiency. I therefore expect widespread interest from genetic researchers in these disciplines, especially since only further transcription factors as srp cofactors have been identified so far.

The publication has compiled a very large amount of data that is adequately embedded in the literature and employing current methods to show consistent experimentation and evidence to support the conclusions.

**RESPONSES:** We are very glad that the reviewer appreciated the significance and novelty of our findings. He/she also pointed out that our results demonstrate that bfc in macrophages is required for efficient efferocytosis by increasing crq expression due to the interaction with Srp and that our data are potentially suitable for publishing in PLOS Genetics. The reviewer raised concerns regarding some important points in the manuscript that need to be explained further or which could further clarify the results in further experimental approaches. To address these concerns, we have performed additional experiments and provided clearer explanations for our experimental findings in our revised manuscript. Our point-by-point responses to these concerns are as follows:

1. **RNAseq identified 48 genes with similar expression patterns to crq, which was**

**upregulated after incubation with ACs.**

- Please indicate why the threshold levels for crq expression are different from stated in M&M or correct this point in M&M.

**RESPONSES:** We apologize for this mistake and have corrected the threshold levels for *crq* expression in M&M (**please see page 5, line 131-135; page 24, line 646-647**).

-The Figure legend points to number of upregulated transcripts, for me it seems confusing at this place, maybe it is better to show them in the Results section.

**RESPONSES:** We thank the reviewer for this very good suggestion. We have modified the figure legend (**please see page 6, lines 141-142**) and showed them in the results section.

Minor points:

- Please indicate also 1c.

**RESPONSES:** As instructed by the reviewer, we have changed this in the revised manuscript **(please see page 5, line 117)**.

- I am not sure if ”VS” is a general abbreviation for “versus”?

**RESPONSES:** We have changed “VS to “versus”.

2. **Bfc is involved in efferocytosis and regulates crq expression in S2 cells.**

- Please indicate what the criteria to the highly similar expression trend to that of crq are?

**RESPONSES:** We agree with the reviewer. We considered the genes which were up-regulated by adding ACs after 6h and were down-regulated when compared 12h to 6h to have similar expression trend with *crq*. We have showed the significant difference in S2 Table in our original manuscript, we now added these data to the **Figure 2A** and **Figure 2B**.

-“We found the overexpression of CG9129 in S2 cells also up-regulated crq mRNA in the presence of ACs (Figure S2), and…” There is something wrong with this hint and not shown in Figure S2 or S2 is maybe incorrectly labeled. Please explain Figure S2a in more detail and indicate also S2b and correct Figure S2 and the complete Figure Legend and include the Crq-GFP construct and the overexpression in S2 cells in M&M.

**RESPONSES:** We apologize for not providing a clearer description of Figure S2. We have corrected the label (change CrqGFP to Bfc-flag) and legend of **S2 Figure** and provided the constructs information in **S4 Table**. Moreover, we discuss these results in the discussion part (**please see page 20, lines 546-549**).

Minor points:

- Please avoid abbreviations as in the chapter heading when Bfc is used for the first time same as crq in the previous chapter heading.

**RESPONSES:** We have rechecked and changed the inappropriate abbreviations in the revised manuscript.

3. **The expression of Crq is mediated by Bfc in vivo.**

- Please correct the doubled sentences in the middle.

**RESPONSES:** We have modified the sentences (**please see page 8, lines 202-209**).

- Please indicate in M&M how many embryos are used for western blots.

**RESPONSES:** As instructed by the reviewer, we have provided detailed information of how many embryos are used for western blots in M&M section (**please see page 25, lines 681-684**).

-Please correct the heading for Supplement 3 with italics for expression and correct the

heading for Figure S3 to all shown experiments. The expressions in S3a and S3b are reversed and please also state S3f in the figure legend.

**RESPONSES:** We thank the reviewer for pointing out our neglect in providing heading and figure legend for Figure S3. We have corrected the heading and legend of **S3 Figure**.

Minor points:

- Include also S3e in the Results to S3d.

**RESPONSES:** We have added Figure S3e in the results.

- And give an additional hint about S4 in the Results after mentioning simu for the first time.

**RESPONSES:** We agree with the reviewer and have added **S4 Figure** in the revised manuscript (**please see page 9, lines 232-234**).

4. **bfc is required in macrophages for efficient efferocytosis.**

- Can you please add an additional quantification for Figure 3a.

**RESPONSES:** We apologize for not providing a clearer description of Figure 3a. We have quantified the efferocytosis in each RNAi-treatment S2 cells (**Fig 2C**), including *crq* and *bfc*. **Figure 3A** was the representative image from crq and bfc RNAi treatment S2 cells. We have added this to Figure 3A legend and showed the RNAi efficiency in **S1 Figure**.

- The authors introduce an additional MiMIC insertion as a strong LOF bfc allele and

characterize it after generating and describing a newly generated bfc knock-out allele in the previous section of the Results. Please explain why you no longer use the KO allele or combine both descriptions more closely in the text.

**RESPONSES:** We thank the reviewer for this good suggestion. We have presented the MiMIC insertion of *bfc* and *bfc^ko^* mutant in the same result section (**please see page 10-11**).

- Acridin orange staining and efferocytosis phenotype: To me, the number of macrophages in bfc mutants appears to be reduced in 3e, f and also S5c! It would therefore be interesting to compare in Figure 3e also the bfc MiMIC allele and srp (due to the strong reduction of circulating hemocytes) in these acridin orange staining’s and possibly also to quantify the number of hemocytes in bfc mutant embryos. I think that can be an important point to show that the number of hemocytes is not strongly reduced in bfc mutants. In this context, adding of some information’s about both newly generated bfc mutants as viable or lethal would be helpful.

**RESPONSES:** We thank the reviewer for this very insightful suggestion. The clustering of apoptotic cells represents several corpses within a single macrophage. We can observe them both in wild type and *bfc^ko^* mutants, which means the *bfc^ko^* embryos can successfully engulf corpses. The low AO staining in *bfc^ko^* embryos may due to the low Crq level in macrophages, which results in fewer corpses engulfed in a macrophage. Moreover, we have shown the number of macrophages did not reduce in *bfc^ko^* embryos (**please see** **S4B Figure**) by intensify exposure of Crq staining. In addition, we used fly strain *srp-gal4*>UAS-GFP(BDSC#78565) to mark macrophages and stained corpses by anti-Dcp1**(please see S5 Figure)**, and we did not see the difference in macrophage development between WT and *bfc^ko^.*

Minor points:

- Please add reference for PI.

**RESPONSES:** We have added the reference in the text (**please see page 10, line 277**).

- “To further determine if…” probably exchange to “confirm”.

- “reduced 35%” please indicate also 3i.

**RESPONSES:** We thank the reviewer for this good suggestion. We have changed “determine” to “confirm” and indicated 3i for “reduced 35%” **(please see page 11, line 291).**

- Adapt the heading of the figure legend more to the content of the figure (include efferocytosis in S2 cells). Please indicate in all Figures with embryos or in M&M section the orientation of the Drosophila embryos with the dorsal side to the bottom as the agreed orientation is with the dorsal side to the top.

**RESPONSES:** This is a very good suggestion. We have modified the Fig3 legend (**please see page 9**) and explained how we calculated the PI in M&M section (**please see page 27, line 729-736**). And we changed the orientation of the Drosophila embryos with the dorsal side to the top for our figures.

- S5: Please change Heading to ”bfc MI02020 mutants are defective for the efferocytosis of apoptotic cells” and correct spelling errors as “nuclear” and correct to Hoechst also in the Figure S5d.

**RESPONSES:** We apologize for these mistakes. We have changed the heading and corrected the spelling errors for **S5** **Figure**.

5.**Bfc physically interacts with GATA factor Srp.**

- 1 st sentence and later: Is there more information available on Bfc protein structure or domain organization?

**RESPONSES:** We thank the reviewer for this good suggestion. However, Bfc was an as-of-yet uncharacterized protein consisting of 251 amino acids with a predicted molecular weight of 29 kD, which seemed to have no conserved domain based on the bioinformatics analysis. And there is no more information available on Bfc protein structure.

- “Subcellular localization analysis revealed that Bfc-GFP fusion proteins predominantly localized in the nucleus of S2 cells (Figure 4), and Ab staining showed that endogenous Bfc localized in the nucleus of S2 cells (Figure S5d). Since Bfc regulated the transcriptional expression of crq and given its localization in the nucleus, we thus proposed that Bfc may act as a cofactor for the transcription factors of Crq.” Please indicate Figure 4d instead of 4 and again avoid abbreviations as “Ab staining”. But the stating is not shown in Figure S5d due to the overexpression situation in S5d. That means when overexpressing GFP::Bfc then you can´t detect endogenous Bfc or how can you differentiate endogenous from induced Bfc? But this is an important point to show! And probably the easiest way of doing is with the help of the Bfc antibody after induction of efferocytosis and with/without co-expressing mCherry::SRP!

**RESPONSES:** This is another very good point. As suggested by the reviewer, we detected endogenous Bfc by Bfc antibody staining (**please see Figure 4B’ and Figure 4B”** ). We adjusted the figure order, avoided abbreviations, and rephrased this result part (**please see page 12, line 314-317**).

- Please change “yeast two-hybrid screen” to “yeast two-hybrid interaction analysis” as no library was screened.

**RESPONSES:** As instructed by the reviewer, we have changed the sentence.

- Please explain the Results of Figure 4c and not only mention the Figure “We further confirmed the Bfc interaction with Srp in co-transfected S2 cells using Bfc-FLAG- and HA-Srp-tagged constructs (Figure 4c).” and please describe Co-IPs also in the M&M section.

**RESPONSES:** We thank the reviewer for this good suggestion. We modified the Figure 4 Legend, described CO-IP method in M&M section (**please see page 26, line 701-708**), and listed corresponding plasmids in **S4 Table**.

- “Bfc co-localized with Srp” please indicate “tagged Bfc co-localized with tagged-Srp”.

- Figure 4a: Please correct the orientation of the Drosophila embryos with the anterior to theleft as in all other embryo pictures. 4d: Correct also Bfc to GFP::Bfc and Srp to mCherry::Srp and Hochest to Hoechst.

- Include the generation of GFP::Bfc, Bfc::Flag and mCherry::Srp in the M&M section, possibly in a new subsection “S2 transformation vectors”.

**RESPONSES:** We thank the reviewer for this good suggestion. As commented by Reviewer 2#, tagged-Srp is very strange as Srp is not known to form such kind of foci. We have modified Figure S5d in our original manuscript. We have detected endogenous Bfc and endogenous Srp in S2 cells by their antibody. These data are now included in **Figure 4B” and Figure 4E**. But both the antibody of Bfc and Srp were made in mice, thus we did not show colocalization of Bfc with Srp in the revised manuscript. We have corrected the orientation of the *Drosophila* embryos in **Figure 4A**.

Minor points:

- Figure Legend 4a: it is already stated in the M&M section in which animals the antibodies are made, please indicate here only in which color the 2nd antibodies were detected.

**RESPONSES:** We thank the reviewer for this suggestion. We have rephrased Figure 4a legend.

6.**Bfc acts as a co-factor for the Srp transcription factor to regulate Crq expression**

**and efferocytosis.**

- Please indicate in the Results section, the Figure and Figure Legend the used srp allele.

**RESPONSES:** We agree with the reviewer. We got *srp* mutant from Bloomington stock and mentioned it according to the corresponding reference. We have indicated *srp* allele in the Results section, the Figure and Figure Legend.

- “The results showed Crq levels were decreased in the bfc ko and srp double heterozygous embryos (Figure 5a to c).” And also in srp or?

**RESPONSES:** We have modified this (**please see page 13, lines 352-354**).

- “single heterozygous macrophages had no clear phenotypic difference from wild type”,

please indicate “data not shown”.

**RESPONSES:** We have added the PI results for single heterozygous macrophages **(please see pages 13, lines 357-358, S6F Fig).**

- Please instead of “obtained … mutants” use “generated mutants … with Crispr/cas9

mutagenesis”

**RESPONSES:** As instructed by the reviewer, we have changed the sentence.

Minor points:

- Please indicate the generation of crq ko also in the M&M section

- Please indicate in the M&M section by Statistical analysis what 4 **** mean in 5f and 5i?

**RESPONSES:** We thank the reviewer for this suggestion. We have added the detailed method on how to generate *crq^ko^* mutant in the M&M section (**please see page 23, line 617-626**) and have explained the meaning of “****” in Statistical analysis (**please see page 30, lines 812**).

7. **Srp directly binds the crq promoter through GATA site recognition enhanced by Bfc.**

- Please change S5a to S6a, S5b to S6b, S5e to S6e and indicate S6d when speaking about mutations in the ZnF domain and avoid abbreviations as “ZnF” before using the complete word for the first time.

**RESPONSES:** We apologize for these mistakes. We have modified this part in the revised manuscript.

- Please include the shown Co-IP results from S6c in the results section.

**RESPONSES:** As suggested by the reviewer, we have added Co-IP results from **new S7C Figure** to the result section **(please see page 14-15, lines 395-396).**

- Immunofluorescence and WBs with FLAG-tagged proteins are shown in S7a and b, not in S6a and b.

**RESPONSES:** We have corrected this in revised manuscript.

- “…Immunofluorescent staining showed that the ectopic expression of Crq-Flag was much lower than that in the control…” An important point is how it is ensured that the newly generated and mutated crq::GAL4 construct shows the same expression as the Bloomington crq-GAL4 driver?

**RESPONSES:** We agree with the reviewer. We generated mutated *crq-gal4* based on the sequence reported by the Bloomington *crq-gal4*, and only mutated the GATA site of *crq-gal4* driver, the construct was sequenced right. We integrated crq-GAL4 and crqM-Gal4 constructs into the same genomic location by site directed insertion methods. We have added the detailed methods in M&M section in our revised manuscript.

- Y1H assays: Please indicate “data not shown” for Bfc and Stat.

**RESPONSES:** Thanks for the suggestion. We have added this data in **S9 Fig** (**please see page 16, line 452**).

- “The Y1H results showed that only Srp-pADT7 interacted…” Please indicate Srp-AD as in the Figure.

**RESPONSES:** As instructed by the reviewer, we have changed the sentence (**please see page 15, line 403**).

- “Sequence analysis of the -1000 bp crq promoter element led to the discovery…” please change to “ and further sequence analysis of the -1600 bp promotor led to the…” as shown in the Figure and indicate in the Figure 1.6 kb instead of 1.6k.

**RESPONSES:** We thank the reviewer for this suggestion. We have changed the sentence (**please see page 15, line 405**) and indicated in the Figure 1.6 kb.

- “To confirm that the GATA sequence in the crq promoter is required for crq expression, we mutated the Srp binding site in the GATA sequence and integrated this crqM-Gal4 construct into the same genomic location to drive Crq-Flag expression in the wild type.” I am confused by this sentences. What do you mean with “the same genomic location”? The same as what? I assume as crq-GAL4? I missed that in the M&M section? However, your M&M section describe the Bloomington crq-GAL4 driver and how did you know where the construct is located and how can you made sure if both driver lines produce the same strong signal (see above)?

**RESPONSES:** We thank the reviewer for this very good suggestion. We generated mutated *crq-gal4* based on the sequence reported by the Bloomington *crq-gal4*, and only mutated the GATA site of *crq-gal4* driver, the construct was sequenced right. We integrated crq-GAL4 and crqM-Gal4 constructs into the same genomic location by site directed insertion methods. We have rephrased this sentence and added the detailed methods in M&M section in our revised manuscript.

- “Our results showed that crqM-Gal4 failed to activate the transcription of crq (Figure S7a and c)“ Presumably because of the mutated GATA site, it cannot activate all other UAS constructs such as unspecific UAS-lacZ or UAS-GFP constructs, right?

**RESPONSES:** We thank the reviewer for this very insightful suggestion. We used crqM-Gal4 to drive CrqFlag, and there was a weak band in crqM-Gal4/UAS-CrqFlag when we tested Flag level by western blot. It is not accurate to conclude that crqM-Gal4 failed to activate the transcription of *crq*, so we have changed the sentence to “Our results showed that the ability of crqM-Gal4 to activate the transcription of *crq* was much weaker than crq-Gal4” (**please see page 15, line 412-413**).

- “Moreover, CHIP qPCR assays confirmed that Srp protein GATA sequence exhibits high specificity binding to the crq promoter (Figure 6c)…” What do you mean with that?

**RESPONSES:** We apologize for not clear description of Figure 6c. We have revised the sentence to make it clearer **(please see page 15, line 414-418).**

- The ChIP assay in Figure 6d: please indicate n! Is it usual to compare the whole input with the HA-beads with p-value? I know the way to show control and HA as percent of input and comparing control to HA with p-values.

**RESPONSES:** We apologize for this mistake. We have showed the input percent used in CHIP, and added the S2 cells without transfection of HA-Srp as a negative control (**please see Figure 6**).

Minor points:

- Why are the Figure Legends of S6 and S7 no longer in block set as all other Figure Legends?

- “In addition, the mutated Srp ZnF from Cys to Arg eliminated binding to the crq promoter (Figure S6f).” Please indicate also S6d in the Results section.

**RESPONSES:** We apologize for this mistake. We have modified the Figure Legends of S6 and S7 and have added S6d to the result (now **S7 Figure**) (**please see page 15, line 399-400, line 407-408**).

- Please correct Figure Legend heading S7: “The mutated GATA sites result to defective

phagocytotic ability in Drosophila embryos.” That is not shown in S7 since only expression patterns are traced!

**RESPONSES:** We apologize for this mistake. We have corrected the Figure Legend heading S7 to “The mutated GATA sites fail to activate Crq expression in *Drosophila* embryos”.

- Please indicate also S7d-f in the Results section.

**RESPONSES:** We have added S8d-f to the result section (now it is **S8** **Figure**) (**please see page 15, lines 412-414**).

- Figure Legend 6: “black arrow head indicates…” Please correct the black arrow head to a“red arrow”

**RESPONSES:** As instructed by the reviewer, we have corrected it (**please see page 15, line 421**).

- “Positive and negative control were present. Yeast cultures were diluted (1:10 successive dilution series), spotted onto selective medium containing 300 ng/ml ABA and no Histidine (-His), then incubated for 3 d at 30°C.” please put that information’s to the M&M section.

**RESPONSES:** As suggested by the reviewer, we have put this information in the M&M section (**please see page 27-28, line 745-756**).

- For the EMSA assay in M&M, please indicate which control exists, for what the S2

nucleoprotein (correct Nuceo to Nucleo) and the anti-HA were used?

**RESPONSES:** We thank the reviewer for this suggestion. We have explained the control samples (**please** **page 28-29, lines 780-782**) and which nucleoprotein was used in M&M section (**please see page 29, lines 790-792**). Nucleoprotein was extracted from HA-Srp-transfected S2 cells.

8. **Discussion**

- “Here, we show a novel gene, bfc (Booster for Crq), that specifically regulates crq expression in a manner dependent the amount of apoptosis…” Please revise language.

**RESPONSES:** This is a good suggestion. We have rephrased the sentences (**please see page 17, line 465-467**).

- “Then, in vivo we found that developmentally programmed apoptosis, and during the first wave of apoptosis beginning at stage 11, when macrophage develop, crq expression is activated and subsequently becomes widespread throughout the 12 embryo.” Dito.

**RESPONSES:** We thank the reviewer for this good suggestion. We have rephrased the sentences in revised manuscript (**please see page 17, line 473-476**).

- “We found crq had a curve expression trend in S2 cells incubated with ACs…” what does curved expression mean?

**RESPONSES:** We thank the reviewer for pointing out our neglect in providing a more accurate statement regarding the crq expression. we have rephrased our description in revised manuscript (**please see page 18, line 482-483**).

- Please indicate Figure 7 in the Discussion section.

**RESPONSES:** As suggested by the reviewer, we have indicated **Figure 7** in the last paragraph of discussion section.

- Please completely revise the language of Figure Legend 7 and change Acs to ACs.

- Figure Legend 7: “bfc is transcriptionally activated earlier than crq…”. AND “We found that up-regulation of bfc expression occurred earlier than crq in S2 cells incubated with ACs…”Please refer to the corresponding Result illustration.

**RESPONSES:** We thank the reviewer for this good suggestion. We have completely revised the language of Figure Legend 7 and refer to the corresponding figure in the discussion (**please see page 21, line 568-570**).

- In the paragraph on the role of srp and crq transcription I missed a reference to the already known srp cofactors and what distinguishes them from Bfc.

**RESPONSES:** We appreciate the reviewer for pointing out our carefulness and apologize for our neglect of citing and discussing the potential role of Ush(FOG-2), the already known srp cofactor in *Drosophila*. We have discussed the difference between Ush and Bfc in the discussion part (**please see pages 19-20, lines 535-541**).

9. **Material & Methods**

Some parts of M&M section (e.g. “Apoptotic cells preparation…”) is incomprehensible to non-familiar readers. “Observe efferocytosis und microscope (Zeiss)”? Please indicate microscope and used software etc. For RNAi treatment: “exact S2 cells DNA templates”? “Phagocytotic cells after RNAi treatment were collected for qPCR…” only these cells or all cells? The section needs a revision in the description of methodology and please use the same way for all units all over the section. Please indicate for the Western Blots on embryos how many embryos you used for protein extracts? Please revise the AO staining. Please indicate the reference for phagocytic index.

**RESPONSES:** We apologize for not providing a clearer description in M&M section. As suggested by the reviewer, we have revised the part of apoptotic cells preparation (**please see page 24, lines 652-665**) and added the detailed methods in M&M section. We have indicated the reference for phagocytic index in the revised manuscript.

Please include the production of the bfc antibody to the M&M section, also the antibody staining on S2 cell cultures and Drosophila embryos and include all used antibodies (Flag, mCherry, etc.). Describe Co-IPs and include the generation of all constructs (e.g. UAS-crqFLAG, GFP::Bfc, Bfc::Flag and mCherry::Srp)!

For what purpose you need the bfc sgRNA? Please refer in the Result section.

Check whether the order of methods can be improved.

**RESPONSES:** We thank the reviewer for this good suggestion. We have added the details on how to produce antibodies to the M&M section (**please see pages 25-26, lines 691-700**). Co-IP method was also added to the M&M section (**please see pages 26, lines 701-708**). The generation of all constructs was described in **S4 Table**.

10.**English**

Correct several spelling errors all over the manuscript and several expressions, some

examples are stated here:

Graph shows the ratio of twenty-four gene mRNA expression in each gene RNAi treated S2

cells when compared to control-treated control S2 cells, as determined by qPCR…

Which consistent with previous study…

Moreover, CHIP qPCR assays confirmed that Srp protein GATA sequence exhibits high

specificity binding to the crq promoter…

**RESPONSES:** We thank the reviewer very much for reminding us of these grammar flaws in our original manuscript. We have examined the text more carefully and corrected the erroneous sentences as well as other spelling grammar mistakes. We have rephrased some sentences and paragraphs to provide better explanations for the rationale and conclusions of our experimental findings.
