# Supplementary material for: bfc, a novel serpent co-factor for the expression of croquemort, regulates efferocytosis in Drosophila melanogaster
Source: PLoS Genet. 2021 Dec 3;17(12):e1009947. doi: 10.1371/journal.pgen.1009947 (PMC8673676; doi:10.1371/journal.pgen.1009947)
Supplement: Attachment — Submitted filename: response_to_reviewers_21.9.25.docx [file pgen.1009947.s021.docx]

**Reviewer #1:**

In their revised manuscript, Zheng et al. have answered some of the concerns raised by the 3 referees and have improved the overall quality of their work. Yet, beside the presentation of the data and M&M that needs to be improved, several conclusions are not well substantiated and some experiments are still not performed adequately. In addition, some of the modifications introduced during the revision do not speak in favor of a rigorous analysis of the results by the authors.
Thus, even though the authors made important modifications to their manuscript, I still think that it does not reach the standards of quality expected for publications in PLoS Genetics.
**RESPONSE:** First of all, we thank the Reviewer for the time spent evaluating our manuscript, and for the pertinent questions and constructive criticisms. We have read all of your comments and tried our best to address them, experimentally. We believe the new data, based on the reviewer suggestions further support our conclusions, and thus thank the reviewer for the feedback that helped us to improve our manuscript. We hope now that the manuscript meets the standards of quality expected for publication in PLoS Genetics.


Some specific comments to the authors.

Major points:

- The statistical significance of many results shown in Figure 2 and S1 have changed as compared to the original version of the manuscript even though they are based on the same numerical data. While I understand that the authors have now used different methods (Annova), I’m very skeptical as some points “gained” one or even two stars of significance and other lost up to three stars!! (e.g: pgrpSA at 12h was “ns” and is now p<0.01; CG8907 at 6 and 12h were p<0.01 and are now ns!). This casts important doubts on the analyses of the data by the authors.

**RESPONSE:** We apologize for not providing a clearer description of the qPCR data in Figure 2A. As we screened the RNA-seq and qPCR data looking at the genes differentially expressed at 6h>0h and 6h>12h, we have only shown the significant differences in these two contexts. For the sake of accuracy, we have clarified this in the revised legend of Figure 2A.

- The authors claim that bfc mutation does not affect macrophage development but the data are still not convincing (S4B-C). Notably, the authors used srp-GAL4>GFP to label macrophages and they observed around 100 macrophage per embryo in wildtype and bfc mutant contexts. This is far less than expected as wildtype embryos contains several hundreds of macrophages!

**RESPONSE:** We thank the reviewer for the remark. We have cross-checked the macrophage numbers in the embryos again; there were approximately 400 macrophages.

- The rescue experiment with crq-GAL4 UAS-Crq was performed in the hypomorphic bfc mutant and not in the null context (S6). As the authors showed that Crq induces bfc transcription, it is thus difficult to conclude that it the restauration of Crq level rather than the restauration of Bfc level which is responsible for the rescue.

**RESPONSE:** We understand the reviewer’s doubt. To clarify it, we used the null *bfc* mutant(*bfc^ko^* which was generated in this paper) for the rescue experiment with *crq-GAL4>UAS-Crq*; notably, the rescue was caused by the restoration of the Crq levels rather than the restoration of the Bfc levels **(Fig S6D)**.

- The impact of apoptosis/ACs on Srp/Bcf-mediated regulation of crq is not clearly assessed. Notably, the authors do not formally test that apoptosis is important for the induction of Bfc and/or Crq expression in vivo. Actually, line 213/FigS3B, the authors show that bcf expression is upregulated as early as stage 9, i.e. before the first wave of apoptosis (stage 11). The conclusion lines 235-236 is not substantiated by the in vivo data. The authors could use the H99 deletion to prevent apoptosis in the embryo and assess whether this affects bfc and crq induction. Thus far, the conclusion line 505-506 is not really sustained.

**RESPONSE:** We agree with the reviewer’s suggestion. To further confirm that the upregulation of crq and bfc was induced by ACs, we quantified the *crq* and *bfc* mRNA levels in *H99* embryos from stage 10 to 13. Importantly the expression levels remained at lower levels compared to those in the wild type condition **(S3F Fig)**.

- The hypothesis that Bfc promotes Srp binding to crq regulatory region is still poorly substantiated. First, the results of the EMSA are not convincing: the supershift with Bcf/Srp is barely detectable and does not really increases with Bcf dose. The binding of Srp to crq probe seems highly variable (cf 2E and 2E’); it may increase with Bcf but this could reflect a mere stabilization due to increased protein concentration in the reaction (increased Srp binding is also observed when anti-HA antibody is added to the reaction in 2E’). Also, line 7 does not seem to contain any probe, and the competition with the unlabeled crq probe does not seem to work in panel 2E. Second, the authors did not test the influence of Bfc on Srp recruitment to crq region by ChIP and they did not assess whether Bfc over-expression/knock-down affects Srp protein level (by western blot). Again these experiments (ChIP, luciferase assays, western blots) should be performed in S2 cells exposed to ACs to be able to conclude on the molecular mechanisms by which ACs control crq expression (lines 469-470).

**RESPONSE:** We agree with the reviewer’s suggestion. We repeated the EMSA assay **(Fig 6F to 6F’)**, compared the ChIP signal in S2 cells without ACs with that in S2 cells with ACs, and overexpressed Bfc in HA-Srp-transfected S2 cells to test the influence of Bfc on the recruitment of Srp to the crq region, in line with your suggestions **(Fig 6C and 6D)**. Of note, the overexpression of Bfc did not affect the Srp protein levels either in the presence or absence of ACs **(S9D-E Fig)**. Based on the abovementioned data, we have revised the Results and Figure Legends **(lines 468-491)**.

Minor points:

- Lines 120-123: I don’t see a particularly logical link between ACs clearance and bacteria uptake. Even if there is one, the level of Crq is lower at 12h than at 6h but efferocytosis is higher at 12h

**RESPONSE:** We agree with the reviewer’s suggestion. We have rewritten this part**(line 119-123).**

- In the discussion: lines 550-552 and 562-565 are identical.

**RESPONSE:** We are sorry for the mistake; we have corrected this part, for accuracy.

- Lines 560: references are missing concerning the regulation of crq by Srp. Also, when discussing the role of Ush/Srp (lines 538-541): the induction of crq by Srp was shown to be repressed by Ush in Waltzer et al EMBO J 2002.

**RESPONSE:** We thank the reviewer for this very insightful suggestion. We added this discussion in **line 579-582**.

- Some parts of the material and methods are still written in a note form closer to a protocol than the methods sections of a publications (e.g.: lines 697-699; 723-728; 746-751; 758-770; 778-779

**RESPONSE:** We availed the services of a professional scientific English language editing company (Editage), to improve the readability and flow of the manuscript text. We believe that now the Materials and Methods section is written in a more clear and consistent way.

- I did not find the GO annotations data for the RNA-seq in the supplemental data (lines 649).

**RESPONSE:** We apologize for this oversight. In the revised manuscript, we have analyzed and discussed differentially expressed genes after filtering instead **(lines 696-697)**.

- It is still not clearly mentioned in the M&M that the authors generated not only a crqM-GAL4 but also the crq-GAL4 “wildtype” version, integrated at the same site an in the description of the results/figure legends, it should be clearly mentioned when the authors use Bloomington crq-GAL4 or their crq/crqM-GAL4 lines.

**RESPONSE:** We apologize for the vague description of the generation of crqM-GAL4. We have revised the M&M section, for accuracy **(lines 643-654)**.

- In their answer to reviewer 1, the authors claim that they could not recombine crqM-GAL4 with bfc or srp; yet according to their M&M crqM-GAL4 is on chromosome II, so it shouldn’t be a problem. And if it is on chr III, it should be possible to obtain recombinant at least with one of the two genes (bfc or srp) as they are on opposite chromosomal arms.

**RESPONSES:** We thank the reviewer for this insightful comment. As an alternative approach, we derived a *UAS-GFP* construct and detected the GFP levels in the context of *crq-Gal4/UAS-GFP*, and *crq^M^-Gal4/UAS-GFP*. A weaker GFP signal was detected in the context of *crq^M^-Gal4/UAS-GFP*, suggesting that *crq^M^-Gal4* activated the unspecific protein **(S9A-S9C Fig)**. Furthermore, following the reviewer’s suggestion, we have been retrying very hard to test the crq^M^-Gal4 activity in *bfc^ko^* and *srp* mutants. We get the recombinant strains of *crqM-Gal4/UAS-GFP*; *bfc^ko^* but fail to the *crqM-Gal4/UAS-GFP*; *srp* in our hands. We found that crqM-Gal4 activity is not affected by *bfc ^ko^***(S9A-S9C Fig, line 435-437).**

- Figure 4D: the negative controls (right panel) should have been performed in the same experiment and not added afterward to the original coIP (left panel).

**RESPONSE:** Thank you for the suggestion. We performed a new experiment and the data has been included **(Fig 4D)**.

- Figure S7C: part of the anti-HA western blot (lanes HA-Srp4) is visibly spliced from another gel.

**RESPONSE:** Thank you for the remark. Because the truncated Srp is not of the same size, we spliced the image to align them. Now, we have replaced the spliced image with the original one, for accuracy **(Fig S7C)**.

**Reviewer 2**

The authors have largely satisfied my queries and the manuscript is much improved. Some minor points remain to be resolved before the manuscript can be accepted in my opinion but these should be easy to address. I hope the authors have found my comments constructive and helpful; I have enjoyed reading this paper. My comments follow the lead author's response to my original comments and are prefixed by "Reviewer:"

**RESPONSE:** First of all, thank you for the time spent reviewing our manuscript. Your feedback has helped us to considerable improve our manuscript, and for that we are deeply grateful. Once again, we have considered all the new comments. Please check below our point-by-point responses to them.

Major points
1.I am afraid that I found the manuscript hard to follow in many places. The figure legends and methods are insufficiently detailed and in many places the results are not described well enough to follow what has been done. The methods are written in a note form that is closer to a protocol than the methods section of a publication. Therefore, the entire manuscript would benefit from careful rewriting to ensure sufficient detail is included to aid the reader. This is particularly the case in the section relating to Figure 6 and 7. Additionally, in some places the language used is not grammatically correct or could be improved to help understanding.
**RESPONSES:** We thank the reviewer for this insightful suggestion. We have rephrased the figure legends and methods with sufficient detail and rewritten the results in the revised manuscript, particularly in the section relating to Figure 6 and 7. We also thank the reviewer for highlighting grammatical flaws in our original manuscript. We have examined the text carefully and corrected the erroneous sentences as well as other spelling mistakes.
**Reviewer: The manuscript is much improved but some sections still need attention and very careful copy editing. For example, several sections of the methods remain in note form as per a protocol (e.g. AO, ChIP and Y1H sections). There are quite a few typos or sentences in which words are missing making it hard to follow in places.**

**RESPONSE:** We procured the services of a professional English language editing company (Editage), to improve the readability and flow of the manuscript text. We believe now the Materials and Methods section is written in a clearer and consistent way, and the grammatical inconsistencies have been removed.

2. The statistics section in the methods is too brief and it appears that inappropriate statistical analyses have been used in many of the figures: according to the methods section it seems that multiple t-tests have been used to compare >2 experimental conditions. Details of the statistical tests used are not described in the figure legends. Therefore, all numerical data and the statistical tests used to compare experimental groups need careful checking and (if needed) a different statistical analysis performing. This may change whether certain data remains statistically significant, so conclusions may need to be modified.
**RESPONSES:** We thank the reviewer for this suggestion. We have rephrased the “Statistical analysis” part of M&M section with more detail and described it in the figure legends. We have carefully checked all numerical data and the statistical tests used to compare experimental groups. We used t-test to compare the means of two groups. For the statistical analysis of those results, which had more than two experimental samples to be compared, ANOVA was used and this has been updated in the revised manuscript.
**Reviewer: If using ANOVA’s please state the post-test used in the Figure legends.
Otherwise, point 2 has been addressed. Should Fig 2E not also be a 2-way ANOVA
rather than a 1-way ANOVA? Maybe this is a mistake in the legend? Fig 3C it is
probably ok to use a** **t-test rather than an ANOVA (as written in the legend at least).**

**RESPONSE:** We thank the reviewer for this suggestion. We have rephrased the statistical analysis accordingly.

3. While I am largely convinced that Srp and Bfc interact and that Srp is binding the crq promoter via a GATA site, I am not convinced that the mechanism by which Bfc is working with Srp is correct or supported by the data presented. This model and Figure 7 are not explained very clearly in the manuscript. I am not sure the data supports the idea that Bfc increases the amount of Srp bound to the crq promoter. However, the way in which the IP and EMSA data are described does not help the reader. Can the authors show that the presence or absence of Bfc regulate the interaction of Srp with the crq promoter (e.g. via ChIP)? For instance: P11 “Bfc interacts with the zinc-finger domain of transcription factor Srp as a co-factor to enhance Srp binding to the crq promoter to elevate its expression and induce efferocytosis in Drosophila melanogaster.” I am not sure that the EMSA data is consistent with this conclusion, but it is not adequately described in the manuscript.

Aditionally, it is not clear what the controls on the left of Fig 6d are or what the panel on the furthest right is demonstrating, as this is not explained anywhere. Authors claim an increase in the migration efficiency of the Srp-DNA complex in the presence of Bfc. Is this the very faint band that can just about be made out below the main band? If so, it is not clear what this means in terms of the mechanism and does not appear to be consistent with Bfc binding to the DNA in concert with Srp, as that should decrease mobility of the band. It is thus not clear how the interaction of Bfc with Srp is functioning to increase Crq levels from this data. Also, why is the Bfc + Srp condition repeated so many times on this gel?
On page 14: “Furthermore, Bfc-Srp interaction occurred through the ZnF GATA domain and that the presence of Bfc increased the ability of Srp to bind the crq promoter.” I don’t think this is substantiated by the data – if it refers to the slight increase in intensity in Fig 6d (not quantified) and this seems an inappropriate conclusion as there is not an excess of DNA (no free probe visible at the bottom). Therefore, probe is rate limiting for complex formation and so adding a fraction more in one lane would change intensity.
RESPONSES: We completely agree with the reviewer that IP and EMSA description and results were not clear. For manuscript revision, we repeated the ChIP experiment and have shown the input percent used in ChIP as well as added the blank S2 cells without transfection of HA-Srp as a negative control (please see **Figure 6C-D**). Moreover, we have included the luciferase assays to prove that bfc knock-down resulted in a decreased activity of crq promoter in HA-Srp transfected S2 cells (please see **Figure 6E-F**). In addition, as suggested, we repeated the EMSA experiment and found the addition of purified Bfc protein to purified Srp-DNA complex resulted in a super-shifted band compared to Srp-DNA shift band, and addition of increased amount of Bfc increased the super-shift of the band (please see **Figure 6F and page 16, lines 481-485**). We have described the method in more detail in M&M **(line 848-858)**.
**Reviewer: The new ChIP data with additional controls etc. supports the conclusions more effectively and shows a requirement for Bfc in the effects of Srp-HA overexpression on crq promoter activity. The new data more fully supports the original conclusions but the paragraph describing this part of the project could still be written more clearly. If there is an issue with the word count, I suggest the editors relax word count restrictions or other sections are abridged to enable a fuller, more clear description of these important experiments. There are several typos in this section. E.g., line 439 used > using; missing “extract” after nuclear from this line? Line 440 missing “DNA/promoter” after crq? Figure 6E’ described before 6E in the text – consider reordering the text or the figure.**

**RESPONSE:** We thank the reviewer for the suggestion. For accuracy, we have explained Figure 6E in detail in the Results section of the revised manuscript **(lines 438-445)**. Do note that we procured the services of a professional scientific English language editing company (Editage), to improve the readability and flow of the manuscript text. We believe now the text is written in a clearer and consistent way, and the grammatical inconsistencies have been removed.

4. Authors claim to have analysed STAT92E, but no data is presented: “To detect the
physical interactions between regulatory transcription factor proteins and crq in the genome, we then performed yeast one-hybrid (Y1H) assays (Reece-Hoyes and Marian Walhout, 2012) between Bfc, Srp, Stat92E, and the crq promoter locus, respectively.”
RESPONSES: We thank the reviewer for highlighting this and we apologize for the oversigt. We have added this data to the revised manuscript (please see Figure S9).
**Reviewer: Thank you for your consideration.**

**RESPONSE:** We are glad that our previous revisions addressed your concerns. Thank you again for the valuable feedback.

5.Apoptotic cell clearance is only analysed in the head. Can defects in apoptotic cell
clearance be detected elsewhere in the embryo in bfc mutants? Dcp-1/Crq staining is only shown for the head region. Acridine orange staining looks like it may be less affected in the absence of Bfc along the ventral regions of the embryo. This is particularly important due to the emerging idea that different subtypes of macrophage may be present in the fly (e.g., Cattenoz et al., 2020; Tattikota et al., 2020).
RESPONSES: We apologize for not providing a clearer description of apoptotic cell clearance assay, which was modified from the previous study (Silva E et al., 2007, Immunity 27:585-96). We agree with the reviewer that the amount of engulfment also depends on the location of the macrophages in the stage 13 embryo. Thus, we ensured that measurements were taken from at least five different embryos of any given genotype, and imaged macrophages in all three regions (head, tail, and CNS) for calculating the phagocytic index. We have added the details of how we calculated the PI in Drosophila embryos in M&M section (please see page 27, lines 729-736).
**Reviewer: Authors should confirm with editor that the methods describe the original analysis of PI rather than a new analysis since the graphs have not changed between submissions. Otherwise I am satisfied with this.**

**RESPONSE:** We thank the reviewer for the remark. Now, for the sake of clarity, we have changede the citation in the M&M section of the revised manuscript **(lines 786-787)**.

6.Is anti-Crq the best marker to stain and visualize macrophages accurately in bfc mutants if Crq protein levels are decreased in the absence of Bfc (Figure S5c and other similar figures)? I have always found it difficult to see how the phagocytic index is accurately measured using anti-Crq staining in comparison to, for example, the use of srpHemo-GAL4, UAS-GFP, and other such tools; it is hard to see where one cell begins or ends and how many cells are present in these images. It is also difficult to see what is going on due to the overlap of red and green staining in these images (can single channel images be displayed with the merge?) Also, please consider using green/magenta rather than green/red due to those suffering from colourblindness, especially if separate channels are not to be displayed. What are the white dotted circles represent? Individual cells? Lastly, the use of anti-Crq makes it difficult to score these images blindly because mutant and wt/rescues can easily be discriminated, and this appears to be quite subjective scoring anyway (precise details are missing from the methods).
N.b., these comments apply to all figures that show anti-Crq/anti-Dcp-1 staining.
RESPONSES: We thank the reviewer for this insightful suggestion. We performed apoptotic cell clearance assay, with slight modifications from a previous study (Silva E et al., 2007, Immunity 27:585-96). We used confocal Z-stacks of embryos that were labeled for apoptotic corpses using anti-Dcp-1 staining and for macrophages using anti-Crq staining. By taking a stack of images through a labeled macrophage, it is possible to count the number of Dcp-1–stained corpses inside one macrophage. The white dotted circles represent the individual macrophages. As suggested by the reviewer, we used green/magenta rather than green/red. We agree with the reviewer that by using srp-GAL4, UAS-GFP, it is easy to see where one cell begins and ends. Following the reviewer’s suggestion, we used the fly strain srp- gal4>UAS-GFP(BDSC#78565) to mark macrophages and stained corpses by anti- Dcp1. We found the apoptotic cell clearance phenotype, based on the phagocytic index (PI), was quite similar to our anti-Crq/anti-Dcp-1 staining assay (please see S5 Figure).
**Reviewer: This is reassuring. Thank you for this, and apologies for being a bit pedantic.**

**RESPONSE:** We are glad that our previous revisions addressed your concerns. Thank you again for the valuable feedback.

7.Figure 4d shows that Bfc is nuclear in the S2 cells. Can nuclear marker/dye and higher
magnification imaging show a similar localization in macrophages in the embryo? What
happens to Srp localization in the RNAi of Bfc from S2 cells? This should be fairly easy to do with the reagents already in hand and may provide insight into how the Bfc-Srp interaction works to regulate Crq levels (especially as I am not sure about the EMSA data).
RESPONSES: We agree with the reviewer’s comment. Hoechst 33342 was used to label the nucleus together with anti-Bfc and anti-Crq antibodies in vivo. We can confirm that Bfc is localized in macrophages but not the subcellular localization of Bfc, which may be due to the quality of our Bfc antibody or the limited resolution of laser confocal microscopy. We isolated macrophages from larvae and stained them with anti-Bfc antibodies to confirm that Bfc localized in the nucleus ( Figure 4B”). Moreover, we performed bfc RNAi transfection in S2 cells, but Srp location did not change when compared to the control ( Fig 4E-E').
**Reviewer: The anti-Bfc staining in B’’ is not very convincing and the Bfc-GFP
 localization in S2 cells is rather odd. Can the authors show a *BfcKO* mutant macrophage as a negative control and/or controls corresponding to pre-immune sera or no primary antibody control?**

**RESPONSE:** We agree with the reviewer. For accuracy, we used the *bfc^ko^* macrophages for anti-Bfc antibodies staining as a negative control and the results are shown in **Figure** **4B’’’**.

8. Why are immune genes, such as cecropin, in transcriptional profiling? This
seems counterintuitive to the silent clearance of apoptotic cells, as it suggests immune
activation. We have had issues generating apopototic cell samples via similar
methods – we obtained apoptotic cells and a large number of necrotic cells. The AC
samples used/method therefore require characterisation/validation (e.g., caspase activity probes, annexin, PI – all widely available). Therefore, it is possible that some of the upregulated genes are in response to necrotic cells present in the samples, in addition to the apoptotic ones. The AC samples should be characterized; if necrotic cells are present, the conclusions need to be modified to acknowledge that the transcriptional changes observed may not be specific to contact with apoptotic cells.
RESPONSES: We thank the reviewer for this insightful suggestion. Because of the anti-inflammatory nature of the apoptotic process, factors released from apoptotic cells should suppress inflammation. However, some studies have shown that some signals from ACs activate macrophage inflammation. We have added this to the Discussion section (please see page 18, line 493-499). As instructed, we validated the AC samples added to live S2 cells and showed that most of them were apoptotic rather than necrotic cells (please see S1A-S1C Figure). However, we cannot eliminate the upregulated expression of genes that may be induced by the presence of a few necrotic cells, so we have modified the conclusions to acknowledge that the transcriptional changes observed may not be specific to contact with apoptotic cells (please see page 18, line 485-490).
**Reviewer: I am satisfied with these changes. However, at the start of the sentence on line 485 can be deleted. The legend should state which %/quadrant relates to the AC.**

**RESPONSE:** We are glad that our previous revisions addressed your concerns. Thank you again for the valuable feedback. Of note, the manuscript was edited by a professional scientific English language editor that suggested alterations to improve the readability and flow of the text.

9. In general, the discussion needs to discuss the findings in a broader context, as it mostly simply repeats the conclusions/findings of this paper. In particular there is barely any discussion of the work in relation to other organisms (only 1 mammalian paper is cited). For instance, do homologs of Bfc exist in vertebrates? Is this a fly specific mechanism? A careful re-write of this section will make it much more appealing and relevant for non-Drosophilists.
RESPONSES: We appreciate the reviewer for the helpful and constructive suggestion. Accordingly, we have now provided expanded the Discussion section per your suggestions in the revised manuscript.
**Reviewer: This is much improved.**

**RESPONSE:** We are glad that our previous revisions addressed your concerns. Thank you again for the valuable feedback.

Minor points
1. Figure 1d-e What does % phagocytosis mean? I presume that% of S2 cells phagocytosing AC –please make clearer in the legend and methods.
RESPONSES: We apologize for not providing a clearer description of our apoptotic cell clearance assay in the previous draft. We have made it clearer in the legend and added the detailed method in the M&M section of the revised manuscript (please see page 24, lines 653-665).
**Reviewer: This has not been adequately explained. Is it the % of cells that phagocytose/efferocytosing AC? Please state this in your legend.**

**RESPONSE:** We apologize that our revision was not satisfactory. The ratio of double-labeled live cells (cell tracker blue and FITC-green) versus all live cells (cell tracker blue) was calculated as the percentage of efferocytosis **(lines 714-716)**. This definition is now included in the revised manuscript.

2.I am not sure that it is possible to make the conclusion that “as an engulfment receptor, Crq contributed to early recognition of ACs, while engulfment at later stages likely depended on other regulatory factors to mediate further AC clearance.” It is not clear whether the internalized ACs were internalized at earlier times and have not yet been degraded or have been internalized more recently. In my opinion, to test this properly, you would need to stimulate with AC, remove excess AC, and then challenge again, or something similar (if this was actually how the experiment was carried out, it is not clear from the results, methods, or figure legend). I do not propose that this experiment is repeated as suggested, as this does not seem to be a key point, just as the conclusions are modified.
RESPONSES: We agree with the reviewer that it is not clear whether the internalized
ACs were internalized at earlier times and were not yet degraded or were
internalized more recently. Accordingly, we have modified the conclusion (please see page 4-5, line 108-110, line 121-126).
**Reviewer: This has been addressed.**

**RESPONSE:** We are glad that our previous revisions addressed your concerns. Thank you again for the valuable feedback.

3.What are the experimental conditions for Figure 2c and d? This is not clear from the text,
methods, or legends. I presume this is with the AC treatment, but for how long?
RESPONSES: We apologize for not providing a clearer description of the experimental
conditions for Figure 2c, d. We have modified the Figure legend to make it clearer (please see page 7, line 188-194).
**Reviewer: Addressed but please carefully proof the legend(s) as there are some missing words that make it hard to read, for example, “adding ACs 6h” -> probably should read “adding ACs for 6h”**

**RESPONSE:** We thank the reviewer for the suggestion. We have carefully checked the legends and corrected the mistakes **(Fig 2C-2E)**. Please note that we also procured the services of a professional scientific English language editing company (Editage), to improve the readability and flow of the manuscript text. We believe that now the text, including the Figure Legends, reads better.

4.The effect of knockdown of CG9129/bfc is far stronger than that of crq itself (Figure 2c), and other processes may be affected given that bfc seems to play no role in SIMU and drpr. It may be possible to speculate on this in the discussion.
RESPONSES: We thank the reviewer for this useful suggestion. The effect of
knockdown of CG9129/bfc was far stronger than that of crq itself, which may be caused by the different RNAi knockdown efficiencies of bfc and crq. We agree with the reviewer that although there were no effects on drpr and simu expression in bfcko mutants, other processes may be affected by bfc, such as the clearance of neuronal debris and bacterial clearance. We have accordingly indicated this in the Discussion section.
**Reviewer: Now included.**

**RESPONSE:** We are glad that our previous revisions addressed your concerns. Thank you again for the valuable feedback.

5.Figure 3a appears after Figure 3c-d. The authors may want to reorder the text/figure slightly to improve how the manuscript reads. There are quite a few panels not cited (e.g., Figure S3e) in the text or mistakes in which figures are being cited throughout the manuscript.
RESPONSES: As instructed by the reviewer, we have revised Figure 3 in the text (please see page 8, line 205-208) and corrected the mistakes in citing the figures.
**Reviewer: Addressed.**

**RESPONSE:** We are glad that our previous revisions addressed your concerns. Thank you again for the valuable feedback.

6.Not all of the inset can be seen in the main field of view for the central panel of Figure 3a, while the zooms could have been slightly larger. I also struggled to see where the regions were taken from (in fact, I am unable to see where the zoom for bfc RNAi came from at all for that field of view).
RESPONSES: Thank you for this useful suggestion. The magnified regions are indicated by the white dotted squares in Figure 3A.
**Reviewer: The white box for bfc RNAi is not quite in the right place, and the brightness of the zoomed regions is different from that of the main panel. The authors may want to address this to be consistent/make their manuscript as robust as possible.**

**RESPONSE:** We understand the reviewer’s point. We looked at the zoomed regions under 40× magnification; please note that the original image was observed under the 20× objective, so the fluorescence may have appeared different.

7.Please provide details of how the mouse anti-Bfc was generated (what epitope was used etc). Authors should check for other methods that are not described in the paper.
RESPONSES: As instructed by the reviewer, we have provided details of how the mouse anti-Bfc antibodies were generated to the M&M section (please see pages 25-26, lines 691- 700). We have also rephrased our M&M section.
**Reviewer: Thank you.**

**RESPONSE:** We are glad that our previous revisions addressed your concerns. Thank you again for the valuable feedback.

8. Figure 4a - why is the green channel so bright in the bfc[ko] embryo? Have these embryos been imaged using the same settings/enhanced similarly? Why is the green channel so faint in the boxed region of the bfc[ko] panel compared to the main image? Individual channels should be shown and advisable to use magenta in place of red for merges. Also, it would be better not to inset zoomed regions over the posterior of the embryo as this prevents the reader from analysing expression in posterior macrophages.
RESPONSES: We apologize for not providing a clearer description of experimental
conditions for Figure 4a in the original manuscript. We used Crq staining intensity to show the macrophages in the bfcko embryo. Now, we have imaged WT and bfcko embryos under the same settings and used magenta in place of red to show individual channels as well as merges. As instructed by the reviewer, we have put the zoomed regions in the upper right corner of each embryo to avoid covering the posterior macrophages (please see Figure 4A).
**Reviewer: Thank you.**

**RESPONSE:** We are glad that our previous revisions addressed your concerns. Thank you again for the valuable feedback.

9.References required for this statement on p8: “Previous researches have demonstrated that several signal pathways regulated the expression of engulfment receptor Drpr, such as d-JNK, PI3K and insulin.”
RESPONSES: We thank the reviewer for reminding us of this point. We have added the
corresponding references in the revised manuscript (please see page 12, lines 320- 321).
**Reviewer: Thank you.**

**RESPONSE:** We are glad that our previous revisions addressed your concerns. Thank you again for the valuable feedback.

10. page 10 – some confusion / mix ups for figure labelling for Figures S5-7. Not all panels are cited in the text.
RESPONSES: We apologize for these mistakes. We have corrected the text in page 10 for S5 Figures (now it’s S7 Figure, please see page 14-15).
**Reviewer: Thank you.**

**RESPONSE:** We are glad that our previous revisions addressed your concerns. Thank you again for the valuable feedback.

11. Why does bfc not work to regulate the amounts of other Srp-dependent receptor genes? Authors suggest other co-factors regulate Srp’s activity at the drpr and simu promoters but I don’t think their model is sufficient to explain why Bfc would not assist at those promoters given their regulation by Srp. Perhaps it would be clearer with more explanation?
RESPONSES: Thank you for this insightful remark. The transcriptional factor Serpent (Srp) was found to be necessary for sufficient phagocytosis of ACs in Drosophila embryonic macrophages by regulating SIMU, Drpr and Crq (Shlyakhover E et al., 2018, Front Immunol 9:266). However, there is no evidence that Srp directly regulates the expression of SIMU, Drpr and Crq. Moreover, we did not detect the binding of Srp to drpr promoter (Please see S9 Figure). Another explanation is that Bfc would assist Srp at the promoter regions of simu and drpr, but exert minor effects, thus we could not detect the effect in bfc mutant in our study. We have added more explanation regarding this in the discussion part (please see page 19, lines 521-525).
**Reviewer: Addressed.**

**RESPONSE:** We are glad that our previous revisions addressed your concerns. Thank you again for the valuable feedback.

12.P13 “These results imply that the expression of phagocytotic receptors could be stimulated by the presence of excessive ACs to improve the phagocytotic activity of macrophages.” This has been shown by a number of other labs in the fly (e.g., Wood and McCall) in flies and is well-documented in vertebrates. These and other papers should be cited and this new mechanism contrasted with how this is regulated in other systems.
RESPONSES: We appreciate the reviewer for pointing out these mistakes and apologize for not citing the findings from other scientists who have reported in this field. We have now cited their works and discussed their mechanism in our revised manuscript (please see page 19, line 508-514).
**Reviewer: Addressed.**

**RESPONSE:** We are glad that our previous revisions addressed your concerns. Thank you again for the valuable feedback.

13. Authors may wish to include Han et al. (2014) and Guillou et al. (2016) in the discussion since these suggest functions for Crq other than a role as a receptor for apoptotic cells.
RESPONSES: We thank the reviewer for reminding us of this point. we have now cited the work by Han and Guillou works in our revised manuscript (please see page 20, line 554).
**Reviewer: Thank you.**

**RESPONSE:** We are glad that our previous revisions addressed your concerns. Thank you again for the valuable feedback.

14. Supplemental tables 3 and 4 are not cited in the manuscript.
RESPONSES: We apologize for this mistake. We have now cited S3 Table and S4 Table in the M&M section (please see page 25, line 671 and page 26, line 696).
**Reviewer: Thank you.**

**RESPONSE:** We are glad that our previous revisions addressed your concerns. Thank you again for the valuable feedback.

Reviewer 3

Bfc, anovel Serpent co-factor for the expression of Croquemort, regulates efferocytosis in Drosophila melanogaster
Thank you very much for providing me with a lined manuscript!
I see the authors have met the main criticisms, concerns and suggestions of the first review. At some points in the revised manuscript, however, further explanations have to be provided in order to make the results clearer. The manuscript is now clearly written, but the linguistic expression can be further improved and please check again the use of abbreviations especially in headings. This can certainly be compensated by some minor revisions that are listed below.

**RESPONSE:** First of all, we thank the reviewer for the careful revision of our manuscript, and for the constructive feedback. Your suggestions have helped us to considerable improve our manuscript, and for that we are deeply grateful. Once again, we have considered all of the new comments. Please check below our point-by-point responses to them.

Author summary
Line 32: “. such…”

**RESPONSE:** We thank the reviewer for the suggestion. We have corrected the grammatical mistake.

Introduction
Line 74: “Drosophila; …”

**RESPONSE:** We thank the reviewer for the suggestion. We have corrected the grammatical mistake.

RNAseq identified 48 genes with similar expression patterns to crq, which was
upregulated after incubation with ACs.
Line 102-103: crq and ACs!

**RESPONSE:** We thank the reviewer for the suggestion. We have corrected the grammatical mistake**(line 104-105)**.

Line 153: The numbers in the Venn diagram differ from the information in the Figure Legend. In addition, there was a correction that should be shown on lines 141-142, but this line only shows the title of the figure legend.

**RESPONSE:** We considered all grammatical changes suggested by you while revising our manuscript. Additionally, we availed the services of a professional Editing company (Editage), to improve the readability and flow of the manuscript text. Regarding the numbers in the Venn diagram, they represent the genes only expressed in one sample together with the genes expressed by two samples; on the other hand, the numbers in the Figure legend represent the sum of the differentially expressed genes in each sample.

Line 141: “The significantly upregulated…”

**RESPONSE:** We thank the reviewer for the suggestion. Our manuscript was edited by a professional scientific English language editor for the improvement of the readability and flow of the text. This sentence was revised having in mind your feedback, for accuracy.

Bfc is involved in efferocytosis and regulates crq expression in S2 cells.
Line 184: Please avoid abbreviations as in the chapter heading when Bfc is used for the first time, the same as crq in the previous chapter heading.

**RESPONSE:** We thank the reviewer for the suggestion. For accuracy, we have changed Bfc to CG9129, as it was used for the first time.

The expression of Crq is mediated by Bfc in vivo.
Line 253-254: Please rephrase or explain.

**RESPONSES:** We thank the reviewer for the remark. We have updated the original description as follows: “Confocal stacks of the head region of stage 13 embryos on the ventral view. Macrophages and apoptotic bodies were stained with anti-Crq (green) and anti-Dcp1 (magenta) antibodies, respectively in wild-type, *bfc^ko^* mutant, and *bfc^ko^*; crqGal4>UAS-Bfc embryos.”

Line 256: …blot analysis of Crq protein levels… please add also Bfc!
Please correct the heading for Supplement 3.

**RESPONSES:** We thank the reviewer for the suggestion. The Figure heading was revised in line with your remark. Additionally, we mentioned Bfc in the Legend of **Figure 3H.**

bfc is required in macrophages for efficient efferocytosis.
Line 282: Please explain the Result of the newly designed Figure S5 and not only mention it.

**RESPONSE:** We thank the reviewer for the remark. Now we have explained Figure S5 in detail in the revised manuscript **(lines 290-297)**.

Line 299: meanwhile

The hint to Figure 3J is missing!

**RESPONSE:** We thank the reviewer for the remark. Now, Figure 3J was cited appropriately in the revised manuscript **(line 299)**.

Bfc physically interacts with GATA factor Srp.
Line 304: Bfc is expressed…

**RESPONSE:** We thank the reviewer for the suggestion. Our manuscript was edited by a professional scientific language editor for the improvement of the readability and flow of the text. This sentence was revised per your suggestion **(line 350-351)**.

Line 307: “premised it was a transcription factor” please rephrase “premised it encodes a transcription factor”

**RESPONSE:** We thank the reviewer for the suggestion; we have updated this sentence in accordance **(line 324)**.

Line 312: “…examine the expression location of Bfc…” please rephrase “protein localization”

**RESPONSE:** We thank the reviewer for the suggestion. Our manuscript was edited by a professional scientific language editor for the improvement of the readability and flow of the text. This sentence was revised for better readability **(line 331-333)**.

Line 317: Fig 4B´-B´´ please change because B´ and B´´ are not similar as B! The same for E and E´ in Fig. 4.

**RESPONSE:** We thank the reviewer for the remark. The manuscript was updated accordingly **(lines 333-334 and line 346-347)**.

Line 345: Please insert “as well as” after control S2 cells!

**RESPONSE:** We thank the reviewer for the suggestion; the sentence was updated accordingly **(line 362)**.

Bfc acts as a co-factor for the Srp transcription factor to regulate Crq expression and efferocytosis.
---
Srp directly binds the crq promoter through GATA site recognition enhanced by Bfc.
Line 403-404: …that only Srp-AD interacted only with the crq…

**RESPONSE:** We thank the reviewer for the suggestion. The sentence has been revised to reflect your suggestion **(lines 423-424)**.

Line 408-409: …mutated Srp Znf from Cys to Arg eliminated binding to the crq promoter (S7D and S7F Fig). This is not shown in S7f due to only binding of Srp-AD to the crq promoter!

**RESPONSE:** We thank the reviewer for the suggestion.We performed the Y1H assay in the context of both Srp-AD and Srp C4^M^-AD (with the mutated ZnF of Srp) binding to the *crq* promoter; importantly, Srp C4^M^-AD did not bind to the *crq* promoter suggesting that the DNA-binding motif is important for the interaction between Srp and crp **(Figure S7F)**.

Line 412-414: Our results showed that the ability of crqM-Gal4 to activate the transcription of crq was much weaker than crq-GAL4“
This statement sounds to me like the failure of a specific transcriptional activation of crq due to the mutated GATA site. But due to the mutated GATA site, all other UAS constructs such as unspecific UAS-lacZ or UAS-GFP constructs cannot be activated either, right?

**RESPONSE:** We thank the reviewer for these insightful remarks. As an alternative approach, we derived a *UAS-GFP* construct and detected the GFP levels in the context of *crq-Gal4/UAS-GFP*, and *crq^M^-Gal4/UAS-GFP*. A weaker GFP signal was detected in the context of *crq^M^-Gal4/UAS-GFP*, suggesting that *crq^M^-Gal4* can activate the unspecific protein **(S9A-C Fig)**.

Line 414: …in bfc also has an insufficient activation to crq… Please rephrase to …in bfc also leads to insufficient activation of crq…

**RESPONSE:** We thank the reviewer for this suggestion. We have updated the sentence for better readability **(lines 433-435)**.

Line 421: Please correct the red arrow in Fig. 6A to the indicated red arrowhead or vice versa.

**RESPONSE:** We thank the reviewer for the suggestion. We have used black arrows instead to denote the primer regions. The respective figure legend was updated accordingly **(Figure 6A)**.

Line 430: E´ Please change because E´ is not the same as E!

**RESPONSE:** We thank the reviewer for the suggestion. The respective figure legend was updated accordingly **(Figure 6F and 6F’)**.

Line 439-441: HA-Srp… Can you explain or please rephrase?

**RESPONSE:** We thank the reviewer for the suggestion. We meant S2 cells expressing HA-Srp. Please note that these cells were generated via transfection with the pAC5.1-HA-Srp construct; all constructs used in this study are listed in the S4 Table to ensure our experiments can be reproduced by others.

Line 445-447: Our results showed… Can you explain the results of Figure 6F in more detail or please rephrase?

**RESPONSE:** We thank the reviewer for the suggestion. Accordingly, we have explained **Figure 6E** in more detail, in the Results section of the revised manuscript **(lines 468-475)**.

Discussion
Line 528: Meanwhile?

**RESPONSE:** The text was edited by a professional scientific language editor for better readability and flow.

Material & Methods
Line 776- 778: This technique… avoid such sentence please.

**RESPONSE:** We thank the reviewer for the suggestion. We have changed this sentence in the revised M&M section, accordingly.

Supplemental Information
Line 1125: S1 Fig. ACs were successfully induced… Please check for abbreviations used for the first time!

**RESPONSE:** We thank the reviewer for the suggestion. And we checked that the abbreviations accured in the introduction firstly.

Line 1128-1129: …as described in the method.

**RESPONSE:** We thank the reviewer for the suggestion. And we have corrected the legend**(line 1080-1082)**.

Line 1174-1175: S4 Fig. Title? Please indicate used arrowheads and indicate all micrometer steps. Please explain the newly designed Figure in the Results section and not only as a hint to S5.

**RESPONSE:** We thank the reviewer for the suggestion. We have changed the S4 Fig title to “*CG9129* knockout did not affect the number of macrophages and ACs in the embryos”, and we have rewritten the corresponding result**(line 285-288)**.

Line 1206-1207: Yeast two-hybrid shows the interaction between Bfc and C4 M of Srp.

**RESPONSE:** We thank the reviewer for the suggestion. We have changed the sentence**(line 1161-1162)**.

Line 1209: …interaction between C4 M of Srp and crq promoter. Please indicate „C4 M of Srp“ in S7F.

**RESPONSE:** We thank the reviewer for the suggestion. We have changed the sentence**(line 1162-1163)**.

Line 1211: Please correct the heading of S7 to: “…of UAS-Crq expression in Drosophila embryos.” Since only expression patterns of effector lines are traced!

**RESPONSE:** Thank you for the remarks. Most of the above points were considered in the revision of our manuscript.

English
Please check again the linguistic expression all over the manuscript.

**RESPONSE:** We thank the reviewer for the suggestion. We procured the services of a professional English language editing company (Editage), to improve the readability and flow of the manuscript text. We believe now the text is written in a clearer and consistent way, and the grammatical inconsistencies were overall corrected.
