# Supplementary material for: bfc, a novel serpent co-factor for the expression of croquemort, regulates efferocytosis in Drosophila melanogaster
Source: PLoS Genet. 2021 Dec 3;17(12):e1009947. doi: 10.1371/journal.pgen.1009947 (PMC8673676; doi:10.1371/journal.pgen.1009947)
Supplement: Attachment — Submitted filename: response_to_reviewers_21.11.08.docx [file pgen.1009947.s023.docx]

**Reviewer #1:** The authors significantly improved their manuscript and addressed my main concerns.

**RESPONSE:** We are glad that our previous revisions addressed your concerns. Thank you again for the valuable feedback.

I may have missed it but I didn't find an accession number to a public repository for the RNA-seq data. This should be provided.

**RESPONSE:** We thank you for kindly reminding us about this. We have shown the accession number of the raw RNA-seq data in the M&M section **(lines 687-688)**.

**Reviewer #2:** The authors have satisfied all my comments and (in my opinion) have done a good job of answering the other reviewers's comments.

The manuscript is now far easier to read and the conclusions are more robust with the additional new data. The figures are much improved and more robust. Areas in which the paper was less strong have now been significantly improved (methods, presentation of gels/legends/descriptions, statistics).
Some typos remain and the manuscript should be proofed carefully. I list some errors I found while re-reading the manuscript to help the authors (see below); It may be that the editing company changed the meaning a little in some places.
This is really interesting work and I look forward to hearing about the other hits they have uncovered via this approach.

**RESPONSE:** We are glad that our previous revisions addressed your concerns. Thank you again for the valuable feedback. Once again, we have considered all the new comments. Please check our point-by-point responses below.

Figure 4B-B’’’ – I suggest authors add annotation to these panels to indicate S2 cell / S2 cell / wt mac / bfcKO mac to help the reader.

**RESPONSE:** We apologize for not providing a clearer description of the experimental
conditions for Figure 4B-B’’’. We have added an annotation to these panels and have modified the Figure legend to make it clearer **(Fig 4B-B’’’, lines 357-359)**.

Line 308 – says authors generated the insertion line, when materials and methods says this was obtained from Bloomington.
**RESPONSE:** We apologize for the vague description of the source of *bfc^MI02020^*. We have corrected the text **(lines 311-312)**.

Line 336 “Bfc act as…” should read “Bfc acts as…”
**RESPONSES:** We apologize for the mistake. We have corrected the text **(line 339).**

Line 490 Misspelling - Bfs rather than Bfc
**RESPONSES:** We apologize for the mistake. We have corrected the text **(line 499).**

Line 581 indicate rather than indicating
**RESPONSES:** We apologize for the mistake. We have corrected the text **(line 590).**

Line 783 TRITC rather than RITC?

**RESPONSES:** We apologize for the mistake. We have corrected the text **(line 782).**

**Reviewer #3:**

Bfc, a novel Serpent co-factor for the expression of Croquemort, regulates efferocytosis in Drosophila melanogaster
I see the authors have met the main criticisms, concerns and suggestions of the first two reviews. The manuscript is now clearly written, but please check again the use of abbreviations especially in headings. In some places in the revised manuscript, however, further explanations are required to clarify the results. In addition, please check again the order in the material and methods section and the use of formal genetic nomenclature in the manuscript (except for the discussion) needs to be re-examined. This can certainly be compensated by some revisions that are listed below.

**RESPONSE:** First of all, thank you for the time spent reviewing our manuscript. Your feedback has helped us improve our manuscript considerably, and we are deeply grateful for that. We have changed the order in the material and methods section according to your suggestion. And once again, we have considered all new comments. Please check our point-by-point responses below.

Title page
Line 1 and 12: Bfc=bfc, Serpent=serpent Croquemort=croquemort, Bfc=bfc
Possibly because of the title page, but formally incorrect.

**RESPONSES:** We apologize for these mistakes. We have corrected the title according to your suggestion.

Abstract
Line 16: in other words

**RESPONSES:** We thank the reviewer for the remark. We have deleted the words in the abstract.

Line 21: Bfc (booster for croquemort) = bfc (booster for croquemort)! Here the novel gene is described. Please always use lowercase and italic notation, while uppercase and non-italic notation have to be used for the corresponding protein.

**RESPONSES:** We apologize for these mistakes. We have corrected the format for the gene names **(line 22)**.

Key words
Line 31-32: Alphabetical order?

**RESPONSES:** We thank the reviewer for the remark. We have changed the keywords and have arranged them in alphabetical order **(lines 32-33)**.

Authors summary
Line 40: Lupus

**RESPONSES:** We apologize for the mistake. We have corrected the word **(line 40)**.

Line 42-43: Sentence construction?

**RESPONSES:** We apologize for not providing a clearer description. We have rephrased the sentence **(lines 43-44)**.

Line 44-45: gene: bfc (Booster for Croquemort) = bfc (booster for croquemort)

**RESPONSES:** We apologize for the mistake. We have corrected the format of the gene name **(lines 45-46)**.

Introduction
Line 56: whereby apoptotic cells…

**RESPONSES:** We thank the reviewer for the remark. We have corrected the text **(line 57).**

Line 80: …seemed…

**RESPONSES:** We apologize for the mistake. We have corrected the text **(line 81).**

Line 88: the transcriptional factor…

**RESPONSES:** We apologize for the mistake. We have corrected the text **(line 89).**

Line 95: gene: bfc (booster for croquemort)

**RESPONSES:** We apologize for the mistake. We have corrected the text **(line 96).**

Results
RNAseq discloses 48 genes with expression patterns comparable to that of crq in S2 cells co-incubated with ACs
Line 106: Here

**RESPONSES:** We thank the reviewer for the remark. We have corrected the text **(line 107).**

Line 118: above mentioned…

**RESPONSES:** We apologize for the mistake. We have corrected the text **(line 119).**

Line 146: AC efferocytosis…

**RESPONSES:** We apologize for the mistake. We have corrected the text **(line 147).**

CG9129 is involved in efferocytosis and regulates crq expression in S2 cells
Line 184: Protein: Booster for crq (Bfc)

**RESPONSES:** We apologize for the mistake. We have corrected the text **(line 185).**

crq expression is regulated by Bfc in vivo
Line 210: Crq (mRNA or protein?) for me it seems crq expression?

**RESPONSES:** We apologize for the vague description. Crq here is the protein because the expression was shown by immunostaining in the cited reference **(line 211).**

Line 223: (Figure S3E) allover the manuscript it is (S3E Fig) and also include (S3D-S3E Fig)

**RESPONSES:** We apologize for the mistake. We have corrected the text **(line 224).**

Line 229: Please explain use of H99 embryos as in the corresponding Figure legend

**RESPONSES:** We apologize for the vague description. We have corrected the text **(line 231).**

Line 231: …embryonic development…

**RESPONSES:** We apologize for the mistake. We have corrected the text **(line 233).**

Line 232: …Crq expression… mRNA or protein?

**RESPONSES:** We apologize for the mistake. “*crq* expression” here means mRNA, so we have changed Crq into *crq* **(line 234).**

Line 237: …expression of drpr and simu… Data not shown for simu?

**RESPONSES:** We thank the reviewer for the remark. Data for *simu* is shown in S4A Fig, which revealed that *bfc* did affect *simu* expression **(lines 248-249).**

Line 243-246: Moreover, we did not observe… Why moreover? And please explain the following part in more detail for better understanding!

**RESPONSES:** We apologize for the vague description, and we rephrased this part to make make it easy to understand **(lines 245-249).**

Line 261: Please change UAS-Bfc to UAS-bfc

**RESPONSES:** We apologize for the mistake. We have corrected the text **(line 264).**

Line 264: stage 13 embryos on the ventral view. As far as I can see, it is a lateral view

**RESPONSES:** We apologize for the mistake. We have corrected the text **(line 267).**

Line 266-267: These embryos… Please clarify!

**RESPONSES:** We apologize for the vague description. We have rephrased this sentence to avoid confusions **(line 269).**

Line 270: Please change UAS-Bfc to UAS-bfc

**RESPONSES:** We apologize for the mistake. We have corrected the text **(line 273).**

Macrophages require Bfc for efficient efferocytosis
Line 294-295: …both bfc…, while bfc…? Please explain

**RESPONSES:** We apologize for the mistake. We have corrected the text **(lines 297-300).**

Line 300, 301, 303: Bfc: all bfc?

**RESPONSES:** We apologize for the mistake. We have corrected the text **(line 303).**

Line 308: …we generated a mutant with a MiMIC insertion… But you write in the M&M section that you got the mutant from the Bloomington stock keeping center. Please clarify!

**RESPONSE:** We apologize for the vague description of the source of *bfc^MI02020^*. We have corrected the text **(lines 311-312)**.

CG9129 physically interacts with GATA factor Srp
Line 322: Italics for CG9129 and Srp in the context of physical interaction?

**RESPONSES:** We apologize for the mistake. We have corrected the text **(line 325).**

Line 330-331: …revealed a strong Bfc expression protein localization in macrophages…

**RESPONSES:** We apologize for the mistake. We have corrected the text **(lines 333-334).**

Line 350-351: Title of the figure legend seems not appropiate to me due to protein extracted from S2 cells and not from embryonic hemocytes as stated in the heading

**RESPONSES:** We agree with the reviewer’s comment. We have modified the title of Fig 4 to make it more appropriate **(lines 353-354)**.

CG9129 and Srp act together to regulate Crq expression in context of efferocytosis
Heading: Spelling of CG9129, Srp and Crq expression? …the context…?

**RESPONSES:** We apologize for the mistake. We have corrected the heading **(lines 369-370).**

Line 371: …bfc/srp mutant double heterozygous embryos

**RESPONSES:** We apologize for the mistake. We have corrected the text **(line 375).**

Line 374: …exhibited similar defects… Please also explain the defects!

**RESPONSES:** We agree with the reviewer’s comment. We have added the PIs of *bfcko* and *srp* to explain the defects **(lines 378-379)**.

Line 382: crq/bfc double heterozygous mutants

**RESPONSES:** We apologize for the mistake. We have corrected the text **(line 387).**

Line 384: …and phagocytic defects as those observed in… Please explain the defects!

**RESPONSES:** We agree with the reviewer’s comment. We have added the PIs of *bfcko*/*crqko* to explain the defects **(lines 389-390)**.

Line 388: Spelling of CG9129?

**RESPONSES:** We apologize for the mistake. We have corrected the text **(line 394).**

CG9129 enhances direct binding of GATA factor Srp to the crq promoter
Heading: Spelling of CG9129 and Srp?

**RESPONSES:** We apologize for the mistake. We have corrected the heading **(lines 415-416).**

Line 430: …we mutated the Srp-binding site in the GATA sequence and integrated this construct, or the crqM-Gal4 construct as a control…? Maybe: we mutated the GATA sequence in the Srp-binding site and integrated this crqM-Gal4…

**RESPONSES:** We thank the reviewer for this suggestion. We have corrected the text according to your suggestion **(lines 436-437).**

Line 435: Meanwhile?

**RESPONSE:** We thank the reviewer for these insightful remarks. We have updated this part for better readability **(lines 441-446)**.

Line 435-438: Can you please rephrase for better understanding

**RESPONSE:** We thank the reviewer for these insightful remarks. We have updated this part for better readability **(lines 441-446)**.

Line 438-444: Please also refer to the corresponding Figures

**RESPONSE:** We thank the reviewer for the remark. Now, Figures 6C and 6D are cited appropriately in the revised manuscript **(line 449)**.

Line 432-437: Please refer also S8F

**RESPONSES:** We apologize for the mistake. We have corrected the text **(line 441).**

Line 471: …bfc and or srp…

**RESPONSES:** We apologize for the mistake. We have corrected the text **(line 479).**

Line 477: …Srp proteins…

**RESPONSES:** We apologize for the mistake. We have corrected the text **(line 485).**

Line 479: …labeled crq… mRNA or protein?

**RESPONSES:** We apologize for the mistake. Here we are referring to the DNA. We have revised the text accordingly **(line 487).**

Line 486-491: Please refer to corresponding subfigures S10 A-D not only S10

**RESPONSES:** We apologize for the mistake. We have corrected the text **(line 497).**

Discussion
Line 558: Please change CO-IP to Co-IP

**RESPONSES:** We apologize for the mistake. We have corrected the text **(line 567).**

Line 573: As a potential Srp cofactor…

**RESPONSES:** We thank the reviewer for the suggestion. We have corrected the text **(line 582).**

Line 579- 582: Also… Please rephrase sentence for better understanding

**RESPONSE:** We thank the reviewer for this suggestion. We have rephrased this part for better readability **(lines 588-592)**.

Material & Methods
Line 640: Please change vasa Cas9 to vasa Cas9

**RESPONSES:** We apologize for the mistake. We have corrected the text **(line 719).**

Line 643, 644,648: Change gal4 to Gal4

**RESPONSES:** We apologize for the mistake. We have corrected the text **(lines 722, 723, 727).**

Line 655: Please change UAS-Bfc/UAS-CrqFlag to UAS-bfc/UAS-crqFlag

**RESPONSES:** We apologize for the mistake. We have corrected the text **(line 734).**

Line 709: Please change …away from the light to …in the dark

**RESPONSES:** We thank the reviewer for this suggestion. We have modified the text **(line 667).**

Line 735: …according to the report of…

**RESPONSES:** We thank the reviewer for this suggestion. We have modified the text **(line 696).**

Figures
Figure 4: Please indicate in B´-B´´´ S2/S2/Mp/Mp for better understanding

**RESPONSES:** We apologize for not providing a clearer description of the experimental conditions for Figure 4B-B’’’. We have added an annotation to the figure panels and have modified the Figure legend to make it clearer **(Fig 4B-B’’’, lines 357-359)**.

Supplemental Figures and Information
Line 1080: Please change …annexin to …Annexin V-FITC

**RESPONSES:** We apologize for the mistake. We have corrected the text **(line 1095).**

Line 1081: Bars and … aActinomycin D

**RESPONSES:** We apologize for the mistake. We have corrected the text **(line 1096).**

Line 1100: Figure title indicates expression in vivo but G and H display mRNA levels in S2 cells

**RESPONSES:** We thank the reviewer for this suggestion. We have modified the Figure title **(line 1105).**

Line 1121: Please rephrase Figure title

**RESPONSES:** We thank the reviewer for this suggestion. We have modified the Figure title **(lines 1136-1137).**

S4C Please indicate bars 3+4

**RESPONSES:** We thank the reviewer for this suggestion. We have added macrophages number to the figure panels and have corrected the text **(lines 1142-1144).**

Line 1130: Please rephrase Figure title and change UAS-GFP to UAS-GFP

**RESPONSES:** We thank the reviewer for this suggestion. We have changed the Figure title and corrected the text **(line 1146).**

Line 1140-1141: The crq and bfc mRNA levels were quantified by qPCR using wild-type and bfcMI02020 cells. Can you please explain what does cells mean?

**RESPONSES:** We apologize for the mistake. We have corrected the text **(line 1157).**

Line 1144, Line 1147: Please change UAS-Bfc and UAS-Crq to italics

**RESPONSES:** We apologize for the mistake. We have corrected the text **(line 1160, 1163).**

Line 1149: Please change E to F

**RESPONSES:** We apologize for the mistake. We have corrected the text **(line 1165).**

Line 1151-1152: The white arrowheads point to... There is no arrowhead in F S9D Please correct Srq to Srp

**RESPONSES:** We apologize for the mistake. The white arrowheads should be white triangles; we have corrected the text **(line 1167).**
